# PERK orchestrates an endoplasmic reticulum stress alternative splicing program via CLK1/SRSF1

**DOI:** 10.1038/s41467-026-74397-y

**Published:** 2026-06-19

**Authors:** Céline Philippe, Shoshana Burke, Arantxa Carrasco-Leon, Andrea Martisova, Pedro Casado, Eleni Maniati, Jimena Castorena, Pantelitsa Protopapa, Alyssa Fronk, Ru-Pin Alicia Chi, Rachel Boniface, Vinothini Rajeeve, Martin Dodel, Beatriz Galvão, Marc Aubry, Kaliya Svetlinova Georgieva, Doriana Di Bella, Brian N. Papas, Taylor Floyd, Kendall Anderson, Martin Akerman, Jun Wang, Lovorka Stojic, Faraz Mardakheh, Marcos Morgan, Eric Chevet, Christian Touriol, Pedro R. Cutillas, Kevin Rouault-Pierre

**Affiliations:** 1 Barts Cancer Institute, Queen Mary University of London, London, UK; 2 INSERM U1242, University of Rennes, Centre de Lutte contre le Cancer Eugène Marquis, Rennes, France; 3 INSERM U1342, Equipe Leader Fondation ARC 2024, IRSL, St Louis Hospital, University of Paris-Cité, Paris, France; 4 Envisagenics Inc., Queens, NY USA; 5 Reproductive and Developmental Biology Laboratory, National Institute of Environmental Health Sciences, National Institutes of Health, Durham, NC USA; 6 Department of Biochemistry, University of Oxford, Oxford, UK; 7 Integrative Bioinformatics, Biostatistics and Computational Biology Branch, National Institute of Environmental Health Sciences, National Institutes of Health, Durham, NC USA; 8 INSERM U1037, Centre de Recherches en Cancérologie de Toulouse, Toulouse, France; 9 Instituto de Parasitología y Biomedicina López-Neyra, Consejo Superior de Investigaciones Científicas (CSIC), Granada, Spain; 10 Leukemia Institute Paris Saint-Louis, Paris, France

**Keywords:** Endoplasmic reticulum, RNA splicing

## Abstract

The unfolded protein response (UPR) is a critical adaptive program triggered upon cellular stresses that profoundly reshapes the transcriptome and translatome. In the very first minutes of cellular stress, translation blockage, RNA decay and RNA granules formation prompt the synthesis of proteins essential to the stress response. Due to the dynamic nature of these processes, investigating translation upon stress has proven to be challenging; therefore, our understanding of these mechanisms and translatome rewiring upon stress remains limited. Here, we exploit O-Propargyl-puromycin (OPP) labelling of de novo peptides followed by LC-MS/MS to identify de novo proteins translated upon endoplasmic reticulum (ER) stress. Combined with transcriptomic analyses, our approach reveals that ER stress profoundly impacts the synthesis of core splicing factor proteins leading to a significant reshaping of the splicing landscape. We identify a signature of seven splicing events consistently occurring in mammalian cells exposed to ER stress. Using pharmacological, genetic, phosphoproteomic and sequencing approaches, we demonstrate that this specific signature is driven by PERK activation and is dependent on the axis CLK1/SRSF1. Our findings identify PERK/CLK1/SRSF1 -mediated splicing regulation as a new facet of ER stress, defining an ER^i-splice^ signature spanning healthy and malignant tissues.

## Abstract (web summary)

ER stress governs protein homoeostasis through the unfolded protein response. Here, the authors show that ER stress not only alters protein synthesis but also rapidly changes the proteome via a PERK-driven alternative splicing program, defining a conserved splicing signature across tissues.

## Introduction

In recent years, the Unfolded Protein Response (UPR) has emerged as a pivotal cellular process, primarily recognized for its role in maintaining endoplasmic reticulum (ER) homoeostasis under stress conditions^1^. The UPR’s influence extends beyond protein folding and degradation, touching upon various aspects of cell biology^2,3^, including RNA decay through the Regulated IRE1α-dependent Decay pathway (RIDD), facilitation by IRE1α of translation of transcripts with 5’ terminal oligopyrimidine motifs^4^, and metabolism via ATF6^5–8^.

The protein kinase R (PKR)-like endoplasmic reticulum kinase (PERK) is a key sensor and mediator of the UPR, and functions as a critical adaptive response to mitigate the burden of protein folding within the ER lumen^9–11^. PERK-mediated phosphorylation of the eukaryotic initiation factor 2 alpha (eIF2α) at serine 51 leads to an overall reduction in cap-dependent protein synthesis^12^, while under chronic ER stress conditions, PERK and its downstream effector ATF4 can initiate apoptotic pathways through the induction of CHOP (C/EBP Homologous Protein)^13^.

Our study investigates the role of PERK in regulating alternative splicing, a process critical for expanding protein diversity and modulating gene expression. Alternative splicing (AS) involves the selective inclusion or exclusion of exons and introns, leading to the production of multiple mRNA isoforms from a single gene to expand the cell’s protein-coding catalogue^14,15^, while contributing to proteins’ specific spatio-temporal expression^16^. Splicing is frequently disrupted in human cancers; however, the exact ways in which spliceosomal components are harnessed and hijacked during cancer progression remain poorly understood^17,18^. Global splicing changes upon a given stress are poorly understood. The acidic pH in solid tumours core leads to discrete splicing changes^19^; heat shock stress promotes splicing during thermal stress recovery^20^ and hypoxia can regulate alternative splicing in many ways, from altered expression of RNA splicing factors to activation of signalling pathways that promote alternative splicing^21^. RNA-binding proteins are important for canonical and alternative splicing, many are considered oncogenes, e.g., the serine-arginine-rich splicing factor 1 (SRSF1), whose increased expression is sufficient to induce pancreatitis and accelerate KRAS^G12D^-mediated pancreatic ductal adenocarcinoma (PDAC)^22^.

Here, we unveil a novel dimension of the UPR, illustrating its capacity to trigger an alternative splicing program. This mechanism is conserved across a spectrum of cellular contexts, from normal to malignant states. Our approach employed an innovative puromycin pull-down technique followed by liquid chromatography-tandem mass spectrometry (LC-MS/MS) analysis to identify de novo synthesized proteins upon ER stress. This methodology allowed us to pinpoint proteins involved in the UPR pathway and, intriguingly, in RNA processing, highlighting a hitherto unknown intersection between ER stress and splicing regulation.

To shed light on this intricate relationship, we employed a multi-omics approach, encompassing RNA-sequencing, splicing analyses, proteomic and phosphoproteomic investigations, along with the utilization of both pharmacological and genetic strategies in order to untangle the role of ER stress in rewiring the splicing machinery. Our findings reveal the emergence of an ER splice signature (ER^i-splice^) upon ER stress induction, observable across various cell types, including primary human cells and different cell lines. Our results not only broaden our understanding of the UPR but also illuminate a conserved alternative splicing program, extending from healthy to malignant cells, governed by the UPR. This nexus between ER stress and splicing regulation opens new avenues for therapeutic interventions and enhances our comprehension of cellular resilience mechanisms under stress conditions.

## Results

### RNA-binding proteins and components of the spliceosome are translated upon the Unfolded Protein Response

**Fig. 1:**
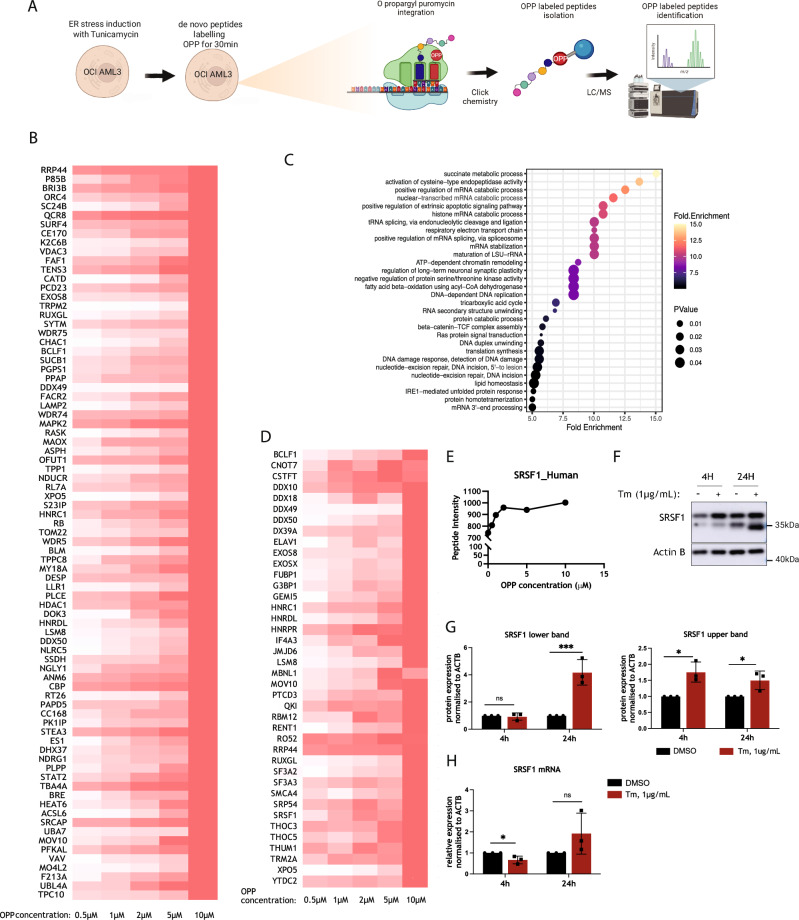
Proteins of the splicing machinery are translated in response to the unfolded protein response. **A** Schematic depicts the O-Propargyl Puromycin (OPP) pull-down method. OCI-AML3 cells are treated with tunicamycin at 1 μg/mL for 4H and co-treated with OPP for the last 30 min. Cells are lysed and, the click reaction between OPP and azide-beads is performed in vitro. Following pull-down, de novo peptides are identified by LC-MS/MS analysis. *Created in* *BioRender. Philippe, C.* (2026) https://BioRender.com/k977bw2. **B** Heatmap of de novo peptides captured with the OPP pull-down method in OCI-AML3 cells upon 1μg/mL tunicamycin treatment for 4H. Each facet depicts peptide intensity according to the OPP concentration. *Spearman coefficient* ≥0.8. **C**Gene ontology analysis shows terms enriched in the de novo peptides list captured upon UPR with the OPP pull-down method. one-tailed Fisher Exact Test. *p* < 0.01*, Fold enrichment* ≥ 5. **D** Heatmap of de novo peptides captured with the OPP pull-down method that are related to the RNA-binding term. Each facet depicts peptide intensity according to the OPP concentration. *Spearman coefficient* ≥0.8. **E** Graph depicts SRSF1 peptide intensity according to OPP concentration. OPP pull-down is performed in OCI-AML3 treated with 1 μg/mL tunicamycin for 4H. **F** Representative western blot of SRSF1 in OCI-AML3 cells treated with tunicamycin at 1 μg/mL for 4H or 24H. Actin B is used as a loading control. **G** Quantification of SRSF1 protein expression (left panel: lower band; right panel: upper band) in OCI-AML3 cells treated with tunicamycin at 1 μg/mL for 4H or 24H. Data here are normalized to Actin B (*n* = 3 biological replicates). mean± S.D. two-sided unpaired t-test. *p < 0.05, **p < 0.01, ***p < 0.005. **H** Quantitative PCR analysis of *SRSF1* mRNA in OCI-AML3 cells treated with tunicamycin at 1 μg/mL for 4 or 24H. Data here are normalized to Actin B (*n* = 3 biological replicates). mean± S.D. two-sided unpaired *t*-test. *p < 0.05.

To identify bona fide proteins de novo synthesised upon endoplasmic reticulum stress, we optimised a puromycin pull-down followed by proteomic analysis by LC-MS/MS. In short, de novo synthesised peptides are labelled with an analogue of puromycin, called O-Propargyl puromycin (OPP). The main advantage compared to other approaches, such as SILAC, AHA, BONCAT, is that OPP improved temporal resolution due to rapid incorporation^23^. The properties of this molecule allow conjugation of different partners through a click reaction. We optimised our protocol from earlier work from Forester et al., and other groups^24–27^. By linking OPP to azide-beads, de novo peptides can be pulled down and analysed by LC-MS/MS shortly after drug stimulation (Fig. 1A). Within 15-30 minutes of incubation in the culture media, the incorporation of puromycin can be detected by western blot and this incorporation can be prevented by the protein synthesis inhibitor, cycloheximide (CHX) (Supplementary Fig. 1A, B). We confirmed that upon 30 minutes treatment with OPP, neither UPR, nor integrated stress response (ISR), nor apoptosis (cleaved PARP), nor aggresomes formation (Proteostat staining) are triggered (Supplementary Fig. 1C, D).

In order to reveal de novo peptides synthesised upon ER stress, we incubated OCI-AML3 cell lines with tunicamycin (ER stress inducer) for three and a half hours prior to adding escalating doses of OPP to the media for thirty minutes (Fig. 1A). Our analysis revealed 350 proteins significantly correlated (Spearman coefficient ≥ 0.885) with OPP concentration upon ER stress, including previously reported proteins such as CHAC1^28^, G3BP1, SEC23IP, and ARIH1^29^ (Fig. 1B*;* Supplementary Data 1). This approach allows us to identify low-abundant proteins with high fold change (Supplementary Fig. 1E–G). Notably, ATF4 translation occurs within 30 minutes of ER stress and was already at a plateau when OPP was added (Supplementary Fig. 1H, I). Gene ontology analysis based on captured-de novo peptides (*p* < 0.01, Fold enrichment ≥5), shows an enrichment for protein synthesis, UPR pathway and more surprisingly pathways involved in RNA processing, such as mRNA splicing (e.g., SRSF1, HRNPR, ELAV1, QKI) (Fig. 1C, D, Supplementary 1J, K). For instance, the Serine and arginine-rich splicing factor 1 (SRSF1) is captured by LC-MS/MS (Fig. 1E) and by western blot (Fig. 1F, G), the top band on the WB representing the hyperphosphorylated active form of SRSF1^30^. In parallel, we confirmed that at 4 h the upregulation of SRSF1 is transcription-independent (Fig. 1H) and assessed the half-life of SRSF1 upon tunicamycin by performing a cycloheximide chase experiment in OCI-AML3 cells treated with 50 µg/mL cycloheximide. By monitoring the decay of both SRSF1 bands over time, we determined that SRSF1 has a half-life of 8 h under cycloheximide and tunicamycin treatment (Supplementary Fig. 1L, M), supporting the conclusion that SRSF1 expression is dynamically regulated during ER stress at the level of protein synthesis.

Furthermore, low complexity sequences analysis from the OPP captured proteins list with the online-available tool: LCD-composer^31^, demonstrated a significant enrichment for lysine-rich domains. Lysine is a positively charged amino acid, usually found enriched in DNA/RNA-binding protein sequences. The gene ontology performed on the LCD proteins showed an enrichment for the RNA-binding term (Supplementary Fig. 1N). These findings indicate that ER stress activation profoundly modulates the translation of the splicing machinery and could thus remodel the transcriptome through splicing changes.

### Unfolded protein response reshapes alternative splicing landscape

**Fig. 2:**
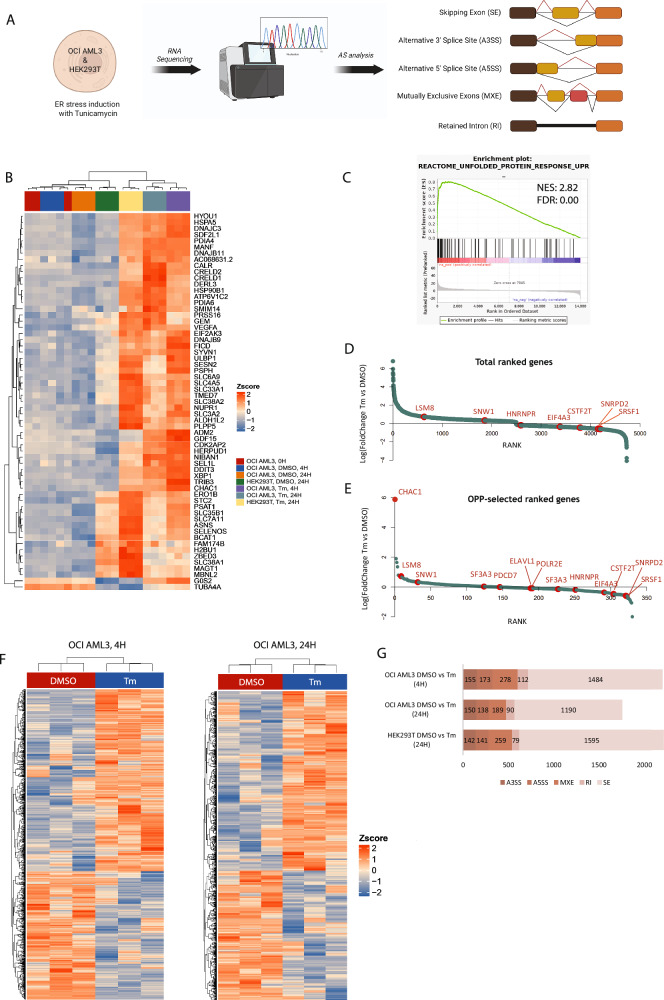
Unfolded protein response reshapes alternative splicing landscape. **A** Schematic depicts the splicing analysis strategy. OCI-AML3 and HEK293T cells are treated with tunicamycin at 1 μg/mL for 4H or 24H, and mRNA sequencing is performed. Splicing analysis with rMATS identifies splicing events. Splicing events are categorised as: skipped exon (SE), alternative 5’ splice site (A5SS), alternative 3’ splice site (A3SS), mutually exclusive exon (MXE), and retained intron (RI). *FDR* < *0.05 and Inclusion Level Difference*≥*|0.1*|*. Created in BioRender. Philippe, C. (2026)* https://BioRender.com/qdjfk6b. **B** Heatmap of differential gene expression in OCI-AML3 and HEK293T cells treated with tunicamycin at 1 μg/mL for 4H or 24H. two-sided moderated t-test (limma, voom). *adj.p* < 0.05*, logFC*≥ *|0.1* |. (**C**) Gene Set Enrichment Analysis of the differential gene expression for the Unfolded Protein Response. **D** Graph depicting the Log(Fold Change) of the ranked genes from the differential gene expression performed in OCI-AML3 treated with tunicamycin at 1 μg/mL for 4H. RNA-binding proteins annotated in red were captured by OPP pull-down in OCI-AML3 cells treated with 1 μg/mL tunicamycin for 4H. **E** Graph depicting the Log(Fold Change) of the ranked genes from the differential gene expression performed in OCI-AML3 treated with tunicamycin at 1 μg/mL for 4H. Only genes encoding proteins pulled down in the OPP analysis are represented here. RNA-binding proteins annotated in red were captured by OPP pull-down in OCI-AML3 cells treated with 1 μg/mL tunicamycin for 4H. **F** Heatmap of inclusion levels of differential splicing events in OCI-AML3 treated with DMSO (Red cluster) or 1 μg/mL tunicamycin (Blue cluster) for 4H (left Panel) or 24H (right panel). *FDR* < *0.05 and Inclusion Level Difference* ≥*|0.1*|. **G** Total number of alternative splicing events per category found in OCI-AML3 and HEK293T cells treated with 1 μg/mL tunicamycin for 4H or 24H. Splicing events are categorised as: skipped exon (SE), alternative 5’ splice site (A5SS), alternative 3’ splice site (A3SS), mutually exclusive exon (MXE), and retained intron (RI). *FDR* < *0.05 and Inclusion Level Difference*≥ *|0.1*|.

To investigate whether the UPR would reshape the splicing landscape, we performed RNA-sequencing and rMATS^32^ analyses on OCI-AML3 (a leukaemic cell line) and HEK293T cells (a non-cancerous cell line) 4 h and 24 h after induction of ER stress with tunicamycin (Fig. 2A). Unsupervised clustering led to the clustering of tunicamycin- induced samples distinctively from the cluster of DMSO treated samples (Supplementary Fig. 2A). Our gene expression analysis revealed the upregulation of classical UPR effectors among which are *HYOU1, HSPA5, EIFAK3, DNAJB9, DDIT3, XBP1* and *ERO1B* in both cell lines (Fig. 2B*,* C). We confirmed that the RNA binding proteins (RBPs) and splicing factors upregulated upon ER stress and captured by LC-MS/MS (Fig. 1D) are not regulated at the transcriptional level when compared to the overall global gene expression (Fig. 2D). Notably among the 350 proteins identified by LC-MS/MS (Supplementary Data 1), the gene expression of these splicing factors remains globally unchanged (Fig. 2E). This contrasts with other ER stress targets such as CHAC1, which is upregulated both at the RNA and protein levels (Figs. 1B*,* 2E). Differential splicing analysis using rMATS identified 2202, 1757, and 2216 alternative splicing events, respectively, in OCI-AML3, at 4 hours and 24 hours, and HEK293T cells, at 24 hours, with a false discovery rate (FDR) < 0.05 (Fig. 2F, Supplementary 2B*)*. We observed an increase in skipping exon (SE) events in both cell lines, at both time points (Fig. 2G*,* Supplementary 2C). Confirming the quality of the splicing analysis, we observed a remarkable non canonical splicing of XBP1 with an inclusion level of −0.665 (Supplementary Fig. 2D). Indeed, upon ER stress, the mRNA encoding the transcription factor XBP1 undergoes a non-canonical splicing by IRE1α in the cytoplasm that involves the excision of a 26-nucleotide intron, leading to a frame shift and the translation of a longer open reading frame^33^. REVIGO analysis of the alternative splicing events revealed that these transcripts are involved in pathways such as DNA repair, cell organisation, protein transport and RNA processing (Supplementary Fig. 2E).

### The Unfolded Protein Response promotes a robust ERi-splice signature across tissues

**Fig. 3:**
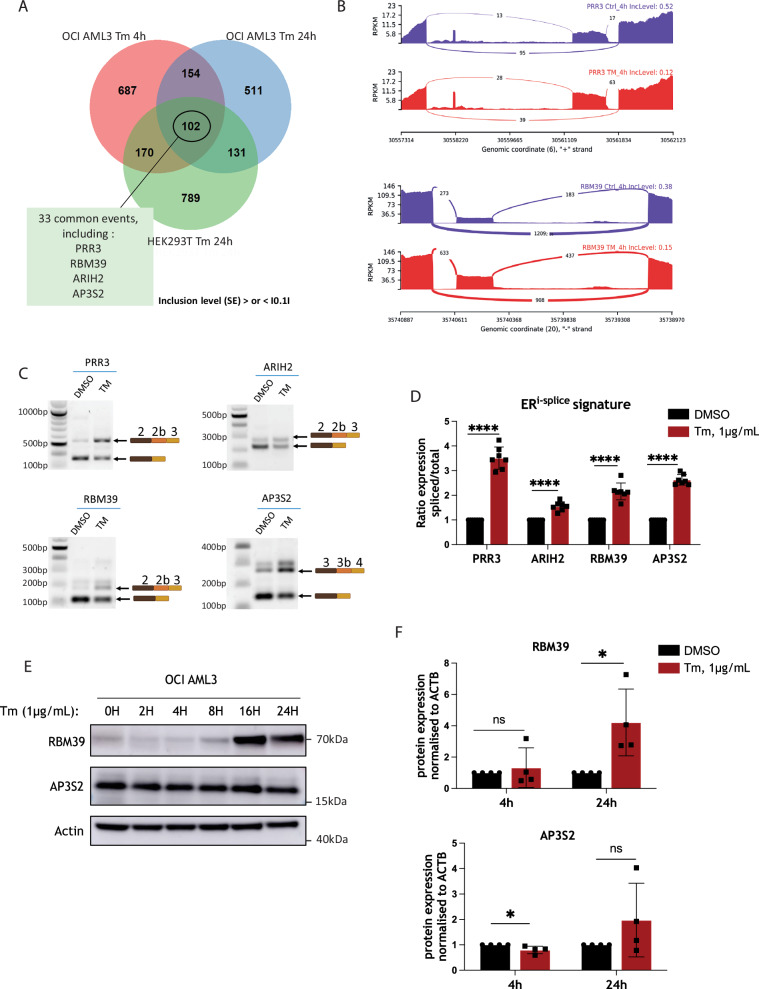
Unfolded protein response promotes a robust ER^i-splice^ signature across tissues. **A** Venn diagram depicting the overlap of genes undergoing differential skipped exon splicing in OCI-AML3 and HEK293T cells treated with 1 μg/mL tunicamycin for 4H or 24H. *FDR* < *0.05 and Inclusion Level Difference*≥*|0.1* | . From the common list of 102 transcripts, 33 present the same splicing coordinates (event). **B** Sashimi plots of two splicing events: *PRR3* (top panel) and *RBM39* (bottom panel), in OCI-AML3 treated with DMSO (in Purple) or with 1μg/mL tunicamycin (in Red) for 4H. **C**Representative gel of PCR products showing alternative splicing occurring in OCI-AML3 treated with tunicamycin at 1 μg/mL for 4H, for four of the splicing events: *PRR3, ARIH2, RBM39 and AP3S2*. **D**Quantitative PCR of four alternative splicing events (*PRR3, ARIH2, RBM39 and AP3S2)* occurring in OCI-AML3 treated with 1 μg/mL tunicamycin for 4H. Data are normalized respectively to total*PRR3, ARIH2, RBM39 and AP3S2* (n= 7 biological replicates). mean± S.D. two-sided unpaired t-test. **** *p* < 0.001. **E**Representative western blot of RBM39 and AP3S2 in OCI-AML3 cells treated with tunicamycin at 1 μg/mL for 0, 2, 4, 8, 16 or 24H. Actin B protein is used as a loading control. **F**Quantification of RBM39 (top panel) and AP3S2 (bottom panel) western blot in OCI-AML3 cells treated with tunicamycin at 1 μg/mL for 0, 2H or 4H. Results are normalized to Actin B. (*n* = 4 biological replicates). mean± S.D. two-sided unpaired t-test. * *p* < 0.05.

A Venn diagram of the transcripts undergoing differential exon skipping (FDR < 0.05 and Inclusion Level Difference > |0.1|) in OCI-AML3 and HEK293T cells treated with tunicamycin for 4 h or 24 h, revealed 102 alternatively spliced transcripts common to both cell lines at both time points. Out of the 102 genes with alternatively spliced variants, 33 transcripts exhibit identical coordinates (Fig. 3A, Supplementary Data 2). Within this subset, we specifically analysed four transcripts with alternative splicing levels spanning from 10% to 65%: *PRR3, RBM39, AP3S2* and *ARIH2* (Supplementary Fig. 3A). These four transcripts harboured an increase in exon inclusion (Fig. 3B, Supplementary Fig. 3B, C), while other transcripts, such as *EIF4A2* showed an increase in skipping exon exclusion (Supplementary Data 2). Polymerase Chain Reaction (PCR) analysis conducted using gel electrophoresis or QIAxcel, along with Reverse transcription quantitative-PCR (RT-qPCR) of the different transcript isoforms (with or without exon inclusion) validated our transcriptomic data (Fig. 3C, D, Supplementary Fig. 3D). These results confirm the induction of an ER induced splice/isoforms accumulation (ER^i-splice^) triggered upon ER stress induction, either with tunicamycin or thapsigargin (two well-established ER stress inducers), in **primary human cells** (Mesenchymal Stem cells: MSCs isolated from three different healthy donors) and **various cells lines** (Leukaemic: OCI-AML3, Human Embryonic Kidney:HEK293T, Hepato Carcinoma: HepG2, Cervical Cancer: HeLa, Prostate Cancer: PC3 and Breast Cancer: MCF7) (Fig. 3C, D, Supplementary Fig. 3E, F). Interestingly, these alternative splicing events can differently affect protein levels; for instance, RBM39 is upregulated upon ER stress, whereas the level of protein of AP3S2 remains unchanged (Fig. 3E, F).

### The ER^i-splice^ signature is driven by PERK

**Fig. 4:**
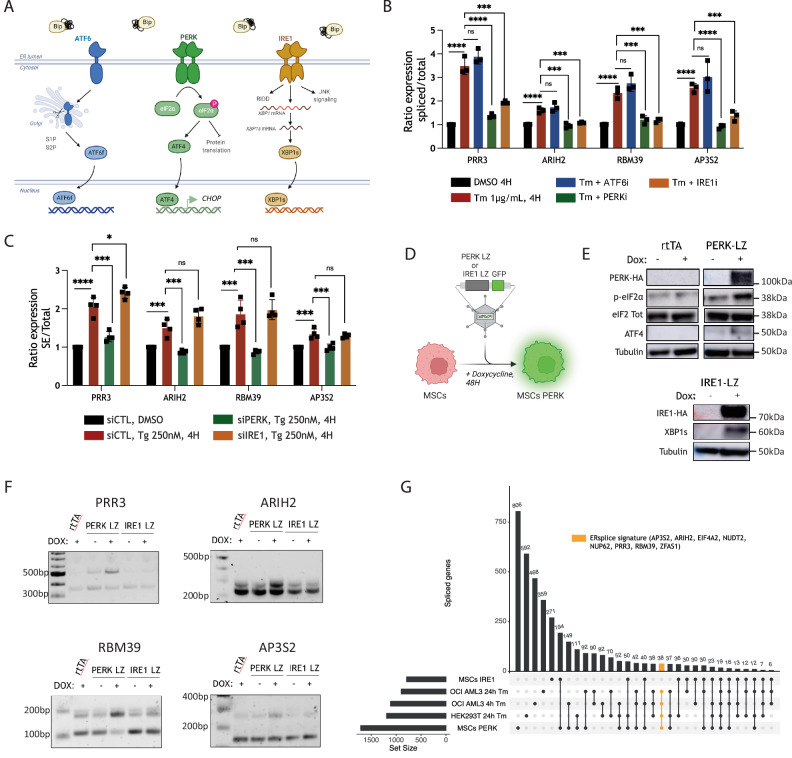
ER^i-splice^ signature is driven by PERK. **A** Schematic depicts the three pathways of the unfolded protein response: ATF6, PERK and IRE1, and their downstream signalling. *Created in BioRender. Philippe, C. (2026)* https://BioRender.com/ugrjezp. **B** Quantitative PCR of four alternative splicing events (*PRR3*, *ARIH2*, *RBM39* and *AP3S2*) occurring in OCI-AML3 treated with 1 μg/mL tunicamycin for 4H+/- PERK, ATF6 or IRE1 inhibitor, respectively, used at 300 nM, 10 μM, and 50 μM. Data are normalized respectively to total *PRR3, ARIH2, RBM39* and *AP3S2* (*n* = 3 biological replicates). mean± S.D. two-sided unpaired t.test. *p < 0.05, ***p < 0.005, ****p < 0.001. **C** Quantitative PCR of four alternative splicing events (*PRR3, ARIH2, RBM39*and *AP3S2)* occurring in HEK293T cells treated with 250 nM thapsigargin for 4H and transfected with either siRNA control (CTL), siRNA PERK, or siRNA IRE1 for 48H. Data are normalized respectively to total *PRR3*, *ARIH2*, *RBM39* and *AP3S2* (*n* = 4 biological replicates). mean± S.D. two-sided unpaired t.test. *p < 0.05, ***p < 0.005, ****p < 0.001. **D** Schematic depicts the gain-of-function strategy. Mesenchymal Stem Cells (MSCs) are transduced either with the PERK-LZ (LZ: Leucine Zipper) or the IRE1-LZ transgene, which allows the constitutive dimerization and activation of PERK and IRE1, respectively. Both lentivectors are doxycycline-inducible and contain an IRES-GFP cassette, allowing cells’ sorting. MSCs are transduced and treated with 1 μg/mL doxycycline for 48H prior to GFP sorting. *Created in BioRender. Philippe, C. (2026)* https://BioRender.com/dfn0uf1. **E** Representative western blot of PERK-HA, phospho-eIF2α, total-eIF2α and ATF4 (top panel) and IRE1-HA, XBP1s (bottom panel) in MSCs rtTA, PERK-LZ and IRE1-LZ cells treated with 1 μg/mL doxycycline for 48H. Tubulin protein is used as a loading control. (*n* = 3 biological replicates). **F** Representative gel of PCR products showing the alternative splicing events occurring in MSCs rtTA, PERK-LZ and IRE1-LZ cells treated with doxycycline at 1 μg/mL for 48H, for four of the splicing events: *PRR3*, *ARIH2*, *RBM39* and *AP3S2*. (*n* = 3 biological replicates). **G**UpSet plot depicting the overlap of genes undergoing differential splicing in MSCs PERK-LZ (MSCs PERK) and MSCs IRE1-LZ (MSCs IRE1) treated with 1 μg/mL doxycycline for 48H, and OCI-AML3 and HEK293T cells treated with 1 μg/mL tunicamycin for 4H or 24H. The 8 common splicing events (ER^i-splice^ signature) displaying the same coordinates are highlighted in orange. *FDR* < 0.05 and Inclusion Level Difference≥*|0.1*|.

To investigate which arm of ER stress is driving the ER^i-splice^ signature, we inhibited ATF6 with CEAPIN^34^, PERK with GSK2656157^35^ and IRE1 with MKC-3946^36^. Upon pharmacological inhibition of ATF6, no rescue of the alternative splicing signature is observed, whereas both PERK and IRE1 inhibitions rescue the alternative splicing of *PRR3, RBM39, AP3S2* and *ARIH2* (Fig. 4A, B). However as previously reported, our analysis confirmed that both inhibitors affect each other’s pathways^37,38^ (Supplementary Fig. 4A). Specifically, *XBP1s*, a target of IRE1, and *CHOP*, a target of PERK, are downregulated upon inhibition of PERK and IRE1, respectively (Supplementary Fig. 4A). To discriminate which of IRE1 or PERK is driving the ER^i-splice^ signature, we silenced *PERK* and *IRE1* with siRNAs. The results suggested that these alternative splicing events are driven by PERK (Fig. 4C, Supplemenary Fig. 4B). To confirm our findings, Leucine Zipper -PERK^39^ or -IRE1 plasmids were cloned into lentiviral vectors expressing GFP (Supplementary Fig. 4C). Upon co-transduction with rtTA (Reverse tetracycline responsive transcriptional activator) lentiviral vectors and doxycycline (DOX) treatment, PERK or IRE1 can be constitutively activated, and sorted by flow cytometry (Fig. 4D). We confirmed that PERK or IRE1 activation in primary human cells Mesenchymal Stem Cells (MSCs) faithfully recapitulated their respective UPR phenotypes (Fig. 4E). Then, the impact of PERK or IRE1 activation on splicing was assessed. Strikingly, only PERK activation led to exon inclusion in *PRR3, RBM39, AP3S2* and *ARIH2* (Fig. 4F).

We next asked whether the ER^i-splice^ phenotype could be recapitulated by other cellular stress conditions. To address this, we assessed the splicing of *PRR3*, *ARIH2*, *RBM39* and *AP3S2* in OCI-AML3 cells exposed to a range of stresses, including thermal stress (cold at 34 °C or heat at 42 °C), hypoxia (1% O₂), genotoxic stress (hydroxyurea, cisplatin, temozolomide), and metabolic stress (glutamine deprivation). Despite inducing cellular stress, none of these conditions fully reproduced the alternative splicing patterns observed upon ER stress induction (Supplementary Fig. 4D–F). These results indicate that the ER^i-splice^ signature is not a general feature of cellular stress responses but rather appears to be specific to ER stress.

To refine the ER^i-splice^ signature, RNA-sequencing and rMATS analyses were performed on MSCs-PERK or MSCs-IRE1 and integrated into the RNA-sequencing analysis performed with Tm at 4 h and 24 h on OCI-AML3 and HEK293T cells (Fig. 4G). Out of the 102 genes affected by splicing previously identified (Fig. 3A), 38 genes (Fig. 4G) are unique to PERK pharmacological or genetic activation. Among these 38 genes 8 share the same splicing coordinates (Supplementary Data 2), including *PRR3, RBM39, AP3S2, ARIH2* and *EIF4A2*.

### Nonsense-mediated decay inhibition leads to the accumulation of *PRR3* and *AP3S2* isoforms but not *RBM39* and *ARIH2* isoforms

**Fig. 5:**
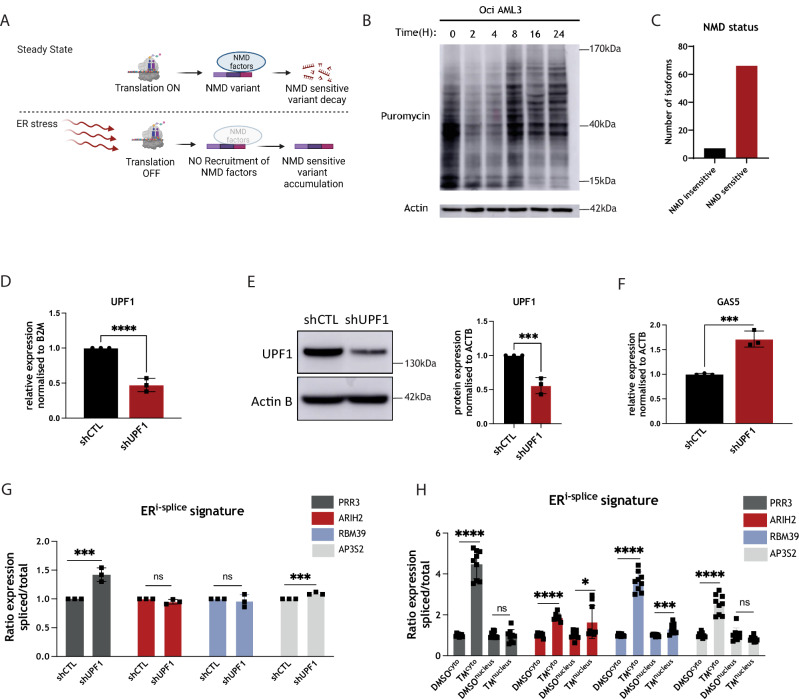
Non-sense-mediated decay inhibition leads to accumulation of *PRR3* and *AP3S2* isoforms but not *RBM39* and *ARIH2* isoforms. **A** Schematic depicts the interplay between protein synthesis and non-sense-medicated decay (NMD) upon endoplasmic reticulum stress. During the first round of translation, the ribosome can detect a premature stop codon (PTC) and trigger NMD activation. During ER stress, translation is inhibited, thereby impairing PTC recognition and NMD recruitment*. Created in BioRender. Philippe, C. (2026)* https://BioRender.com/kt4fm94. **B** Sunset Assay showing de novo peptides tagged with 2 μM puromycin for 30 min following 0, 2, 4, 8, 16 and 24H tunicamycin treatment, at 1 μg/mL, in OCI-AML3 cells. Actin protein is used as a loading control. (*n* = 3 biological replicates). **C** Number of NMD insensitive and sensitive isoforms predicted with the IsoformSwitchAnalyzeR package, in OCI-AML3 cells treated with 1 μg/mL tunicamycin for 4H. **D** Quantitative PCR analysis of *UPF1* mRNA levels in OCI-AML3 cells transduced with a shControl (shCTL) or a shUPF1. Data are normalized to Beta-2-Microglobulin (*n* = 3 biological replicates). mean± S.D. two-sided unpaired t.test. ****p < 0.001. **E** Representative western blot of UPF1 (left panel) and its quantification (right panel) in OCI-AML3 cells transduced with a shControl (shCTL) or a shUPF1. Actin B protein is used as a loading control. (*n* = 3 biological replicates). mean± S.D. two-sided unpaired t.test. ***p < 0.005. **F** Quantitative PCR analysis of the NMD-sensitive *GAS5* isoform in OCI-AML3 cells transduced with a shControl (shCTL) or a shUPF1. Data here are normalized to Actin B (*n* = 3 biological replicates). mean± S.D. two-sided unpaired t.test. ***p < 0.005. **G** Quantitative PCR of four alternative splicing events (PRR3, ARIH2, RBM39 and *AP3S2)* occurring in OCI-AML3 transduced with a shControl (shCTL) or a shUPF1. Data are normalized respectively to total *PRR3, ARIH2, RBM39* and *AP3S2* (*n* = 3 biological replicates). mean± S.D. two-sided unpaired t-test. ***p < 0.005. **H** Quantitative PCR of four alternative splicing events (*PRR3*, *ARIH2*, *RBM39* and *AP3S2*) occurring in cytoplasmic or nuclear fractions of OCI-AML3 treated with 1 μg/mL tunicamycin for 4H. Data are normalized respectively to total *PRR3*, *ARIH2*, *RBM39* and *AP3S2* (*n* = 3 biological replicates, with 3 technical replicates). mean± S.D. two-sided unpaired t.test. *p < 0.05, ***p < 0.005, ****p < 0.001.

PERK governs global protein translation rate through the phosphorylation of eIF2α^40^. Tightly coupled to the process of protein translation is an essential mechanism of mRNA quality control called Nonsense-mediated mRNA decay (NMD) that recognizes and degrades mRNA molecules containing premature termination codons (PTCs)^41^ (Fig. 5A). Its primary function is to prevent the translation of potentially aberrant proteins that may arise due to errors in transcription or splicing^41^, moreover it is known that PERK is required to inhibit NMD^42^. Therefore, it is legitimate to wonder whether the ER^i-splice^ signature observed upon PERK activation is the consequence of an indirect inhibition of NMD through global protein translation attenuation. SUNSET assay performed at different time points on OCI-AML3 treated with tunicamycin confirmed a global protein translation attenuation up to 4 h post-treatment (Fig. 5B). Curation of the RNA-sequencing data generated upon ER stress activation, revealed an increase of mRNA molecules, with shorter open reading frames that reflects an expected translation reprogramming upon ER stress (e.g. *ATF4*) (Supplementary Fig. 5A) and with NMD-sensitive mRNAs (Fig. 5C). Our various endeavours to pharmacologically disentangle protein translation from NMD using protein translation inhibitors (cycloheximide or omacetaxine) or a potent NMD inhibitor (SMG1i) systematically triggered the activation of the PERK pathway (Supplementary Fig. 5B*)*. Consequently, pharmacological approaches rendered the discrimination of PERK- or NMD-induced splicing isoforms accumulation impossible.

To overcome this limitation and shed light on splicing regulation upon ER stress activation, we employed a genetic approach. The identification of a PTC or other relevant features prompts the phosphorylation of UPF1 (RENT1), a pivotal NMD factor^43^ also captured by our OPP-LC-MS/MS assay. Thus, we silenced *UPF1* with a lentiviral approach. OCI-AML3 cells were transduced with a shRNA control (shCTL) or shRNA against *UPF1* (shUPF1) and sorted based on the expression of the reporter gene GFP (Supplementary Fig. 5C). Western blot and RT-qPCR analyses showed 50% loss of expression of UPF1 at the mRNA and protein levels (Fig. 5D, E). This loss was sufficient to trigger the accumulation of known NMD-sensitive isoforms such as *GAS5*^44^ (Fig. 5F) without triggering a UPR response (Supplementary Fig. 5D). Then RT-qPCR analyses of *PRR3, RBM39, AP3S2* and *ARIH2*, in OCI-AML3 silenced for *UPF1*, demonstrated that *PRR3* and *AP3S2* are likely to be NMD-sensitive isoforms accumulating upon NMD inhibition, although the effect on *AP3S2* is subtle (Fig. 5G). Next, we used an orthogonal approach to discriminate passive (due to NMD inhibition) versus active (due to splicing machinery) accumulation of isoforms upon ER stress. NMD is coupled to protein translation and occurs in the cytoplasm, while splicing occurs in the nucleus; therefore, we isolated RNA from nuclear and cytoplasmic fractions of OCI-AML3 cells treated with tunicamycin or DMSO. *XBP1s (spliced in the cytoplasm by IRE1)* and *ARIH2 pre-mRNA* were used as positive controls of cytoplasmic and nuclear extracts, respectively (Supplementary Fig. 5E). Accumulation of NMD-sensitive isoforms should be observed only in the cytoplasm, while isoforms actively spliced in the nucleus and shuttled to the cytoplasm should be observed in both fractions. Hence, we confirmed that *PRR3* and *AP3S2* are likely to be NMD-sensitive isoforms while *RBM39* and *ARIH2* are actively spliced in the nucleus upon ER stress activation (Fig. 5H).

### PERK-driven alternative splicing is mediated through the splicing factor kinase CLK

**Fig. 6:**
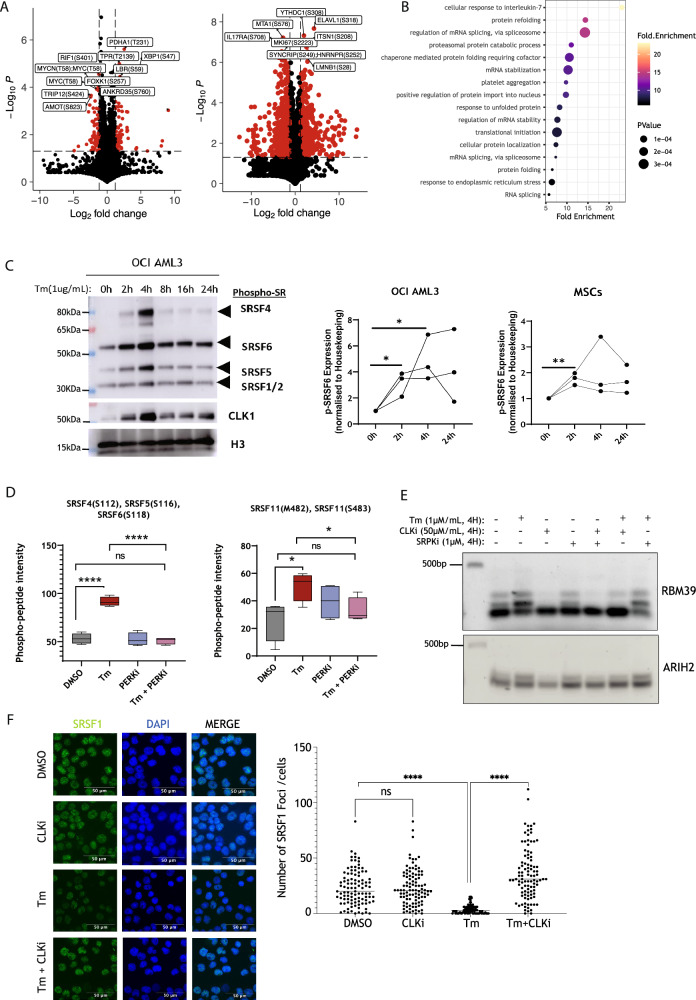
PERK-driven active alternative splicing is mediated through the Splicing Factor Kinase CLK. **A** Volcano plots showing the differential phosphorylation of peptides in OCI-AML3 cells treated with 1 μg/mL tunicamycin for 4H (left panel) or 24H (right panel). two-sided unpaired t.test. **B** Gene ontology enrichment analysis performed on differentially phosphorylated peptides from OCI-AML3 cells treated with 1 μg/mL tunicamycin for 24H. one-tailed Fisher Exact Test. **C** Representative western blot of phospho-SR and CLK1 proteins in OCI-AML3 cells treated with 1 μg/mL tunicamycin for 0, 2, 4, 8, 16, and 24H (left panel). Histone H3 protein is used as a loading control. Quantification of phospho-SRSF6 protein expression, in OCI-AML3 and MSCs cells treated with tunicamycin at 1 μg/mL, is displayed (right panel). Each dot-connected line represents an individual western blot quantification. Data are normalized to the respective housekeeping gene (*n* = 3 biological replicates). mean± S.D. two-sided paired t-test. * *p* < 0.05, **p < 0.01. **D** Normalised intensity for the phosphopeptides SRSF4(S112) or SRSF5(S116) or SRSF6(S118), and SRSF11(M482) or SRSF11(S483), in OCI-AML3 cells treated with 1 μg/mL tunicamycin +/300 nM PERK inhibitor for 2H (*n* = 4 biological replicates). Box plots show the median, 25th–75th percentiles (IQR), and whiskers extending to the minimum and maximum observed values. two-sided unpaired t.test. *p < 0.05, ****p < 0.001. **E** Representative gel PCR showing the alternative splicing events for two of the splicing events (*RBM39 and ARIH2*) occurring in OCI-AML3 treated with tunicamycin at 1 μg/mL for 4H, +/– CLK or SRPK inhibitors, used respectively at 50 μM or 1 μM in co-treatment. (*n* = 3 biological replicates). **F** Representative pictures of SRSF1 immunofluorescence staining (left panel) performed in OCI-AML3 cells treated +/− 1 μg/mL tunicamycin +/− 50 μM CLK inhibitor for 4H. Scale bar = 50 µm. Quantification of nuclear SRSF1 Foci (right panel). (*n* = 3 biological replicates, with 100 nuclei counted for each condition). mean± S.D. two-sided unpaired t.test. ****p < 0.001.

PERK kinase activity prompted us to perform phosphoproteomic LC-MS/MS analysis on protein extracts obtained from OCI-AML3 cells treated with tunicamycin. Phosphoproteomics revealed a global decrease in peptide phosphorylation 24 h after tunicamycin treatment (Fig. 6A and Supplementary Data 3). Notably, the set of peptides with increased phosphorylation after treatment showed an enrichment of proteins involved in mRNA splicing and protein folding in a gene ontology analysis (Fig. 6B, Supplementary Data 3). Among splicing factors, many are serine/arginine-rich (SR) proteins regulated by phosphorylation^45^. To investigate whether the splicing factor’s phosphorylation status was altered upon ER stress induction, we performed western blots on OCI-AML3 and MSCs treated with tunicamycin or thapsigargin. The blots, probed with a pan phospho-SR antibody, unveiled a notable increase in the phosphorylation of SR proteins at 4 h in both models (Fig. 6C, Supplementary Fig. 6A) with modulations seen as early as 2 h post-treatment concomitant with the expression of CLK1 (Fig. 6C, Supplementary Fig. 6B). To confirm the contribution of the PERK pathway to these phosphorylation events, OCI-AML3 were treated for 2 h with tunicamycin, in presence or absence of PERK inhibitor (GSK2656157), prior to undergoing phosphoproteomic LC-MS/MS analysis. This approach demonstrated a PERK-dependent phosphorylation of SR proteins such as SRSF4 and SRSF11 within 2 h of ER stress induction (Fig. 6D and Supplementary Data 4). For completeness, acetyl- and methyl- peptides were investigated by proteomic LC-MS/MS in OCI-AML3 treated for 4 h with tunicamycin, in the presence or absence of PERK inhibitor (Supplementary Fig. 6C, Supplementary Data 5). Our analysis revealed a specific acetylation of the Ribosomal Protein S7 (RS7) and methylation of the G Protein Subunit Alpha I1 (GNAI1), while no SR peptides were detected (Supplementary Fig. 6D).

SR proteins are the target of two well-established kinase families, the Serine-Arginine Protein Kinase (SRPK) and the CDC-like Kinase (CLK)^46^ (Supplementary Fig. 6E). Thus, we investigated whether SRPK or CLK inhibition could rescue the splicing phenotype observed upon ER stress activation. Strikingly, CLK inhibition rescues the splicing of *RBM39* and *ARIH2* (Fig. 6E, Supplementary Fig. 6F), without affecting *PRR3* and *AP3S2* splicing (Supplementary Fig. 6G), while SRPK inhibition had no effect on these different events. These findings confirm that *RBM39* and *ARIH2* are actively spliced in the nucleus upon ER stress activation (Figs. 5H*,* 6E).

As CLK1 is a major regulator of splicing factors’ activity, we tracked by immunofluorescence the endogenous SRSF1’s behaviour upon ER stress activation and CLK1 inhibition. Indeed, Jacky Chi Ki Ngo et al.^46^, showed that CLK1 causes release/diffusion of SRSF1 from nuclear speckles by phosphorylating the C-terminal part of its arginine-serine (RS)-rich domain, allowing SRSF1 to carry out its functions. Therefore, we performed an immunofluorescence staining of SRSF1 upon ER stress activation in the presence or absence of CLK1 inhibitor TG003. Here we show for the first time that ER stress activation releases SRSF1 from nuclear speckles and that this phenotype can be prevented by inhibition of CLK1 kinase activity with TG003 (Fig. 6F).

### The inhibition of the axis CLK1/SRSF1 silencing rescues ER stress-induced splicing and synergises with tunicamycin to enhance cell death

**Fig. 7:**
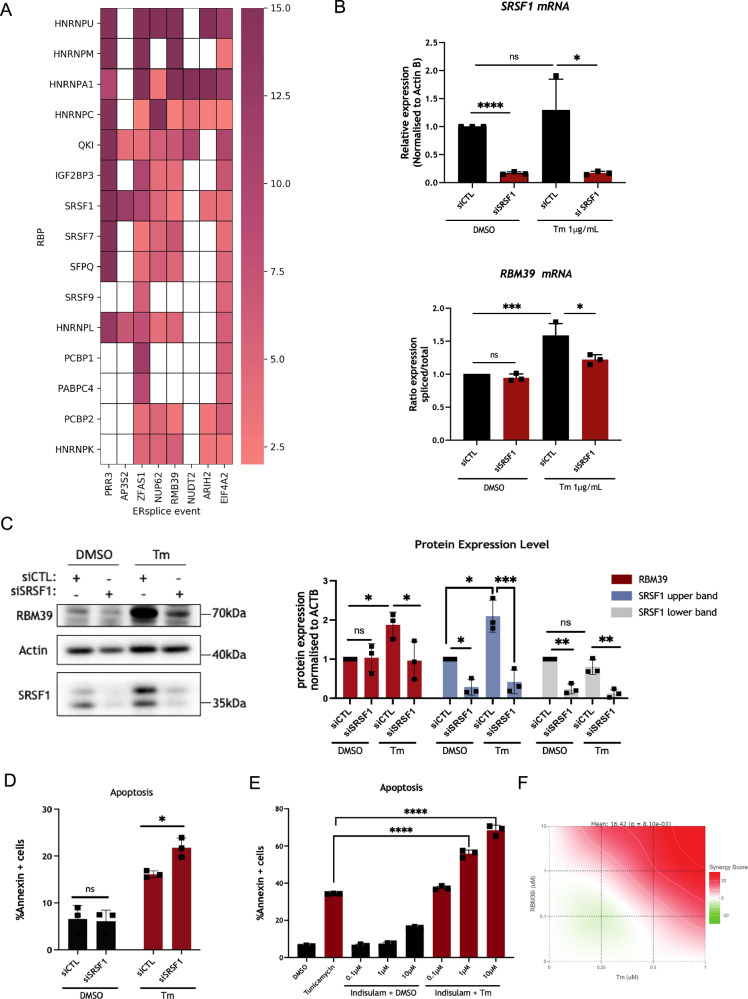
SRSF1 ER stress-mediated upregulation controls RBM39 expression. **A** Top RNA Binding Proteins for each of the 8 events filtered for RNA Binding Proteins that have ENCODE eCLIP data available and supporting eCLIP peaks in the ER^i-splice^ events. **B** Quantitative PCR of *SRSF1* (top panel) or *RBM39* splicing event (bottom panel) in OCI-AML3 transfected with a siControl (siCTL) or a siSRSF1 for 48H and treated +/− 1 μg/mL tunicamycin for 4H. Data are normalized respectively to *Actin B* (for *SRSF1*) and to total *RBM39* (for *RBM39* splicing event) (*n* = 3 biological replicates). mean± S.D. two-sided unpaired t.test. *p < 0.05, ***p < 0.005, ****p < 0.001. **C** Representative western blot of RBM39 and SRSF1 (left panel) in OCI-AML3 cells transfected with a siControl (siCTL) or a siSRSF1 for 48H and treated with tunicamycin at 1 μg/mL for 4H. Actin B protein is used as a loading control. Quantification of RBM39 and SRSF1 (right panel) western blot. Results are normalized to Actin B. (*n* = 3 biological replicates). mean± S.D. two-sided paired t.test. * *p* < 0.05, ** *p* < 0.01, *** *p* < 0.005. **D** Apoptosis assessed by Annexin V staining in OCI-AML3 transfected with a siControl (siCTL) or a siSRSF1 for 48H and treated with or without tunicamycin at 1 μg/mL for 24H (*n* = 3 biological replicates). mean± S.D. two-sided unpaired t.test. * *p* < 0.05. **E** Apoptosis assessed by Annexin V staining in OCI-AML3 treated with or without tunicamycin at 1 μg/mL, and with or without indisulam at 0.1 μM, 1 μM or 10 μM for 48H. (*n* = 3 biological replicates). mean± S.D. two-sided unpaired t.test. ****p < 0.01. **F** Synergy score between tunicamycin and indisulam (RBM39i) by using the Bliss model (ZIP Score > 10 indicates synergism) in OCI-AML3 treated with different combination doses of tunicamycin at 0.25 μg/mL, 0.5 μg/mL or 1 μg/mL, and indisulam at 0.1 μM, 1 μM or 10 μM for 48H. (*n* = 4 biological replicates).

Using the splicing event coordinates and performing FIMO analysis, we identified significant enrichment of RBP binding sites, with SRSF1 emerging as the top-ranked binding factor (Supplementary Fig. 7A). As an orthogonal validation, we applied in silico analysis with SpliceLearn™, an AI/ML-based pipeline designed to predict RBP binding sites^47^. This approach confirmed SRSF1, along with HNRNPU, QKI, and HNRNPC, as the most probable RBPs binding to the flanking exons of the eight transcripts in our ER^i-splice^ signature. SpliceLearn integrates available ENCODE eCLIP data and supporting eCLIP peaks to predict RBP binding (Fig. 7A).

SRSF1 is identified by OPP-LC-MS/MS (Fig. 1D–F), released from nuclear speckles upon ER stress (Fig. 6F) and predicted as the top RBP by FIMO and SpliceLearn™ run on the 33 common splicing events (Fig. 7A*,* & Supplementary Fig. 7A). We therefore silenced *SRSF1* with siRNA and assessed its impact on ER stress-induced splicing and cell death. We achieved over 90% of silencing for *SRSF1* and showed that *SRSF1* silencing prevents *RBM39* splicing and protein expression upon ER stress activation (Fig. 7B, C). Furthermore, while *SRSF1* silencing alone barely affects cell death, its combination with the ER stress inducer tunicamycin induces a dramatic increase in cell death (Fig. 7D). Consistent with this observation, inhibition of CLK1, the upstream kinase of SRSF1, also reproduces this phenotype (Supplementary Fig. 7B).

Finally, the heightened splicing and protein expression of *RBM39* controlled by SRSF1 upon ER stress activation suggest a potential biological role for this protein. In order to delve into its biological significance, we used the inhibitor Indisulam, targeting RBM39, (Supplementary Fig. 7C) and performed a synergistic experiment with the ER stress inducer, tunicamycin. Our results unequivocally reveal that Indisulam and tunicamycin synergistically amplify ER stress-induced cell death (Fig. 7E*, F*).

## Discussion

The intricate relationship between the Unfolded Protein Response (UPR) and alternative splicing, as revealed in our study, underscores a previously underappreciated dimension of cellular stress responses. The discovery that PERK orchestrates a conserved program of alternative splicing, akin to the Regulated IRE1α-dependent Decay (RIDD)^6,7^, spanning from healthy to malignant cells, marks a significant advance in our understanding of cellular adaptation by mechanisms to ER stress (Supplementary Fig. 7D).

The observation that proteins of the splicing machinery are translated upon the UPR, particularly under conditions of ER stress, is remarkable. The enhanced synthesis of RNA-binding proteins and splicing factors by the translational machinery, independent of transcriptional changes, suggests a rapid, post-translational mechanism for enhancing the cell’s capacity to modify its transcriptome in response to stress.

SF3A3 and SRSF1, which are both translated upon ER stress and captured by our OPP-LC-MS/MS analyses, are two splicing factors playing key roles during tumourigenesis. The alternative splicing factor oncoprotein SRSF1 is upregulated in many cancers, including in breast cancers, where its overexpression alone is sufficient to trigger a cancerous phenotype through splicing of pro-apoptotic transcripts and partially through cooperation with MYC^48^. Therefore, it is noteworthy to observe SRSF1 translation and hyper-phosphorylation upon ER stress^49–53^. SF3A3 is a key component of the spliceosome that undergoes translation-based regulation upon MYC activation, impacting the splicing of mRNAs associated with mitochondrial regulation. Dysregulation of SF3A3 translation leads to metabolic changes and stem-like properties, enhancing MYC-driven tumorigenic potential and serving as a marker with significant value for aggressive breast cancers^18^. Therefore, our data suggest that ER stress could represent a means for cancerous cells to hijack key components of the splicing machinery and dictate their spliceosome landscape.

The identification of Serine and Arginine-rich splicing factors (SRSF) and RNA-binding proteins de novo synthesized and post-translationally modified upon ER stress indicates a direct link between UPR activation and the modulation of the splicing machinery. Our analysis of alternative splicing changes in seven cell lines sourced from different tissues reveals a broad reorganization of the splicing landscape, with an increased incidence of exon skipping events. This reorganization is not only a response to stress but also a reflection of the cell’s adaptive strategies, reshaping gene expression profiles to cope with altered environmental conditions.

The low complexity sequences analysis from the OPP captured proteins list demonstrated a significant enrichment for lysine and glutamic acid-rich domains. Lysine is of peculiar interest as positively charged amino acids are usually found enriched in DNA/RNA-binding protein sequences. On the contrary, glutamic acid is a negatively charged amino acid. For the stress response proteins, an enrichment in acidic glutamic acid could potentially ensure their exclusion from the cytoplasmic RNA granules generated upon stress, such as the stress granules.

The identification of a specific ER splice signature (ER^i-splice^) across different cell types, including primary human cells, further supports the notion of a universal UPR-driven mechanism governing alternative splicing under stress conditions.

The role of PERK in driving the ER^i-splice^ signature is particularly noteworthy and underscores its broader impact on gene expression regulation. The interplay between PERK-driven splicing and Nonsense-mediated Decay (NMD) highlights the complexity of cellular responses to ER stress. For instance, the core factor of NMD, RENT1/UPF1, is translated upon ER stress along with the core catalytic unit of CCR4-NOT, CNOT7, responsible for mRNA deadenylase activity. The challenge to uncouple ER stress from NMD, for example, with omacetaxine, cycloheximide or the inhibitor SMG1 (Supplementary Fig. 5B), suggests a complex interplay between these key quality control mechanisms and pleads for further investigation. Indeed, the accumulation of specific splice isoforms upon NMD inhibition suggests a dual mechanism by which PERK influences gene expression: directly through the induction of alternative splicing events and indirectly by modulating NMD.

The phosphorylation of splicing factors, as mediated by PERK, opens new avenues for understanding the post-translational regulation of splicing under stress. The specific role of the Splicing Factor Kinase, CLK, in mediating active splicing changes during ER stress, as opposed to SRPK, underscores the specificity of cellular responses to different stress signals.

Nuclear speckles are dynamic subnuclear structures that regulate the storage, modification, and availability of splicing factors, and their organisation is highly sensitive to cellular stress. SR protein behaviour within speckles is stress-specific. For instance, oxidative stress and heat shock reduce SR protein phosphorylation, whereas hypoxia increases it^54^. SRSF5 relocalises from speckles under hypoxia or arsenite but not upon MG132 treatment^55^ and SRSF6 downregulation under hypoxia leads to speckle dispersal^56^, highlighting the diversity and the specificity of stress-dependent responses.

In this context, CLK1 plays a central role by phosphorylating the arginine-serine domain of SR proteins, thereby promoting their release from nuclear speckles and enabling splicing activity^46^. Consistent with this mechanism, we show that ER stress induces redistribution of SRSF1 from nuclear speckles, which is prevented by CLK1 inhibition using TG003 (Fig. 6F), supporting a CLK1-dependent regulation of splicing factor dynamics.

As nuclear speckles are affected by different stresses, we tested a range of cellular stresses, including thermal stress, hypoxia, genotoxic stress, and metabolic stress. None of these conditions fully reproduced the alternative splicing events observed upon ER stress (Supplementary Fig. 4D–F), indicating that the ER^i-splice^ signature is not a generic consequence of cellular stress but is specific to ER stress.

Finally, an important distinction may lie in the kinetics of stress exposure. While many reported effects on splicing changes occur after prolonged stress (typically 24 h), our observations are made at early time points (4 h), suggesting that ER stress triggers a rapid and specific reprogramming of splicing factor dynamics and activity.

SRSF1 is a core member of the SR protein family, essential for both constitutive and alternative splicing. It is frequently overexpressed in human cancers, where it promotes oncogenic transformation and splicing programs^57–59^. Our work identifies ER stress as a potential mechanism driving SRSF1 upregulation and its associated oncogenic programs^17^.

Remarkably, the majority of transcripts identified by our ER^i-splice^ signature appear to be intricately involved in the critical processes of protein quality control and splicing biology. This observation emphasizes the pivotal role that ER stress-driven splicing plays in the stress response mechanism, extending its influence from protein quality control to the intricate details of transcription regulation through canonical splicing and advocating for further investigation of the role of the different isoforms generated.

For instance, ARIH2 is an ubiquitin E3 ligase that may play an important role in protein quality control in the ER^60^ while eIF4A2 enables positive regulation of miRNA-mediated inhibition of translation^61^. The splicing of *RBM39* is of particular interest as RBM39 is an RNA-binding protein that was found to be crucial for maintaining RNA splicing and AML survival. Its genetic or pharmacologic targeting altered RNA splicing, which is especially lethal to AML cells with spliceosomal mutations^62^. The functional implications of these findings are further underscored by our observation that RBM39 inhibition synergizes with ER stress inducers to amplify cell death. As splicing factor and RNA-binding protein mutations affect protein homoeostasis, our findings suggest a potential therapeutic avenue in cancer treatment, where ER stress-inducing drugs could enhance the efficacy of splicing factor modulators.

Future work will be required to resolve isoform-specific consequences of ER stress-induced splicing changes, as short-read RNA-seq does not allow unambiguous assignment of full-length transcript variants. Targeted functional analyses will therefore be essential to define the protein-level impact of individual splicing events. In addition, the molecular mechanism linking ER stress to CLK1 activation remains to be fully elucidated. Addressing these questions will be important to further refine the regulatory framework connecting ER stress signalling to splicing control.

In conclusion, our study sheds light on a novel aspect of UPR-mediated cellular adaptation and highlights the broader implications of alternative splicing in cellular stress responses. These findings pave the way for future research into targeted therapies that could exploit the vulnerabilities of cancer cells in managing ER stress and alternative splicing^63^.

## Methods

### Cells and culture conditions

OCIAML3, PC3 cells were grown in RPMI (Thermo Fisher, #21875-034), and HEK293T, HepG2, HeLa, MCF7 cells were grown in DMEM (Thermo Fisher, #41966-029). RPMI and DMEM media were supplemented with 10% FBS (Thermo Fisher) and 1% penicillin-streptomycin (Thermo Fisher). Cells were incubated at 37 °C with 5% CO_2_. Bone marrow samples from healthy donors (PMID: 36156210) were obtained after signed informed consent. Mononuclear cells (MNCs) were isolated by using the Ficoll-Paque density centrifugation method (GE Healthcare Life Sciences) and red blood cell lysis (BioLegend, #420301). Following CD34+ cells selection by magnet (StemCell Tech, #17898), the CD34- fraction was kept and seeded in mesenchymal stem cell (MSC) medium (Gibco™ MEMα, nucleosides, GlutaMAX™ Supplement, #32571093, containing 10% Gibco MSC-qualified, #12662029, 1% penicillin-streptomycin). MSCs were incubated at 37 °C with 5% CO_2_, and isolated by plastic adherence. Medium was replaced 24H after plating and later 3-4 days until confluence was reached. At 80%-90% confluence, the cells were incubated with 0.025% trypsin and 0.01% EDTA (Thermo Fisher, #AM9261) and subsequently passaged. Cells were frozen at passage 2 and used up to passage 7. Following drugs were used for treatments: Tunicamycin (Merck, #T7765), Thapsigargin, (Merck, #586005), Cycloheximide (Merck, #C4859), CLKi (TG003, Thermo Fisher, #J64690.LB0), SRPKi (SPHINX31, Cambridge Bioscience, #21582), PERKi (GSK 2656157, Generon, #A13577-10), IRE1i (MKC-3946, Insight Biotechnology, #HY-19710), ATF6i (Ceapin A7, Merck, #SML2330), Doxycycline (Merck, #D3347), Omacetaxine (Santa Cruz, #26833-87-4), SMGi (hSMG-1 inhibitor 11e, Cambridge Bioscience, #HY-124760), Puromycin (Merck, #P8833).

### Vectors construction

pTRIP-TRE-PLZ and pTRIP-TRE-ILZ gain-of-function constructs and rtTA lentivectors were kindly gifted by Dr Christian Touriol (Cancer Research Center of Toulouse, Inserm, Université Toulouse III). IRES-EGFP cassette from pIRES2-EGFP (Addgene, #6029-1) was cloned as a eukaryotic selectable marker in the original vectors by using In-Fusion® HD Cloning (Takara, #639298) following the manufacturer’s instructions. Primers used for cloning are available in Data S6. PERK-LZ and IRE1-LZ vectors were linearized using the restriction enzyme KpnI (NEB, # R3142S). The IRES-EGFP sequence was amplified with CloneAmp HiFi PCR Premix Kit (Takara, #639298) by using the iresEGFP-pTRIP primers, and inserted into the linearized vector by homologous recombination to generate pTRIP-TRE-PLZ-iresEGFP (PERK-LZ), and pTRIP-TRE-ILZ-iresEGFP (IRE1-LZ). Plasmid size was checked by restriction enzyme digestion and gel electrophoresis with 0.5% (w/v) agarose gel in Tris-Borate-EDTA (TBE) buffer and sequences were confirmed by DNA sequencing (Eurofins). Lentiviral vectors shControl (CCTAAGGTTAAGTCGCCCTCG) and shUPF1 (CCTGCGTGGTTTACTGTAATA) were purchased from Vectorbuilder.

### Vectors amplification et virus production

All vectors were amplified by transformation of NEB 5-alpha competent E. Coli (NEB, #C2987) and overnight growth in LB supplemented with ampicillin or kanamycin at 37 °C under agitation. Plasmids were purified using Invitrogen™ PureLink™ HiPure Plasmid Maxiprep Kit (Thermofisher, #K210007). Viruses were produced by transfecting HEK293T cells with each vector along with packaging components pCMVdR8.74 and pMD.G2 using the CaCl2 method. Medium containing the virus was collected at 24 and 48 hours, centrifuged at 500 x *g* for 8 min and filtered through a 0.22 µm filter to eliminate cell debris. The viruses were then concentrated using an ultracentrifuge at 22,000 rpm for 2 hours at +4 °C and resuspended in 200 µl of the appropriate medium (RPMI or MSC medium). Aliquots of concentrated viral particles were kept at −80 °C for long-term storage.

### Cells transduction and selection

To generate stable transduced cell lines, 3.5 million cells were harvested and resuspended in 12 ml of the virus-containing medium at a multiplicity of infection (MOI) of 10. For the gain-of-function vectors, MSCs were co-transduced with rtTA and PERK-LZ or IRE1-LZ gain-of-function plasmids at 1:1 ratios (rtTA:PERK-LZ or rtTA:IRE1-LZ). MSCs transduced with rtTA alone are used as controls for experiments. Resuspended cells are then seeded in 150 mm flasks at high confluence. After ~12 hours, the cells were washed, resuspended in fresh medium and expanded. For cells in suspension, virus particles were added directly to the cells (1million/mL) at a MOI of 10 and incubated at 37 °C, 5% CO_2_ overnight. Cells were then washed with fresh medium and expanded for 4 days prior cell sorting. Selection of transduced cells, either shRNA or gain-of-function models, was based on EGFP cell sorting with the FACSAria™ Fusion (BD Biosciences).

### Cells transfection

300,000 cells were seeded in a 6-well plate 24H prior to transfection. Cells were then transfected with either siCTL (Dharmacon, #D-001210-02-05), siIRE1 (Santa Cruz, #sc-40705), siPERK (Dharmacon, #L-004883-00-0005), or siSRSF1 (Dharmacon, #L-018672-01-0005) using Lipofectamine RNAiMAX (Thermo Fisher, #13778075) according to the manufacturer’s instructions. Medium was then changed 24H following transfection, and cells were treated 24H later with either DMSO, tunicamycin (1 µg/mL) or thapsigargin (250 nM) for 4H.

### Cellular fractionation

5 million OCI-AML3 cells were collected and washed with ice-cold PBS. Cell pellet was then gently resuspended in 200 µL of saponin extract buffer (50 mM HEPES at pH 7.5, 0.05% [w/v] saponin, 50 nM 2-mercaptoethanol, 2 mM MgCl_2_, 0.025% NP40, 1 mM DTT) supplemented with protease inhibitor cocktail (Thermo Fisher, #A32961). Lysates are incubated on ice for 5 min, and then centrifuged at 4 °C for 3 min at 300 g. The cytoplasmic fraction was collected after centrifugation and represents 180 µL of the supernatant. Pellets contain the nuclei. In order to remove the residual nuclei in the cytoplasmic fraction, this fraction was spun again at max speed for 3 min and then stored at −80 °C. In parallel, nuclei pellets are washed in 1 mL of saponin extract buffer and left on ice for 5 min. Nuclei were then centrifuged at 300 g for 3 min at 4 °C. Supernatant was discarded and the nuclei were lysed in iCLIP lysis buffer (50 mM Tris-HCl at pH 7.4, 100 mM NaCl, 1% NP40, 0.1% SDS, 0.5% sodium deoxycholate) supplemented with protease inhibitor cocktail (Thermo Fisher, #A32961) for 10 min on ice. This nuclear fraction was then stored at −80 °C.

### RNA extraction and cDNA synthesis

2 million cells were collected and washed with ice-cold PBS. For sequencing, cell pellets were then resuspended in 300 μL of Trizol. RNA extraction was performed by using the Direct-zol™ RNA MicroPrep kit (Zymo Research, #R2062) following the recommendations of the manufacturer. For qPCR analysis, cell pellets were lysed and RNA extracted by using the RNeasy Mini kit (Qiagen, #74136) following the recommendations of the manufacturer. All the extractions were performed in RNase-free conditions. RNA was then quantified by using the Nanodrop. For sequencing, the quantification was performed by using Qbit™ 4 Fluorometer (Thermo Fisher, #Q32852) and the RIN was assessed by using the 4200 TapeStation System (Agilent, #5067-5579). For cDNA synthesis, 300ng-500ng of RNA was converted into cDNA by using the High-Capacity cDNA Reverse Transcription Kit (Thermo Fisher, #4368814) following the supplier’s recommendations. For further analysis by PCR or qPCR, the cDNA final solution was diluted to an equivalent of 1 ng/µL in RNase-free water.

### PCR and quantitative PCR

Splicing analysis, by visualisation of the isoforms, was performed by PCR. 2 ng of cDNA were mixed with OneTaq® Hot Start 2X Master Mix (NEB, #M0484L), 800 nM final of primers, and the reaction volume was topped up to 12.5 µL with water. The amplification in the thermocycler (Biorad) was performed by following the supplier’s recommendation for the Taq polymerase. The melting temperature was determined with the Tm calculator tool provided on the NEB website. After completion, the PCR reaction was mixed with a gel loading dye (NEB, #B7024S) and the reaction was loaded on a 2% agarose gel along with a 100 bp ladder (NEB, #N3231L). Gel pictures are captured with the Amersham^TM^ Imager 600 (GE Healthcare). Splicing analysis was also visualised by capillary gel electrophoresis with QIAxcel analyser (Qiagen) by using the DNA High Resolution Kit (Qiagen, #929002). For quantitative PCR, 5 ng of cDNA were mixed with 5 µl PowerUp™ SYBR™ Green Master Mix (Thermo Fisher, #A25742), and primers at 800 nM final for each reaction. The 384-well plate was placed into the CFX384 Touch Real-Time PCR detection system (BioRad). Primers used for PCR and quantitative PCR are available in Data S6.

### Apoptosis analysis and synergy calculation

Apoptosis analysis was performed by staining ~500,000 cells with an Annexin V-Alexa Fluor 647 antibody (Biolegend, #640912) and DAPI in 1X Annexin V Binding Buffer (BD Bioscience, #556454). Apoptosis was then assessed on a LSRFortessa™ Cell Analyzer (BD Bioscience) and data were analysed using FlowJo™ Software (BD Bioscience). The percentage of viability was uploaded in the SynergyFinder Plus online tool (PMID: 35085776), and synergy was calculated by using the Zero Interaction Potency (ZIP) model (PMID: 26949479).

### Proteostat staining

100,000 cells were treated with 2 µM OPP (Thermo Fisher, # C10459) for 0, 5, 10, 15, 20, 30, or 60 min. A positive control was added with cells treated for 16H with 5 µM MG132 (Merck, M7449). Cells were washed with PBS + 2% FBS, then centrifuged at 320 g for 5 min. Cells were then fixed in 250 µL of Cytofix solution (BD, #554714) for 30 min at 4 °C. Fixed cells were then washed two times with 1X of perm/wash solution (BD, #554714). Finally, the cells were resuspended in 300 µL of 1x Perm/Wash + Proteostat Aggresome Red Detection Reagent diluted at 1/10,000 (Enzo, ENZ-51035-K100), freshly prepared. Samples were protected from light and incubated for 30 minutes at room temperature. Proteostat staining was then assessed on a LSRFortessa™ Cell Analyzer (BD Bioscience) and data were analysed using FlowJo™ Software (BD Bioscience).

### Immunofluorescence

Areas of ~1 cm diameter were drawn with a PAP pen (Merck, #Z377821) on poly-l-lysin slides (VWR, #631-0107). After washing cells with PBS, approximately 150,000 cells were resuspended in 80 µl and were dropped onto the circled areas. Slides were incubated for 30 min at 37 °C. The excess PBS was then removed using a pipette. Cells were fixed with 2% PFA for 10 min at room temperature. After washing the cells three times with PBS, cells were permeabilized with 0.25% Triton X-100 for 30 min at room temperature. Cells were then washed three times with PBS + 2%FBS and blocking was performed with a blocking buffer (PBS, 10%FBS, 2%BSA, 0.1%Triton X-100) for 30 min at room temperature. Afterwards, the primary antibody for SRSF1 (Thermo Fisher, #324500) was diluted at 1:500 in blocking buffer, added to the circled areas and incubated overnight at 4 °C. Then the cells were washed three times with PBS + 2%FBS and incubated with the secondary antibody, AF488 goat-anti-mouse IgG (Thermo Fisher, #A32723), diluted at 1:500 in blocking buffer for 1H at room temperature in the dark. Finally, the cells were washed three times with PBS + 2%FBS and mounted (Merck, #DUO82040). The slides were allowed to dry for 24 hours before analysing the cells using the Confocal LSM 880 Airyscan Zeiss. The settings for image acquisition were kept the same between samples of the same cell line to be able to compare the intensity of the stainings for the different conditions. All pictures were taken with a 63x oil immersion objective.

### Western blot

2 million cells were collected and washed with ice-cold PBS. Cell pellets were then lysed in a protein lysis buffer (1% Igepal, CA-630, 0.1% SDS, 0.5 sodium deoxycholate, 100 mM NaCl, 50 mM Tris HCL at pH 7.4) supplemented with Pierce Protease inhibitor (ThermoFisher, #A32961). Lysates were sonicated using the Bioruptor Pico Sonicator (Diagenode) for 30 cycles, consisting of 30 seconds on and 30 seconds off intervals at 4 °C. The samples were spun down at 12,000 rpm for 10 minutes at 4 °C and the supernatant was collected into a new tube. A protein dosage was then performed using the Pierce BCA Protein Assay Kit (Thermo Fisher, #23225) following the supplier’s recommendations. After quantification, all samples were adjusted to the same protein concentration by dilution with the protein lysis buffer. Lysates were then mixed with 1X NuPAGE™ LDS Sample Buffer (Thermo Fisher, #NP0007), and 1X Reducing Agent (Thermo Fisher, #NP0009). All samples were heated for 5 minutes at 92 °C before loading on a pre-cast Mini Protein gel (Thermo Fisher, #NP0323BOX) along with the PageRuler Plus Pre-stained Protein Ladder (Thermo Fisher, #26619). The Western blot tank was filled with 1X NuPAGE MOPS SDS Running Buffer (ThermoFisher, #NP0001). Protein transfer on nitrocellulose membrane was performed in liquid condition by using the 1X NuPAGE Transfer Buffer (ThermoFisher, #NP00061) supplemented with 10% methanol. The transfer was run overnight at 15 V on ice. Protein transfer was confirmed with a ponceau staining and the membrane was then washed with 1X TBST (Tris-buffered saline with 0.1% Tween 20) until complete staining removal, and the membrane was blocked with 3% BSA in 1X TBST for 1H at room temperature. Incubation with primary antibody, in blocking buffer, was performed overnight at 4 °C. After washing with 1X TBST, the membrane was incubated with a secondary antibody (Cell Signalling Technology, #7074S, #7076S), diluted at 1:10,000 in blocking buffer, for 1H at room temperature. Membrane was washed and then proteins were revealed by adding Pierce ECL Western Blotting Substrate (Thermo Fisher, #32109) on top of the membrane, which was incubated for 3 minutes. The chemiluminescence signals were monitored using the AmershamTM Imager 600 (GE Healthcare). The following antibodies were used: Anti-Phospho-IRE1 (Abcam, #ab48187), Anti-IRE1α (Cell Signalling, #3294), Anti-XBP1s (Cell Signalling, #83418), Anti-cleaved PARP (Cell Signalling, #9541), Anti-Phospho-eIF2α (Cell Signalling, #3398), Anti-eIF2α (Cell Signalling, #9722), Anti-ATF6 (Novus Biologicals, #NBP1-40256), Anti-Puromycin (Merck, #MABE343), Anti-ATF4 (Cell Signalling, #11815), Anti-SRSF1 (Thermo Fisher, #324500), Anti-RBM39 (Proteintech, #67420-1-lg), Anti-AP3S2 (Proteintech, #15319-1-AP), Anti-Phosphoepitope SR proteins (Merck, #MABE50), Anti-UPF1 (Cell Signalling, #12040), Anti-CLK1 (Santa Cruz, #sc-515897), Anti-αTubulin (Abcam, #ab7291), Anti-GAPDH (Cell Signalling, #97166), Anti-Histone H3 (Merck, #PLA0148), Anti-Actin (Merck, #A2066), Anti-HA (Cell Signalling, #2999).

### Sample preparation for phosphoproteomics and peptide methylation and acetylation analysis

Mass spectrometry experiments were performed using mass spectrometry as reported (PMID: 31959955; PMID: 23532336) with some technical modifications. 10 million cells were collected and washed with ice-cold PBS supplemented with phosphatase inhibitors (1 mM Na_3_VO_4_ and 1 mM NaF). Cell pellets were then lysed with a Urea buffer (8 M urea in 20 mM in HEPES, pH 8.0 supplemented with 1 mM Na_3_VO_4_, 1 mM NaF, 1 mM Na_2_H_2_P_2_O_7_ and 1 mM sodium β-glycerophosphate). Lysates were then sonicated in a Bioruptor Pico for 20 cycles, consisting of 30 seconds on and 35 seconds off intervals at 4 °C. Samples were then centrifuged at 13,000 rpm for 10 min at 5 °C. Supernatant was transferred to a 1.5 mL Protein Lo-bind tube (Eppendorf, #Z666505-100EA). A protein dosage was then performed using the Pierce BCA Protein Assay Kit (Thermo Fisher, #23225) following the supplier’s recommendations. Proteins were digested into peptides using trypsin as previously described (PMID: 22547687; PMID: 21316455). For phosphoproteomics, 110 μg of protein in a volume of 300 µL were subjected to cysteine reduction and alkylation by sequential incubation with 10 mM dithiothreitol (DDT) and 16.6 mM iodoacetamide (IAM) for 1 h and 30 min, respectively, at 25 °C with agitation. Trypsin beads (50% slurry of TLCK-trypsin) were equilibrated with 3 washes with 20 mM HEPES (pH 8.0), the urea concentration in the protein suspensions was reduced to 2 M by the addition of 900 µL of 20 mM HEPES (pH 8.0), 100 μL of equilibrated trypsin beads were added and samples were incubated overnight at 37 °C. Trypsin beads were removed by centrifugation (2000 x *g* at 5 °C for 5 min) and samples were transferred to 96-well plates and acidified by adding TFA to a final concentration of 0.1%. Peptide solutions were desalted and subjected to phosphoenrichment using the AssayMAP Bravo (Agilent Technologies) platform. For desalting, the protocol peptide clean-up v3.0 was used. Reverse phase S cartridges (Agilent, 5 μL bed volume) were primed with 250 μL 99.9% acetonitrile (ACN) with 0.1%TFA and equilibrated with 250 μL of 0.1% TFA at a flow rate of 10 μL/min. The samples were loaded (770 μL) at 20 μL/min, followed by an internal cartridge wash with 250 μL of 0.1% TFA at a flow rate of 10 μL/min. Peptides were then eluted with 105 μL of 1 M glycolic acid with 50% ACN, 5% TFA and this is the same buffer for subsequent phosphopeptide enrichment. Following the Phospho Enrichment v 2.1 protocol, phosphopeptides were enriched using 5 µl Assay MAP TiO2 cartridges on the Assay MAP Bravo platform. The cartridges were primed with 100 µl of 5% ammonia solution with 15% ACN at a flow rate of 300 μL/min and equilibrated with 50 μL loading buffer (1 M glycolic acid with 80% ACN, 5% TFA) at 10 μL/min. Samples eluted from the desalting were loaded onto the cartridge at 3 μL/min. The cartridges were washed with 50 μL loading buffer and the phosphorylated peptides were eluted with 25 μL 5% ammonia solution with 15% ACN directly into 25 μL 10% formic acid. Phosphopeptides were lyophilized in a vacuum concentrator and stored at −80 °C. For K and R methylation and acetylation analysis, 110 μg of protein were digested and acidified as described above. Peptide solutions were desalted using the AssayMAP Bravo (Agilent Technologies) platform. Cartridges were primed, equilibrated, loaded and washed as described above and peptides were eluted with 105 μL of 70/30 ACN/H_2_O  +  0.1% TFA. Eluted peptide solutions were dried in a SpeedVac vacuum concentrator and peptide pellets were stored at −80 °C.

### O-Propargyl-Puromycin pull-down

20 million of cells were co-treated with 1 μg/mL tunicamycin for 4H and then with OPP (Thermo Fisher, #C10459) for the last 30 min. Cells were then collected and washed with ice-cold PBS. Cell pellets were then lysed with 900 μL of urea lysis buffer (Jena Bioscience, #CLK-1065) supplemented with Pierce Protease inhibitor (Thermo Fisher, #A32961). Lysates were sonicated by using a Branson tip sonicator for 2 minutes, consisting of 20 seconds on and 30 seconds off intervals, power 50%, at 4 °C. Samples were then centrifuged at 13,000 rpm for 10 min. Supernatant was transferred to a 1.5 mL Protein Lo-bind tube (Eppendorf, #Z666505-100EA). A protein dosage was then performed using the Pierce BCA Protein Assay Kit (Thermo Fisher, #23225) following the supplier’s recommendations. In parallel, the azide-agarose resin (Jena Bioscience, #CLK-1038-2) was prepared. 200 μL of 50% resin slurry was resuspended and transferred to a 1.5 mL Protein Lo-bind tube. The resin was washed with 1.3 mL of distilled water and spun down for 2 minutes at 500 *g*. The supernatant was carefully discarded, and the resin was used for the click reaction. A 2X copper catalyst solution (Jena Bioscience, #CLK-1065) was prepared freshly according to the supplier’s instructions. The click reaction was then performed by mixing 200 μL of washed alkyne-agarose resin, 800 μL of protein extract (~10 mg of protein), 1 mL of 2X copper catalyst solution. The reaction was then incubated on a wheel overnight at 4 °C. The click reaction was transferred to a spin column (G-Biosciences), which was spun down 2 min at 500 *g*. The flow through was discarded. The resin was washed with 2 mL of distilled water, spun down, and the flow-through discarded. The resin was washed several times: 1 time with wash buffer A (Tris 100 mM, 1 M NaCl, pH8.0, 0.1%SDS), 3 times with wash buffer B (Tris 100 mM, 1 M NaCl, pH8.0) and 3 times with wash buffer C (25 mM Ammonium bicarbonate, pH8.0). For each wash, the resin was incubated 10 min at 4 °C on a wheel, the column was centrifuged for 2 minutes at 500 *g* and the flow-through was discarded. Finally, the resin was resuspended in 2 mL of wash buffer C and transferred to a 2 mL Protein Lo-bind tube (Eppendorf, #Z666513-100EA). The resin was centrifuged for 2 min at 500 *g* and the supernatant was discarded. Then, 330 µl of buffer C with 1083.33 ng of soluble trypsin were added and protein solutions were incubated at 37 °C and agitation (1400 rpm). Samples were desalted using C18+ carbon top tips as previously described (PMID: 36843114). Briefly, spin tips were always centrifuged at 1,500 x *g* at 5 °C for 3 min. Column spin tips were activated twice with 200 µL of elution solution (70% ACN, 0.1% TFA) and equilibrated twice with 200 µL of wash solution (1% ACN, 0.1% TFA). Spin tips were loaded with samples and washed twice with wash solution. Finally, spin tips were transferred to new 2 mL Protein LoBind tubes and peptides were eluted 4 times with 50 µl of elution solution, dried in a speed vac and stored at −80 °C.

### LC-MS/MS analysis

Peptides were re-suspended in 20 µL of reconstitution (97% H2O, 3% ACN, 0.1% TFA, 50fmol/µl-1 enolase peptide digest) and sonicated for 5 minutes at RT. Following a brief centrifugation, 2 μl was loaded onto a LC-MS/MS system. This consisted of a nano flow ultra-high pressure liquid chromatography system, UltiMate 3000 RSLC nano (Dionex) coupled to a Q Exactive Plus using an EASY-Spray system. The LC system used mobile phases A (3% ACN: 0.1% FA) and B (100% ACN; 0.1% FA). Peptides were loaded onto a μ-pre-column and separated in an analytical column. The gradient: 1% B for 5 minutes, 1% B to 35% B over 90 minutes, following elution, the column was washed with 85% B for 7 minutes, and equilibrated with 3% B for 7 minutes, flow rate of 0.25 µL/min. Peptides were nebulized into the online connected Q-Exactive Plus system operating with a 2.1 s duty cycle. Acquisition of full scan survey spectra (m/z 375-1500) with a 70,000 FWHM resolution was followed by data-dependent acquisition in which the 15 most intense ions were selected for HCD (higher energy collisional dissociation) and MS/MS scanning (200–2000 m/z) with a resolution of 17,500 FWHM. A 30 s dynamic exclusion period was enabled with an exclusion list with a 10 ppm mass window. Overall duty cycle generated chromatographic peaks of approximately 30 s at the base, which allowed the construction of extracted ion chromatograms (XICs) with at least ten data points.

### Peptide and protein identification and quantification

Peptide identification from MS data was automated using a Mascot Daemon (v2.8.0.1) workflow in which Mascot Distiller generated peak list files (MGF) from RAW data, and the Mascot search engine matched the MS/MS data stored in the MGF files to peptides using the SwissProt Database restricted to the Homo sapiens taxon (SwissProt_2021_02.fasta; 20396 sequences). Searches had an FDR of ~1% and allowed 2 trypsin missed cleavages, mass tolerance of ±10 ppm for the MS scans and ±25 mmu for the MS/MS scans, carbamidomethyl Cys as a fixed modification and oxidation of Met, PyroGlu on N-terminal Gln and phosphorylation on Ser, Thr, and Tyr as variable modifications (phosphorylation was only considered for searches performed in phosphoproteomics data). Identification of peptides with other PTMs was performed using the parameters mentioned above, and allowing methyl R, methyl K, dimethyl K, trimethyl K and acetyl K as variable modifications. Searches for modifications in R and K were performed independently. Pescal was used for label-free quantification of the identified peptides. The software constructed XICs for all the peptides identified in at least one of the LC-MS/MS runs across all samples. XIC mass and retention time windows were ±7 ppm and ±2 min, respectively. Quantification of peptides was achieved by measuring the area under the peak of the XICs. Individual peptide intensity values in each sample were normalized to the sum of the intensity values of all the peptides quantified in that sample. Data points not quantified for a particular peptide were given a peptide intensity value equal to the minimum intensity value quantified in the sample divided by 10. For phosphoproteomics experiments, we obtained a phosphorylation index (ppIndex) by summing the signals of all peptide ions containing the same modification site. For the proteomics experiment, protein intensity values were calculated by adding the intensities of all the peptides derived from a protein. Protein score values were expressed as the maximum Mascot protein score value obtained across samples. Statistical analysis was carried out in Excel or in R (v4.0.0) using base functions or the ggpubr package (https://CRAN.R-project.org/package=ggpubr). For the OPP pull-down experiment, a Spearman correlation coefficient was calculated for each protein by ranking peptide intensities and OPP concentrations from smallest to largest. Proteins with a Spearman coefficient ≥ 0.8 were then shortlisted. Since the bead volume was identical across all conditions and only the OPP concentration varied, this approach helped minimise background noise arising from non-specific bead binding. Term enrichment analysis of subsets of proteins differentially expressed or phosphorylated was performed using the clusterProfiler package (https://bioconductor.org/packages/release/bioc/html/clusterProfiler.html). Data was visualized using the ggplot2 package (https://cran.r-project.org/web/packages/ggplot2/index.html).

### RNA sequencing and analysis

RNA-Sequencing was performed at Novogene with paired-end, 150 bp sequence reads. Library preparation was carried out with Poly-A enrichment and sequencing was performed on a NovaSeq6000 platform, generating on average 41 million reads per sample. Reads were quality trimmed using seqtk v1.2 and aligned to the reference genome GRCh38 (hg38) using STAR v2.7.0 f (PMID: 26334920). Counting of reads was performed with RSEM v1.3.1 (PMID: 21816040) using the ENSEMBL annotation GRCh38.101. Only genes that achieved at least one read count per million reads (TPM) in at least 8 samples 8 were kept. This led to 14,007 sufficiently detected. Differential expression analysis was performed using the ‘limma” R package and voom normalisation (PMID:25605792). Differentially expressed genes were thresholded with padj <= 0.05 and log2 fold-change > |1 | . Gene set enrichment analysis (GSEA) was performed on the publicly available bioinformatics platform GenePattern (PMID:16642009) using GSEA pre-ranked (PMID:16199517) and the ranked t-statistic of all sufficiently detected genes. The analysis was performed for Canonical Pathways and Gene Ontology Biological Processes. Hierarchical cluster analysis was performed on a Pearson’s correlation matrix using the Ward D2 clustering method. Heatmaps were generated using the R package ComplexHeatmap. Row clustering was performed on the Euclidean distance and “complete” clustering method. Differential splicing was examined using rMATS version 4.0.2. Aberrant splicing events were thresholded with FDR = < 0.05 and inclusion level difference >= |0.1|. Enrichment analysis of the genes with significant splicing events was performed using the function dEnricher of the dnet R package. The significantly enriched GO Biological processes with adjp <0.05, along with the adjp values, were used as input in REVIGO (PMID: 21789182). Sashimi plots were generated using IGV (PMID: 36562559). The R package maser was used to illustrate RBM39 isoforms in Supplementary Fig. 3C. RNA-Seq data have been deposited in Gene Expression Omnibus (GEO) under the accession number GSE245918. NMD status presented in Fig. 5C, and the consequences of isoforms switch in Supplementary Fig. 5A, have been generated with the IsoformSwitchAnalyzeR package (PMID:30989184).

### FIMO analysis

Motif Scanning was performed with the FIMO program (PMID: 21330290) available on the MEME Suite 5.5.8. For each splicing event, the skipping exons and spanning introns/exons sequences were retrieved from Ensembl. The FASTA file was then uploaded to FIMO with the RNA Binding motif database (PMID: 23846655). The top 10 of the most represented RNA-binding proteins were compiled in Supplementary Fig. 7A.

### SpliceLearn

The SpliceLearn™ algorithm was used to predict the identity and precise binding locations of splicing factors (SFs) within the ER^splice^ events (PMID: 38664594). SpliceLearn is an XGBoost machine learning model that identifies SF-RNA interactions and their probability of splicing modulatory activity. In addition, SpliceLearn uses Shapley (SHAP, PMID: 32607472) analysis to prioritize SFs most likely to bind at specific nucleotide positions. A probability threshold of 50% was applied, indicating that SF-RNA interactions with over 50% chance of exhibiting splicing modulatory activity were deemed significant. The most predictive SFs were further prioritized using SHAP analysis, and the SFs with the best binding score in the top 50% of features were considered significant (PMID: 38664594). Next, the algorithm generated weighted splicing effect scores for each SF, which were plotted on factor-specific graphs, illustrating the scores for nucleotides where the respective splicing factor is predicted to bind according to the model’s predictions. Additionally, the SFs were ranked within an event using a hypergeometric test. This test measures the enrichment of nucleotides with high alternative splicing effect probabilities for each SF within the event (See Fronk et al., 2024 for more details). For the *RBM39* splicing event, SpliceLearn identified a total of 36 potential splicing factors as binding candidates. Subsequently, this list underwent further refinement based on eCLIP data (enhanced CrossLinking and ImmunoPrecipitation followed by sequencing, PMID: 32728246).

### Quantification and statistical analysis

Unless specified, all data are represented as mean ± standard deviation (SD). Figure legends contain information on independent biological replicates and statistical tests used. All *p-*values are available in the source data files.

### Reporting summary

Further information on research design is available in the Nature Portfolio Reporting Summary linked to this article.

## Supplementary information

Supplementary Information

Description of Additional Supplementary Files

Supplementary Data 1

Supplementary Data 2

Supplementary Data 3

Supplementary Data 4

Supplementary Data 5

Supplementary Data 6

Reporting Summary

Transparent Peer Review file

## Source data

Source Data

## Data Availability

The RNA sequencing data have been deposited in NCBI’s Gene Expression Omnibus and are accessible through the GEO Series accession number GSE245918. Mass proteomics data have been deposited to the ProteomeXchange Consortium via the PRIDE partner repository with the dataset identifier PXD046476 [http://proteomecentral.proteomexchange.org/cgi/GetDataset?ID=PXD0046476]. Source data are provided with this paper.
