# Supplementary material for: PERK orchestrates an endoplasmic reticulum stress alternative splicing program via CLK1/SRSF1
Source: Nat Commun. 2026 Jun 19;17:7745. doi: 10.1038/s41467-026-74397-y (PMC13434015; doi:10.1038/s41467-026-74397-y)
Supplement: Supplementary file 1 [file 41467_2026_74397_MOESM10_ESM.pdf]

nature portfolio

Peer Review File

PERK Orchestrates an Endoplasmic Reticulum Stress Alternative Splicing Program via CLK1/SRSF1

Corresponding Author: Dr Kevin Rouault-Pierre

This file contains all reviewer reports in order by version, followed by all author rebuttals in order by version.

Version 0:

Reviewer comments:

Reviewer #1

(Remarks to the Author)
The manuscript by Philippe et al reports a role for the ER stress sensor PERK in the regulation of alternative splicing. Using O28 Propargyl-puromycin (OPP) labelling and proteomics, they show that ER stress increases the synthesis of splicing factor and identify a the kinase CLK1 as a mechanistic mediator of PERK signaling. I think that this work is original, the finding novel and interesting and the experiments largely convincing. I am supportive of the publication fo this work and have only some technical comments and suggestions. I will go below through the comments, not separating between minor and major ones, but rather in the order they occur through the manuscript.

1- Explain the rationale why OCI-AML3 cells were used. This is not explained.

2- Figure 1F: Why is SRSF1 higher at 24 h in the untreated condition? The increase of SRSF1 after 24h is stronger than the Tm effect for 4 h.

3- I think the name ERsplice is a bit misleading, because it implies that the splicing happens at the ER, which is not what the authors are trying to say. In case the authors want to keep using this name, this should be explicitly made clear.

4- Figure 4: IRE1 knockdown is not very efficient. I suggest that the authors use a stronger siRNA (or shRNA). Otherwise, the lack of an effect could be due to the poor knockdown efficiency.

5- Figure 5G: There is no real effect on AP3S2. I understand that this is statistically significant, but I would say that the magnitude of the effect renders it biologically insignificant.

6- Figure 6B: is there a difference in the enrichment of protein folding when you separate cytoplasmic and ER-lumen folding?

7- Figure 6E: Can a better gel be shown for ARIH2? This is not a very clear and nice example.

8- I think this work should discuss other apers that connected splicing to ER-proteostasis. There are a few papers that are completely ignored by the authors. For instance, this paper is relevant to discuss: PMID: 38836349. This paper shows that IRE1 can regulate the Translation of TOP-Motif Transcripts. Although not directly related to splicing I think it is relevant to include.

Reviewer #2

(Remarks to the Author)
Comments to the Authors

This manuscript addresses how ER stress influences alternative splicing and identifies the SRSF1–CLK1 axis as a potential regulatory node in this process. The topic is timely, as stress-responsive splicing is still less well understood than transcriptional responses, and the authors aim to extend this concept to the unfolded protein response (UPR). The study includes proteomic, phosphoproteomic, RNA-seq, and imaging data. While the work is ambitious, several conceptual and

technical issues substantially weaken the current conclusions and should be carefully addressed.

Major Conceptual Concerns

1. The authors argue that ER stress induces synthesis of new splicing factors, but this conceptual model is not convincing. Canonical SR proteins are abundant, highly stable, and enriched in the nucleus; cells generally regulate their activity through post-translational modification, not new production.

Have the authors measured protein half-lives of splicing factors under UPR conditions?
Is there evidence for preferential degradation during ER stress that would necessitate new synthesis?
Are specific isoforms induced? Many splicing factors generate autoregulatory isoforms via NMD.

Without such evidence, the conclusion that newly synthesized splicing factors contribute to acute splicing changes is not well supported.

2. The manuscript mixes two mechanistically distinct regulatory pathways. It conflates two processes:

Translation of splicing factors (a slow, targeted response), and
Activation of splicing factors by kinases such as CLK1 (a fast, broad response).

The phosphoproteomic results strongly suggest that CLK1 activation—which is known from other stress contexts—is the more plausible driver of rapid splicing changes upon ER stress. These pathways should be clearly separated in the interpretation of results.

3. RNA-seq was performed at 4 h and 24 h of tunicamycin treatment. The 4 h time point is unlikely to capture effects of newly synthesized splicing factors, as translation, nuclear import, and engagement with nascent transcripts would require more time.

The 24 h time point, while allowing de novo synthesis, carries a high risk of capturing indirect effects of prolonged UPR.

A kinetic rationale should be added, particularly distinguishing fast kinase-mediated responses from slower translational ones.

4. The authors overstate the concept of an “ER splice signature” (ERsplice). The term “signature” suggests strong reproducibility and directionality across cell lines, but: Out of >2000 events, only 8 are shared and PERK-responsive. Among four validated candidates, two appear to be stabilized NMD isoforms due to decreased translation rather than changes in splicing.

Figure 5H shows dramatic cytoplasmic accumulation, but little nuclear change—raising questions about whether these are true splicing events or also stabilized NMD targets. The conclusions about a conserved ER splice program should be tempered.

5. The Puromycin pulldown + LC-MS/MS approach is elegant, but interpretation requires more nuance. If new synthesis of SR proteins is indeed detected: Are these changes robust across replicates?
Could they reflect translational recovery or changes in global translation rather than specific induction? Supporting experiments (half-life measurements, isoform analyses) are needed.

6. The synergy in cell death is interesting, but the manuscript lacks mechanistic insight. Does the ER-induced splice program sensitize cells to RBM39 depletion? Are the affected exons enriched in pathways related to protein folding or apoptosis? More mechanistic depth is needed.

7. The discussion is too shallow and should include limitations of the study. For example, CLK1 activation during the UPR is shown, but its dependence on PERK is not established. While CLK1 likely contributes to a subset of splicing events, many observed transcript changes appear to stem from stabilization of NMD targets, not active alternative splicing. The authors should discuss whether the identified isoforms have functional relevance for ER stress survival or adaptation.

8. Minor Comments

Figure 1 – The upper band in the SRSF1 western blot is unlikely to represent SRSF1. Authors should validate identity via siRNA knockdown or use of phospho-SRSF1–specific antibodies.

The lower band’s mobility shift at 24 h is also unexpected given CLK1-mediated hyperphosphorylation, which typically increases apparent molecular weight.

Since SRSF1 autoregulates itself via 3'UTR splicing and NMD, the authors should examine potential isoform changes.

The statement "increase in skipping exon inclusion" is contradictory.
Exon inclusion and exon skipping are mutually exclusive and should be described carefully.

RNA-binding proteins often contain regulatory exons producing NMD isoforms. Among the 33 shared events: How many correspond to NMD-linked exons? Are RBP autoregulatory circuits enriched?

Supplementary Figure 3 - Several panels appear to be missing and should be included for completeness.

Figure 6 – The authors claim that ER stress releases SRSF1 from nuclear speckles and that CLK1 inhibition rescues this. Published studies (PMID: 39755675, 40716777, 36620874) show that other stresses (e.g., hypoxia) upregulate CLK1 and disperse SRSF5/6. These should be discussed to place findings into context.

Figure 6 - The microscopy images do not show canonical nuclear speckles. Instead, SRSF1-GFP accumulates in nucleoli, which is common upon protein overexpression. This undermines the claim that speckle disassembly is occurring. Endogenous staining or lower expression levels would be required.

Figure 7 – The authors search for SRSF1 motifs in flanking exons, but SRSF1 generally binds within alternative exons to promote inclusion. Motif enrichment should be re-analyzed in the alternative exons themselves.

Reviewer #3

(Remarks to the Author)
To the authors:
In the manuscript entitled „PERK orchestrates an endoplasmic reticulum stress alternative splicing program via CLK1/SRSF1", Philippe et al. use OPP labeling to identify newly synthesized proteins after triggering the unfolded protein response (UPR). Among these proteins the authors find numerous proteins involved in RNA processing and splicing. RNA sequencing after UPR activation reveals thousands of splicing pattern changes of which only a tiny fraction is shared between treatments and the cell lines employed. The authors claim that these changes to alternative splicing represent a signature specific to ER stress and demonstrate that they are mediated by activation of the eIF2alpha kinase PERK. While at least two of the UPR-induced mRNA isoforms (PRR3 and AP3S2 mRNA isoforms) represent NMD substrates that are stabilized upon activation of PERK, the other changes to alternative splicing are sensitive to inhibition of CLK1 or knockdown of SRSF1.

General Critique:
OPP-labelling and LC-MS/MS:
I commend the authors for using the OPP labelling approach to investigate protein translation upon activation of the UPR. However, I disagree with their statement that "due to the dynamic nature […] and ribosome stalling, investigating translation upon stress has proven to be challenging" and that "therefore our understanding of […] translatome rewiring upon stress remains limited" (l. 25-27). We and others have published comprehensive datasets that specifically address translation after pharmacological and genetic activation of the UPR employing different and complementary methodology under various, different conditions (e.g. Reich et al., 2020; Klann et al., 2020; Andreev et al., 2015 just to name a few). In this context, it would be very helpful if the authors would compare their dataset to the previously published ones, given that they use yet another methodological approach. What's the degree of overlap? What are the key differences? And if there are, what's the reason for those differences?

Regarding ribosome stalling upon activation of the UPR: we do not observe induction of specific ribosome pause sites in ribosome profiling data; also, there is no increase of disomes after RNaseI treatment which would be indicative of collisions due to stalling. Arguably eIF2alpha phosphorylation mediated by any of the eIF2alpha kinases (among them PERK - activated by the UPR- and GCN2 -activated e.g. by the ribotoxic stress response) is involved in resolving ribosome collisions by reducing ribosome loading on mRNAs presumably also helping to resolve stalling. In light of this, OPP tagging does not offer any obvious advantages over ribosome profiling (in particular when those data are additionally validated by changes in steady state protein levels detected shotgun MS). While I very much appreciate the alternative methodology to monitor translation output under stress, the authors should down tone their claims about the advantages of OPP tagging.

Even after scrutinizing the manuscript, it still remains unclear to me how the OPP experiment was conducted. In the main body of the manuscript and the Materials and Methods section, the authors do not mention any negative controls or disclose how they bioinformatically generated the list of 350 proteins that "significantly correlated […] with OPP concentration upon ER stress".
Why did the authors use correlation with OPP concentration as a criterium in the first place? Even proteins whose translation is attenuated upon stress should exhibit this correlation. Of interest are rather the **changes in translation rate** upon induction of the UPR and the **overall translation rate** during the UPR, which need to be determined relative to control cells. This important control – a control treatment – is mentioned only in the legend to figure S1G: log2 fold change of TM treatment over vehicle (DMSO). Unfortunately, Table 1 lacks all the relevant information (such as e.g. FC relative to control, Z-score etc.) and only provides the identity of the 350 genes.
Finally, induction of the UPR does not entirely shut-off translation and most mRNAs in the cell still produce protein, albeit at a reduced rate. These should also be detectable among the "bona fide proteins de novo synthesized upon endoplasmic

reticulum stress” (l. 85). Why do the authors then come up with a list comprising only 350 proteins? Surely, some bioinformatic sorting/scoring has been performed which is not disclosed in the manuscript.
And why don’t they identify ATF4 among the highly translated proteins? They comment on this in l. 104-105. However, without a proper description of how their data was derived, I cannot follow this reasoning.
In summary I am truly puzzled by the experiment, most likely because important information is lacking from the manuscript.

Splicing signature and activation of CLK1:
The changes to AS observed by the authors in two different cell lines and at two different time points after induction of the UPR are hardly similar – only less than 10% (of each individual treatment) are shared, a rather poor correlation and far from convincing. Would this not be in the range of a spurious overlap between two completely unrelated treatments? The authors should compare to another type of stress (genotoxic stress, hypoxia, starvation, heat shock, or else) to show that the changes observed upon TM treatment are significant.
I very much appreciate the efforts the authors made to demonstrate how the UPR (via PERK) impacts on alternative splicing. However, it remains unclear, how PERK activity results in activation of CLK1. Is it a direct downstream target? Likely not given that SR protein phosphorylation peaks at 4h after induction of the UPR and not earlier. Does activation of another eIF2Alpha kinase (e.g. GCN2, HRI, PKR, or MARK2) produce a similar splicing pattern? Does treatment with e.g. salubrinal recapitulate these changes and can they be blunted by ISRIB? In other words, are those changes to AS mediated by eIF2alpha phosphorylation, or driven by another PERK downstream target such as Nrf2? This would also address the question whether the particular changes to AS are truly ER stress specific as suggested by the authors (named ER^{splice}), or shared with other pathways of the ISR.
Does the LC-MS/MS analysis capture peptides indicative of the changes to AS? If so, what’s the faction of the protein isoform produced by alternative splicing relative to the overall amount of the respective protein (could also be addressed by WB analysis)? Also, the authors have not addressed the effect that the changes to AS might have on the function of the respective encoded proteins (potential LOF, GOF, competitive inhibitor etc.).

Biological Importance of the observed alternative splicing (AS) events:
Unfortunately, also the biological importance of the observed changes to AS remains vague. Increased apoptosis upon induction of the UPR in combination with SRSF1 knockdown or tinkering with RBM39 does not convincingly demonstrate that both are causally related – in particular since negative controls are lacking entirely. Pushing cells further to the limit by pharmacological activation of the UPR typically sensitizes them to various other types of challenges and increased apoptosis. The observed effect upon combined treatment therefore does not necessarily reflect a direct interaction. Hence, I am not convinced that the authors have discovered an “new pro-survival facet of ER stress” (l. 35). More data and refined experimentation are needed to mount a convincing case.

Minor points:
l. 94: typo- should read cycloheximide instead of cycloheximide
l. 134: the event is called exon skipping
l. 136: what’s an inclusion level of -0.66%; percentage of inclusion or fold change would be more informative
l. 144: a venn diagram is not an analysis but rather a way to depict data
l. 147 & 184: what do the authors mean by “identical coordinates”? I can only assume that it is the exact same change to AS?
l. 150 & 151: “skipping exon inclusion” and “increase in skipping exon inclusion” should be rephrased
l. 197ff: what the authors observe are transcriptomic changes; hence, it is not translation reprogramming sensu stricto
l. 232: there are many more splicing factors than the SR proteins. These just represent on particularly well studied group.
l. 273: Looks to me that SRSF1 is not THE top RBP in the list. By eyeballing hnRNPU and hnRNPA1 show higher scores overall in Fig 7A
Figs 2G and S2C: depict the same data just plotted differently. One can be omitted entirely
Figs 3c and D: essentially similar results, one panel could move into the supplement
Fig 5A: I am truly puzzled by the model put forward by the authors…
Fig S2D: This is not a sashimi plot, it’s read depth plot, only
Fig S2F: mentioned in legend but missing

Best wishes, Jan Medenbach

Version 1:

Reviewer comments:

Reviewer #1

(Remarks to the Author)
I think the authors responded well to my initial comments. I think that we already have enough acronyms for major processes and I dont see really a need for more acronyms. On the other hand, I see that many authors like to come up with new acronyms for effects they discovered. ERi-splice is acceptable and less misleading than the original name. As for the

knockdown efficiency, 70% is still not a very strong knockdown, and a knockout should have been attempted. However, in light of the otherwise very strong evidence, I think the manuscript is as a whole very convincing.

Reviewer #2

(Remarks to the Author)
I appreciate the effort that the authors undertook to revise the manuscript according to my suggestions. It really improved. I have no further comments.

Reviewer #3

(Remarks to the Author)
I commend the authors for the thorough revision of the manuscript and the additional data that strengthen the claims in the manuscript. Importantly, the authors now provide data that demonstrate that the identified splice signature can be induced in various different cell lines (Figure S3E & F) and that it is stress type specific: heat shock, hypoxia, genotoxic drugs, or amino acid starvation do not recapitulate the alternative splicing changes observed in the four critical transcripts after induction of the UPR (Figure S4D-F).

Is the splicing pattern truly UPR-specific or part of the ISR?
The authors provide additional evidence that the observed alternative splicing patterns of PRR3, RBM39, AP3S2, and ARIH2 are driven by stress-induced phosphorylation of eIF2alpha by PERK. Notably, knock down of the PERK downstream target NRF2 has no effect, while treatment with ISRIB mitigates the changes to alternative splicing (note to the authors: ISRIB treatment does not relieve Tm-induced ER stress [p25 of the rebuttal] but overcomes the effect of low to moderate phosphorylation of eIF2alpha).
What remains to be addressed is, however, whether other stress kinases that also phosphorylate eIF2alpha S51 can induce similar changes to alternative splicing. Unfortunately, the authors do not show whether GCN2 has been activated after withdrawal of glutamine from the tissue culture medium (Fig S4F) since eIF2 phosphorylation has not been experimentally addressed. Before claiming that the observed splicing signature is indeed ER stress-specific (as suggested by the name $ER^{i\text{-}splice}$) it needs to be convincingly demonstrated that the activation of the other eIF2 kinases does not result in similar changes to splicing (hence not part of the ISR). Specific activation of other eIF2 kinases (GCN2, HRI, PKR, and MARK2) by e.g. pI:pC (PKR), BTdCPU (HRI) or the use of salubrinal would solve this issue.
I do not consider these experiments critical for publication, though. To get semantics right, the authors could instead of $ER^{i\text{-}splice}$ simply choose a different acronym that does not imply that the changes to AS are specific to activation of the UPR.

Open Access This Peer Review File is licensed under a Creative Commons Attribution 4.0 International License, which permits use, sharing, adaptation, distribution and reproduction in any medium or format, as long as you give appropriate credit to the original author(s) and the source, provide a link to the Creative Commons license, and indicate if changes were made.
In cases where reviewers are anonymous, credit should be given to 'Anonymous Referee' and the source.
The images or other third party material in this Peer Review File are included in the article’s Creative Commons license, unless indicated otherwise in a credit line to the material. If material is not included in the article’s Creative Commons license and your intended use is not permitted by statutory regulation or exceeds the permitted use, you will need to obtain permission directly from the copyright holder.

To view a copy of this license, visit https://creativecommons.org/licenses/by/4.0/

We thank the reviewers for their thoughtful and constructive feedback.

Reviewer #1 (Remarks to the Author)

The manuscript by Philippe et al reports a role for the ER stress sensor PERK in the regulation of alternative splicing. Using O28 Propargyl-puromycin (OPP) labelling and proteomics, they show that ER stress increases the synthesis of splicing factor and identify a the kinase CLK1 as a mechanistic mediator of PERK signaling. I think that this work is original, the finding novel and interesting and the experiments largely convincing. I am supportive of the publication of this work and have only some technical comments and suggestions. I will go below through the comments, not separating between minor and major ones, but rather in the order they occur through the manuscript.

1- Explain the rationale why OCI-AML3 cells were used. This is not explained.

Our laboratory has extensive expertise in models of hematological malignancies such as leukemia. Despite the importance of endoplasmic reticulum (ER) stress in cancer cell adaptation, the translational changes induced by ER stress remain poorly characterized in leukemic cells. We used OCI-AML3 cells harbouring mutations in *DNMT3A* and *NPM1*, two of the most commonly mutated genes in leukemia. We also carried out parallel experiments on these cells with aracytine treatment (frontline treatment in leukemia) and OPP labelling to induce and unveil *de novo* proteins synthesised upon treatment, essential to cell adaptation and survival, to identify novel candidates for targeted therapies (not shown in this manuscript). Here, we report a conserved stress-induced splicing signature shared across healthy and malignant cells. Notably, most studies of stress-induced transcriptome and translatome changes have been performed in adherent cells. Our work, therefore not only offers novel insights but also provides a valuable resource for understanding how suspension and adherent cells differ in their acute stress responses.

2- Figure 1F: Why is SRSF1 higher at 24 h in the untreated condition? The increase of SRSF1 after 24h is stronger than the Tm effect for 4 h.

We thank the reviewer for this comment, which highlights an important parameter that we routinely consider when culturing our cells. OCI-AML3 cells have a doubling time of approximately 24 hours; they are seeded at 300,000 cells/mL and typically reach ~600,000 cells/mL after 24 hours. In the context of ER stress studies, cell density is a critical variable, as increased cell concentration can itself induce ER stress, even under control conditions.

This is illustrated in the graph below, which shows increased expression of ER stress markers (*CHOP*, *GADD34*, *BiP*, and *XBP1s*) as cell density rises. We therefore propose that this basal activation of ER stress at 24 hours may account for the baseline induction of SRSF1 observed in **Fig. 1F**.

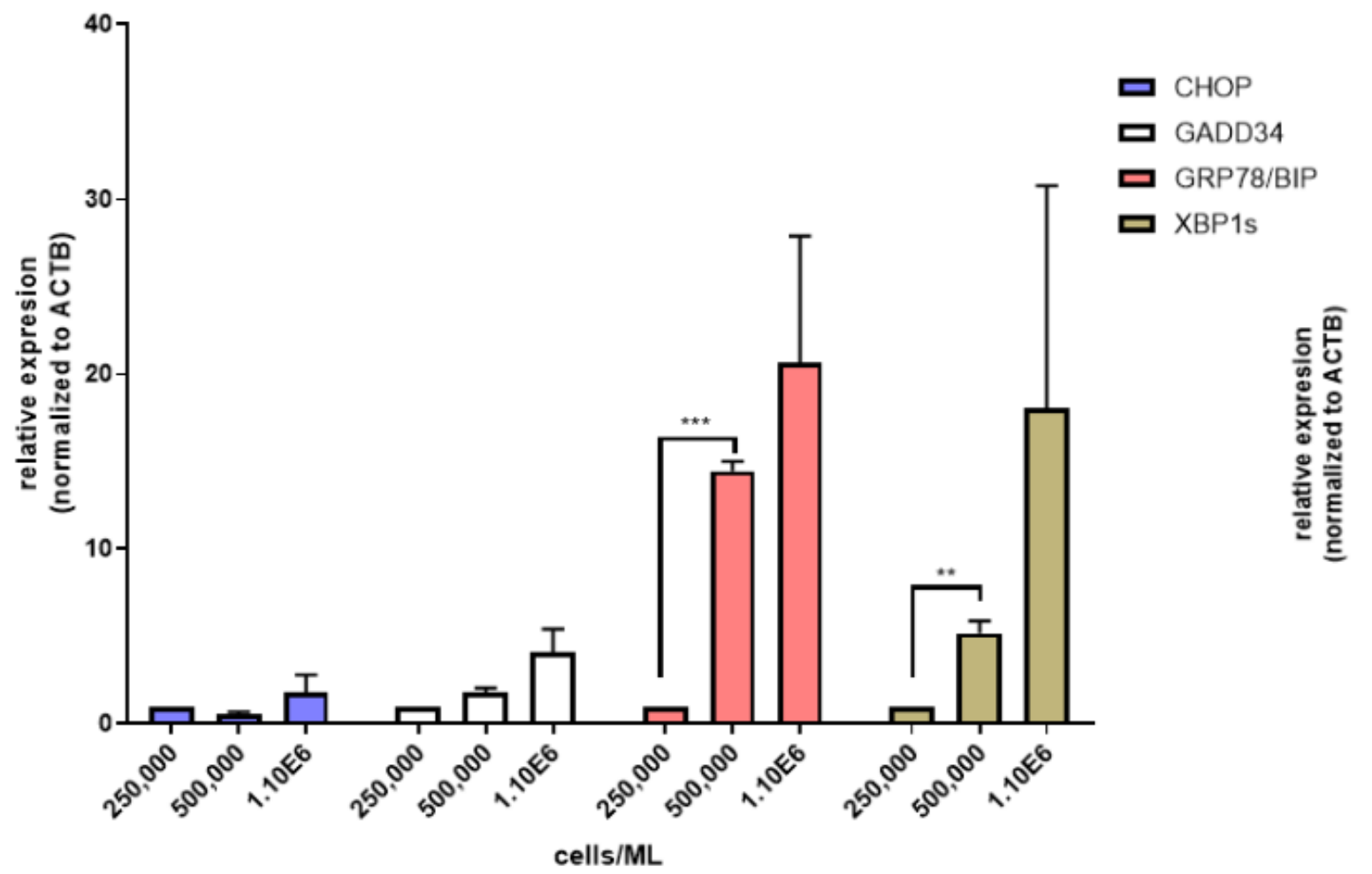

3- I think the name ERsplice is a bit misleading, because it implies that the splicing happens at the ER, which is not what the authors are trying to say. In case the authors want to keep using this name, this should be explicitly made clear.

Indeed, the ER splice signature contains alternatively spliced (AS) and NMD-sensitive isoforms induced by an ER stress, although these splicing events do not occur at the ER itself. To clarify this distinction, we have now adopted the term $ER^{i\text{-}splice}$ for ER induced splice/isoforms accumulation.

4- Figure 4: IRE1 knockdown is not very efficient. I suggest that the authors use a stronger siRNA (or shRNA). Otherwise, the lack of an effect could be due to the poor knockdown efficiency.

We improved IRE1 knockdown efficiency by increasing the siRNA concentration from 50nM to 100nM for 48h, resulting in an increase in silencing from ~50% to ~70% (**see figure below**). Importantly, these results are consistent with our previous data and further support our conclusion that the isoform switch observed upon ER stress induction is independent of IRE1.

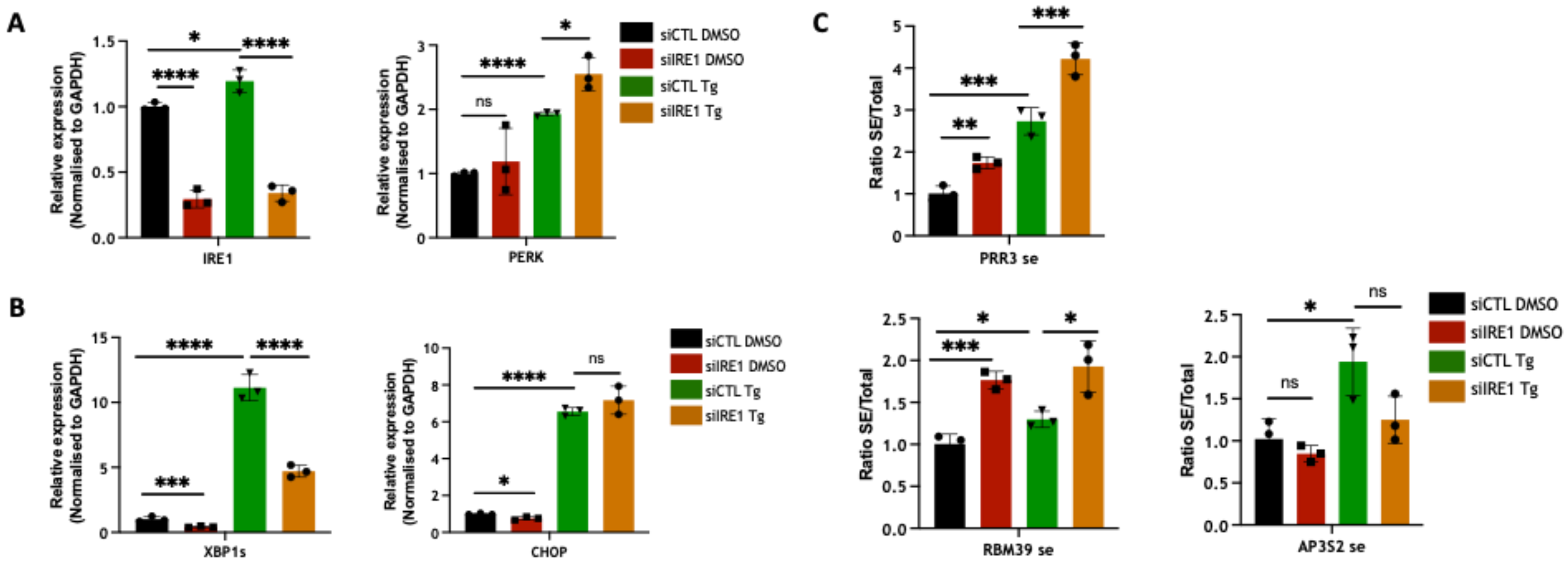

(A) Quantitative PCR analysis of *IRE1, PERK*, and (B) *XBP1s, CHOP* mRNA in HEK293T cells that have been transfected with either siRNA control (CTL), siRNA or siRNA IRE1 for 48H, and then treated with 250nM thapsigargin for 4H. Data are normalized to GAPDH (n=3). mean± S.D. * $p<0.05$, ** $p<0.01$, *** $p<0.005$, **** $p<0.001$. (C) Quantitative PCR of four alternative splicing events (*PRR3, ARIH2, RBM39 and AP3S2)* occurring in HEK293T cells treated with 250nM thapsigargin for 4H and transfected with either siRNA control (CTL), or siRNA IRE1 for 48H. Data are normalized respectively to total *PRR3, ARIH2, RBM39 and AP3S2* (n=4). mean± S.D. * $p<0.05$, ** $p<0.01$, *** $p<0.005$, **** $p<0.001$.

5- Figure 5G: There is no real effect on AP3S2. I understand that this is statistically significant, but I would say that the magnitude of the effect renders it biologically insignificant.

We've now edited the text to moderate our conclusion on this specific figure (**line 236**), although it is worth noticing that this conclusion is confirmed by the observations **Fig. 5H** where *AP3S2* transcript does not accumulate in the nucleus. See **line 236**, "although the effect on *AP3S2* is subtle".

6- Figure 6B: is there a difference in the enrichment of protein folding when you separate cytoplasmic and ER-lumen folding?

We thank the reviewer for this comment. To address this point, we extracted the list of protein associated with the 'Protein Folding' term shown in **Fig. 6B** (CCT3, PDIA3, HSPA9, HSP90AB3P, HSPA8, HSP90AA1, HSP90AB1, HSPE1, PDIA6, HSPD1, PDIA4, HSP90B1, ERP44, HSP90B2P, KHSRP, TCP1, DNAJB11, P4HB, PPIB, PPIA).

We then performed a cellular component analysis based on this list to discriminate ER folding proteins from cytoplasmic folding proteins:

Sublist	Category	Term	RT	Genes	Count	P-Value	Benjamini
▢	UP_KW_CELLULAR_COMPONENT	Endoplasmic reticulum	RT	40.00%	8	2.78e-4	3.34e-3
▢	UP_KW_CELLULAR_COMPONENT	Cytoplasm	RT	50.00%	10	9.95e-2	5.97e-1

- **ER folding proteins:** DNAJB11, ERP44, HSP90B1, PPIB, P4HB, PDIA3, PDIA4, PDIA6
- **Cytoplasmic folding proteins:** CCT3, HSP90AA1, HSP90AB1, HSP90AB3P, HSPA8, HSPA9, HSPD1, PPIA, TCP1, KHSRP

These results show that both ER-localized and cytoplasmic protein folding machineries are enriched, revealing a global stress response.

7- Figure 6E: Can a better gel be shown for ARIH2? This is not a very clear and nice example.

We have now updated **Fig. 6E** with a better PCR gel for *ARIH2* and *RBM39* (**please see figure below**).

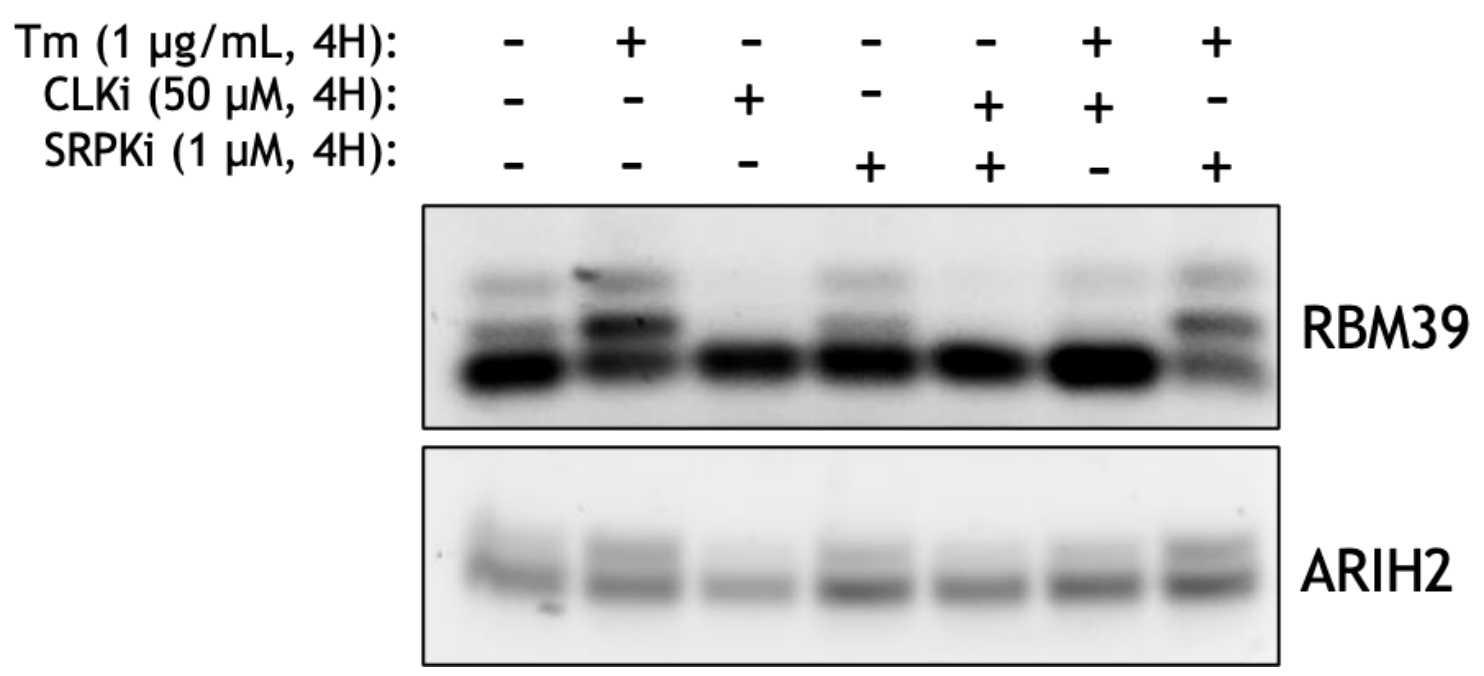

Representative gel PCR showing the alternative splicing events for two of the splicing events (*RBM39 and ARIH2*) occurring in OCI-AML3 treated with tunicamycin at 1µg/mL for 4H, +/- CLK or SRPK inhibitors, used respectively at 50µM or 1µM in co-treatment.

8- I think this work should discuss other papers that connected splicing to ER-proteostasis. There are a few papers that are completely ignored by the authors. For instance, this paper is relevant to discuss: PMID: 38836349. This paper shows that IRE1 can regulate the Translation of TOP-Motif Transcripts. Although not directly related to splicing I think it is relevant to include.

We thank the reviewer for their suggestions. We have now amended the manuscript (**lines 49-50**).

Reviewer #2 (Remarks to the Author):

Comments to the Authors

This manuscript addresses how ER stress influences alternative splicing and identifies the SRSF1–CLK1 axis as a potential regulatory node in this process. The topic is timely, as stress-responsive splicing is still less well understood than transcriptional responses, and the authors aim to extend this concept to the unfolded protein response (UPR). The study includes proteomic, phosphoproteomic, RNA-seq, and imaging data. While the work is ambitious, several conceptual and technical issues substantially weaken the current conclusions and should be carefully addressed.

Major Conceptual Concerns

1. The authors argue that ER stress induces synthesis of new splicing factors, but this conceptual model is not convincing. Canonical SR proteins are abundant, highly stable, and enriched in the nucleus; cells generally regulate their activity through post-translational modification, not new production. Have the authors measured protein half-lives of splicing factors under UPR conditions? Is there evidence for preferential degradation during ER stress that would necessitate new synthesis?

We thank the reviewer for raising this important conceptual point. While SR proteins are indeed generally abundant, stable, and primarily regulated through post-translational modifications, our data suggest that their expression can also be modulated at the level of protein synthesis under stress conditions.

As a proof of concept, focusing on SRSF1 as a representative SR protein, we employed two orthogonal approaches to assess its translational regulation during ER stress.

First, we assessed the half-life of SRSF1 under tunicamycin-induced stress by performing a cycloheximide chase experiment in OCI-AML3 cells treated with 50 µg/mL cycloheximide. By monitoring the decay of both SRSF1 bands over time, we determined that SRSF1 has an

estimated half-life of approximately 8h under tunicamycin treatment (**see figure below; now included as Supplementary Fig. 1L&M in the manuscript**).

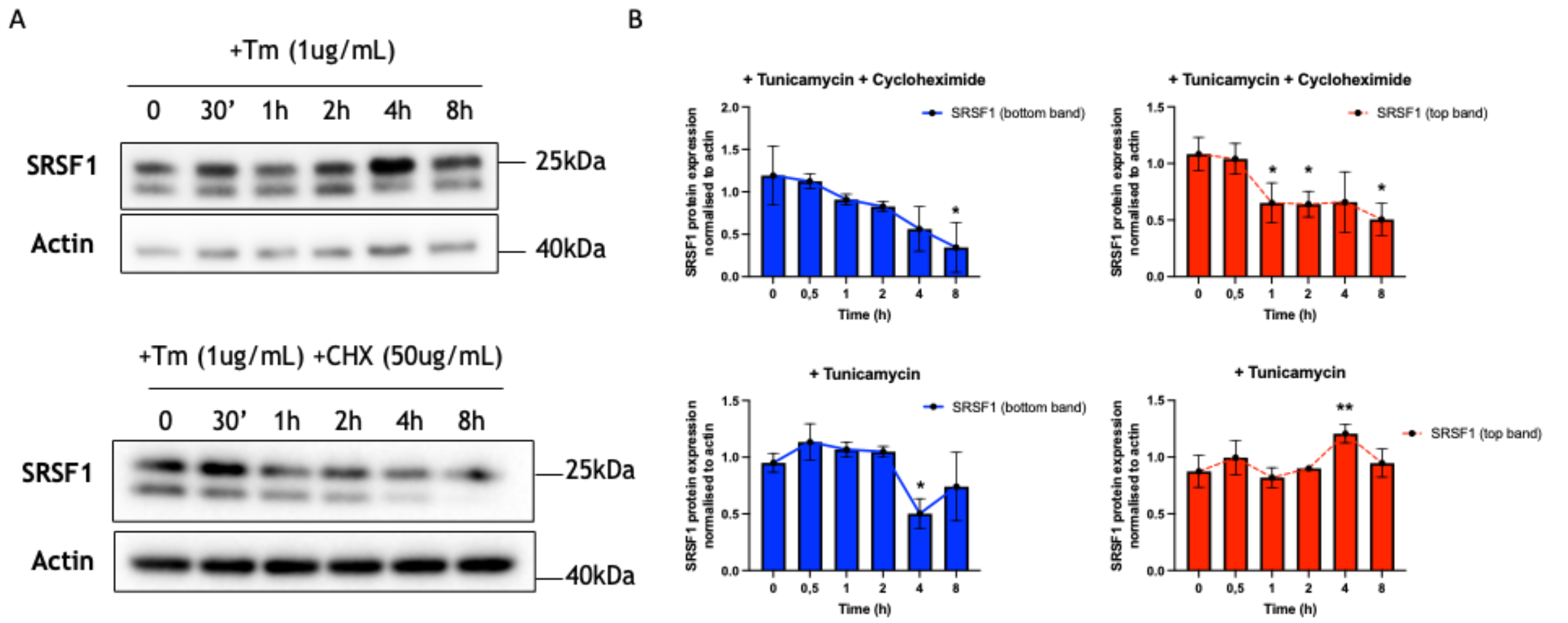

(A) Representative western blot of SRSF1 performed on OCI-AML3 treated with 1ug/mL tunicamycin +/- cycloheximide, for 0, 30min, 1H, 2H, 4H , and 8H. Actin is used as loading control. (B) Quantification of the protein expression level of SRSF1 (bottom band quantification in blue and top band quantification in red) normalised to Actin at the different time point and for the different conditions. (n=3). mean± S.D. **p<0.05, *** *p<0.01*.

Second, we used ISRIB, which rescues translation downstream of eIF2α phosphorylation by promoting the GDP to GTP exchange to restore the pool of active eIF2α, thereby counteracting translational repression during the ER stress response. OCI-AML3 cells were treated with 1 µg/mL tunicamycin ± 400 nM ISRIB for 4 hours and pulsed with 2 µM puromycin for 30 minutes. Following immunoprecipitation with an anti-puromycin antibody, we performed a western blot for SRSF1 and puromycin.

Quantification of the SRSF1-to-puromycin signal ratio in the immunoprecipitates revealed an increase in newly synthesised SRSF1 upon tunicamycin treatment, which was reversed by ISRIB (**see figure below**). These results demonstrate that SRSF1 is actively translated during ER stress.

Altogether with our half-life experiment, these results support the conclusion that SRSF1 expression is dynamically regulated during ER stress at the level of protein synthesis.

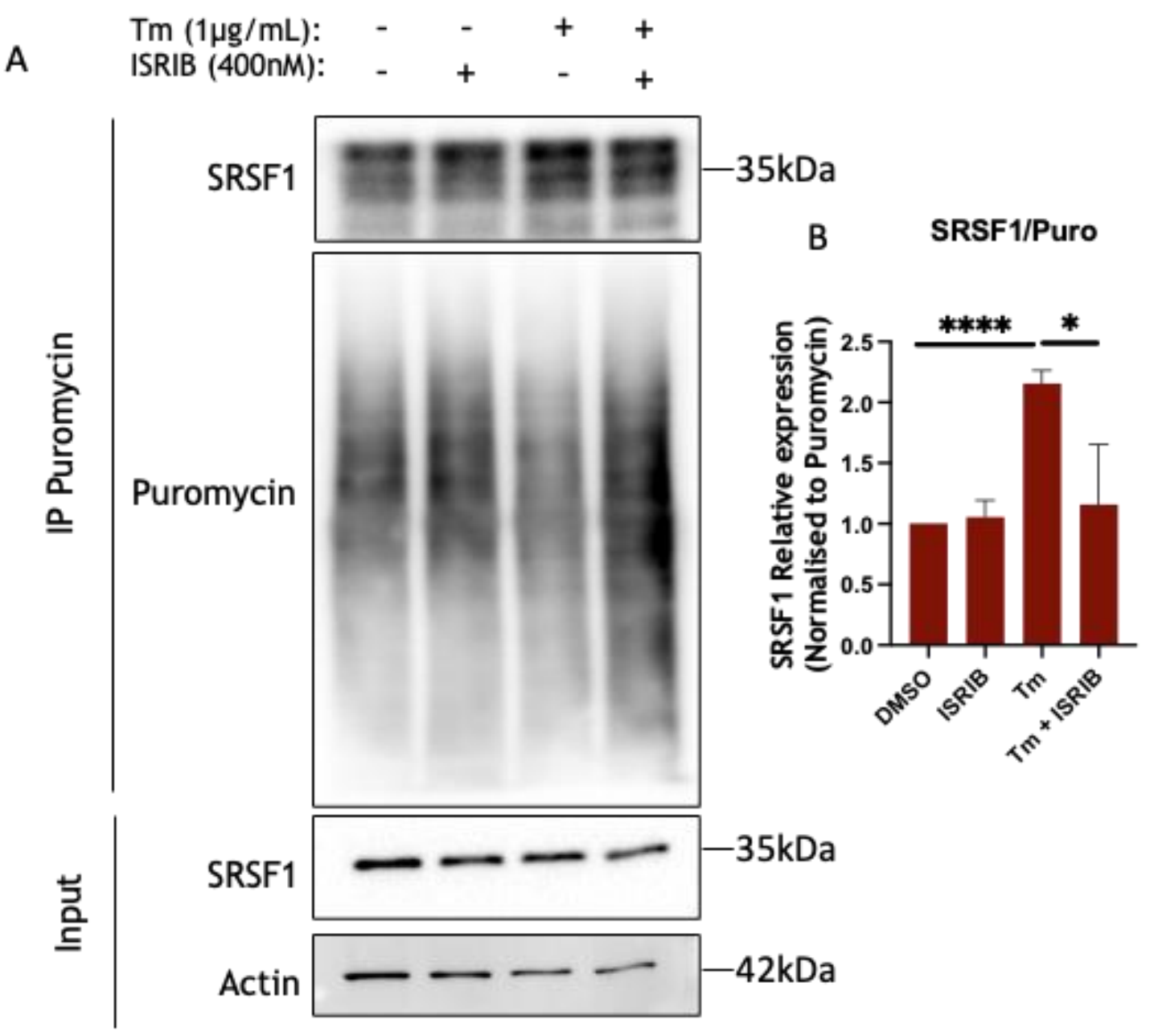

(A) Representative western blot performed following immunoprecipitation with puromycin antibody in OCI-AML3 treated with 1ug/mL tunicamycin +/- 400nM ISRIB, for 4H, and 2uM puromycin for 30min prior cells collection. Immunoprecipitates were immunoblotted for SRSF1 and Puromycin. Total input was immunoblotted for SRSF1 and Actin, as loading control. (B) Quantification of the protein ratio SRSF1 to Puromycin signals in the immunoprecipitates is depicted on the graph. (n=3). mean± S.D. *$p<0.05$, **** $p<0.001$.

Are specific isoforms induced? Many splicing factors generate autoregulatory isoforms via NMD. Without such evidence, the conclusion that newly synthesized splicing factors contribute to acute splicing changes is not well supported.

The remark is highly relevant. To address this point we investigated whether specific isoforms of splicing factors, particularly those subject to autoregulatory NMD mechanisms, are induced upon ER stress. We cross-referenced the splicing analysis and the OPP pull-down results with a focus on RNA-binding proteins (**please see figure A below**). This analysis did not identify any overlapping targets, indicating that RBPs exhibiting altered translation in the OPP assay do not appear to undergo alternative splicing under these conditions.To further explore this, we performed an analysis of SRSF1 isoforms by using the IsoformSwitchAnalyzeR package. Consistent with the global analysis, we did not detect any significant switches in *SRSF1* isoforms upon tunicamycin treatment (**please see figure B below**).

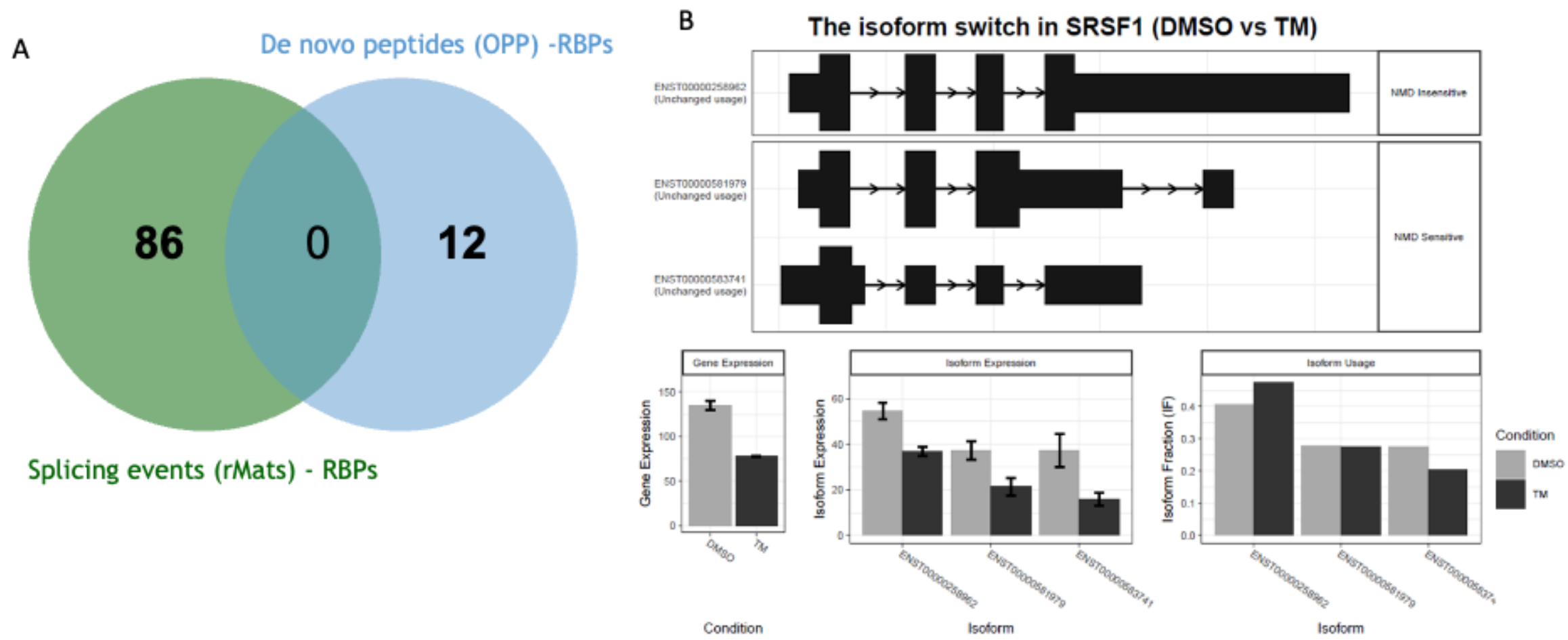

(A) Venn diagramm showing the overlap in RNA Binding Proteins encoded genes commonly affected by splicing detected by rMats, with Inclusion Level>I0.2I, *pvalue*<0.05, or captured with OPP pull down, *Spearman coefficient* ≥ 0.8 . (B) Graphs depicting the different SRSF1 isoforms found in OCI AML3 by using the IsoformSwitchAnalyzeR package (top panel). SRSF1 gene expression, isoforms expression and isoforms usage are represented by histograms in DMSO and tunicamycin (TM) treated OCI AML3 for 4H (bottom panel).

2. The manuscript mixes two mechanistically distinct regulatory pathways. It conflates two processes:

- Translation of splicing factors (a slow, targeted response), and Activation of splicing factors by kinases such as CLK1 (a fast, broad response).

- The phosphoproteomic results strongly suggest that CLK1 activation—which is known from other stress contexts—is the more plausible driver of rapid splicing changes upon ER stress. These pathways should be clearly separated in the interpretation of results.

We thank the reviewer for this relevant comment, and we seize this opportunity to clarify our model. Here, we propose that the splicing rewiring upon stress is the result of a change in stoichiometry of the RBPs during stress **and** phosphorylation of key splicing factors by CLK1.

These two events together are required to drive ER$^{i\text{-}splice}$. Indeed, we observed in **Fig. 1F**, and **see response to question°1 (page 5),** that SRSF1 is phosphorylated as soon as it is synthesised. We demonstrated also in **response to question°1 (see page 6)** that SRSF1 translation is p-eIF2a dependent since it can be rescued by ISRIB upon ER stress.

We propose the following model, where ER stress induces the translation of CLK1 and splicing factors such as SRSF1 to rewire splicing upon stress. **Please see the schematic below**:

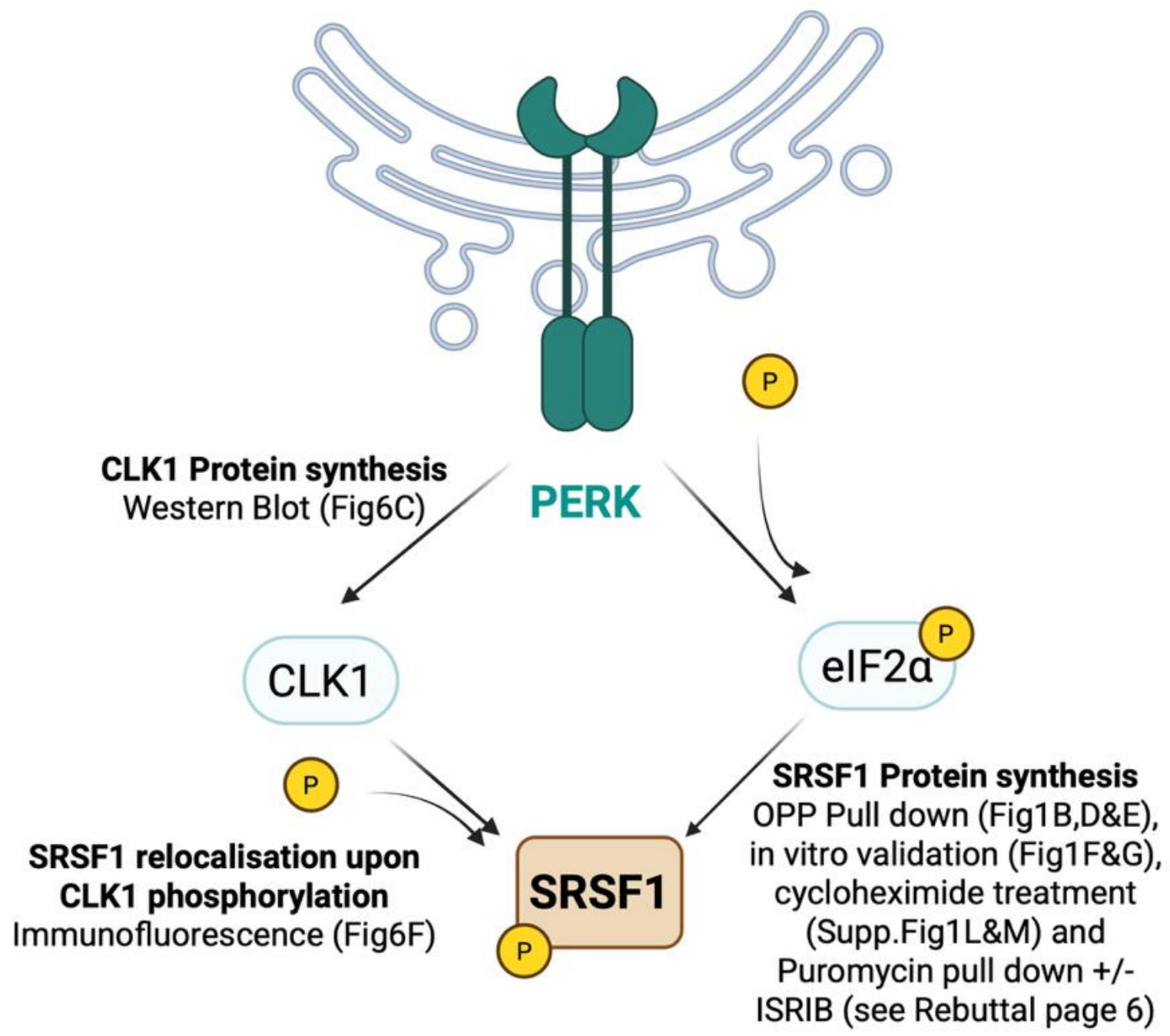

For completeness, we assessed the ER$^{i\text{-}splice}$ signature upon tunicamycin and ISRIB treatments. **Fig. A below** validates ISRIB-rescue protein translation upon ER stress. **Fig. B below** shows that ISRIB treatment prevents ER stress genes (*XBP1s* & *CHOP*) expression. Finally, **Fig. C below** shows that ISRIB treatment prevents the splicing of *PRR3*, *RBM39,* and *AP3S2*, while the rescue of *ARIH2* is not significant.

These results demonstrate that P-eiF2a is involved and necessary to the signalisation of this pathway.

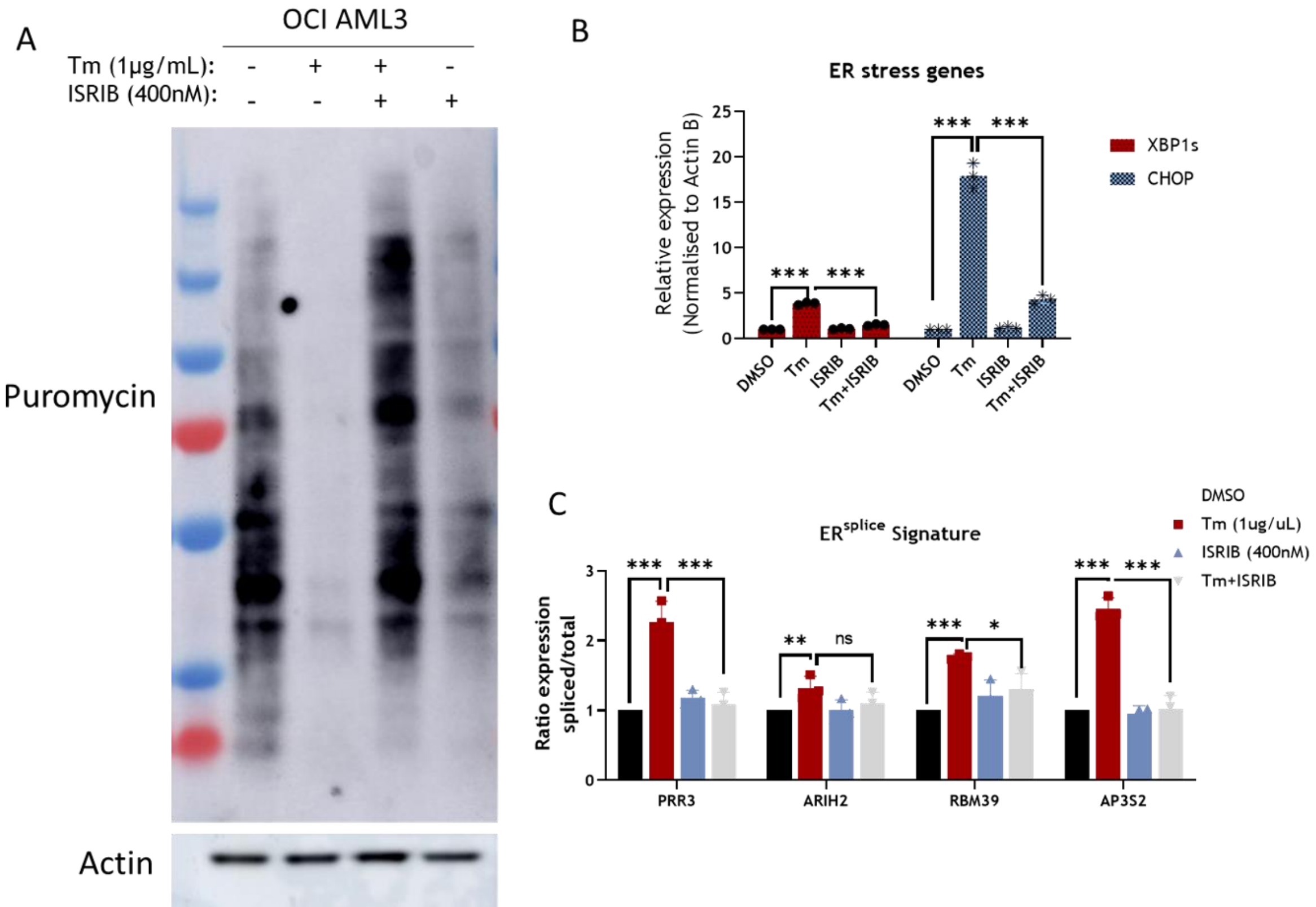

(A) Sunset Assay showing *de novo* peptides tagged with 2µM puromycin for 30min following 4H of tunicamycin treatment at 1µg/mL +/- ISRIB treatment at 400nM, OCI-AML3. Actin protein is used as a loading control. (B) Quantitative PCR analysis of *XBP1s* (Red panel) and *CHOP* (Blue panel) mRNA in OCI-AML3 cells treated with 1µg/mL tunicamycin for 4H +/- ISRIB at 400nM, in OCI-AML3. Data are normalized to Actin B (n=3). mean± S.D. ****p<0.005*. (C) Quantitative PCR of four alternative splicing events (*PRR3, ARIH2, RBM39* and *AP3S2*) occurring in OCI-AML3 treated with 1µg/mL tunicamycin for 4H +/- ISRIB at 400nM, in OCI-AML3. Data are normalized respectively to total *PRR3, ARIH2, RBM39* and *AP3S2* (n=3). mean± S.D. **p<0.05, **p<0.01, ***p<0.005.*

3. RNA-seq was performed at 4 h and 24 h of tunicamycin treatment. The 4 h time point is unlikely to capture effects of newly synthesized splicing factors, as translation, nuclear import, and engagement with nascent transcripts would require more time. The 24 h time point, while allowing de novo synthesis, carries a high risk of capturing indirect effects of prolonged UPR. A kinetic rationale should be added, particularly distinguishing fast kinase-mediated responses from slower translational ones.

We agree with the reviewer that, under basal conditions, the kinetics of protein synthesis, nuclear import, and functional engagement would likely preclude newly synthesized splicing factors from contributing to early (4h) effects.

However, under stress conditions, translational responses can occur much more rapidly due to the activation of the ER stress response. In this context, specific transcripts are selectively translated despite global translational repression. This is illustrated in **Supplementary Fig. 1I**, which shows a robust increase in ATF4 protein levels as early as 30 minutes following ER stress induction.

In our experimental design, *de novo* peptides were captured upon a 30min treatment of OPP, ensuring a cumulative view of newly synthesized proteins. To investigate adaptive pathways contributing to cellular recovery, we focused on the 4hours time point following stress induction. This corresponds to a time window at the end of the global inhibition phase (see western blot, **Fig. 5B**). In contrast, the 24hours time point was used to capture 'long lasting' splicing effects in order to obtain a robust signature transposable to different ER stress conditions. Overall, the risk of capturing indirect effects is mitigated by cross-referencing the findings between 4 and 24hours time points.

4. The authors overstate the concept of an "ER splice signature" (ER^{splice}). The term "signature" suggests strong reproducibility and directionality across cell lines, but: Out of >2000 events, only 8 are shared and PERK-responsive. Among four validated candidates, two appear to be stabilized NMD isoforms due to decreased translation rather than changes in splicing.

Indeed, the ER splice signature contains alternatively spliced (AS) and NMD sensitive isoforms. We thought that the term "splice" would adequately reflect both splicing and isoforms accumulation (for the NMD sensitive isoforms). To clarify the concept, we have now modified the acronym to **$ER^{i\text{-}splice}$** for ER induced splice/isoforms accumulation.

Overall, alternative splicing is extremely context and tissue-specific (Liang & Wilkins, bioRxiv, 2025). Here, we showed that the defined signature is ER stress-specific and not tissue-specific, as we validated the induction of the signature upon ER stress in 7 cell types in total, as presented figure **supplementary Fig. 3E&F** (OCI AML3, HEK293T, HepG2, HeLa, PC3, MCF7, and MSCs, which are primary human Mesenchymal stem cells isolated from three different healthy donors). Although this figure was mentioned in the manuscript, data were lost in the combined file, and we apologise for this. We have now corrected this and re-uploaded the correct file. Please see **supplementary Fig. 3E&F**.

Regarding the term "signature", we validated the analysis by using real-world data. We generated an ER stress score, a Z-score of gene expression in TCGA-cohorts of the nine ER stress genes identified in our PERK/tunicamycin dataset (*DNAJB9, ATF3, HSPA5, HERPUD1, EIF2AK3, HYOU1, DDIT3, STC2, ASNS*). Then, from The Cancer Genome Atlas Program (TCGA) we obtained the exon counts dataset for TCGA_PAAD (pancreatic cancer), TCGA_PRAD (prostate cancer), and TCGA_BRCA (breast cancer) from the Broad Institute Firehose (https://gdac.broadinstitute.org/) and extracted the RPKM values for our different exons of interest. We then created an $ER^{i\text{-}splice}$ signature based on the expression of seven alternative splicing events induced upon ER stress activation (*NUP62, RBM39, ARIH2, EIF4A2, NUDT2, AP3S2, ZFAS1*). A significant correlation could be observed between the ER score and the $ER^{i\text{-}splice}$ signature and was exacerbated in the top and bottom quartiles of each cohort (BRCA n=1090 samples; PAAD n=178 samples; PRAAD n=496 samples). These data validated our $ER^{i\text{-}splice}$ signature and confirmed the activation of a splicing program conserved across tissues and induced by the Unfolded Protein Response (**please see figure below**).

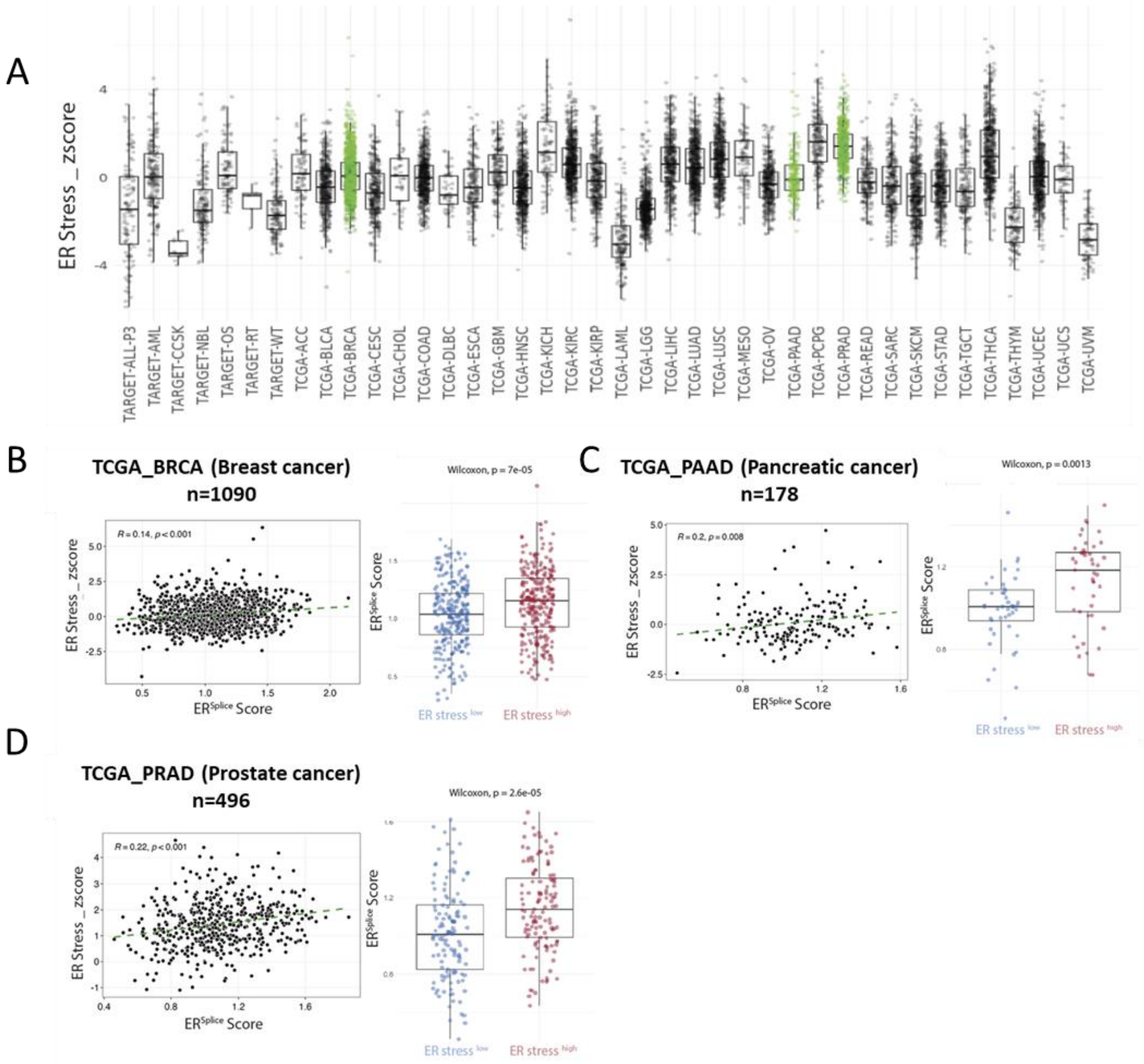

(A) Z-score calculated for the ER stress signature *(DNAJB9, ATF3, HSPA5, HERPUD1, EIF2AK3, HYOU1, DDIT3, STC2, ASNS)* per sample, across different TCGA cohorts. (B) Correlation plot of ER stress Z-score versus the ER^{splice} score in TCGA_BRCA cohort. n = 1,090 tumour samples. Pearson's r and p values are indicated on the graph. (Left panel). Boxplot representing the ER^{splice} score per ERstress group. Upper and lower quartiles were used to define ERstress high and low groups, n = 273 per group (Right panel). (C) Correlation plot of ER stress Z-score versus the ER^{splice} score in TCGA_PAAD cohort. n = 178 tumour samples. Pearson's r and p values are indicated on the graph. (Left panel). Boxplot representing the ER^{splice} score per ERstress group. Upper and lower quartiles were used to define ERstress high and low groups, n = 45 per group (Right panel). (D) Correlation plot of ER stress Z-score versus the ER^{splice} score in TCGA_PRAD cohort. n = 496 tumour samples. Pearson's r and p values are indicated on the graph. (Left panel). Boxplot representing the ER^{splice} score per ERstress group. Upper and lower quartiles were used to define ERstress high and low groups, n = 124 per group (Right panel).

Figure 5H shows dramatic cytoplasmic accumulation, but little nuclear change—raising questions about whether these are true splicing events or also stabilized NMD targets. The conclusions about a conserved ER splice program should be tempered.

Isoforms are shuttled out of the nucleus very quickly, within seconds (PMID: 22615357), and do not accumulate enough to show a comparable amplitude to the cytoplasmic accumulation.

In addition, we performed an RNAseq on OCI AML3 treated with tunicamycin +/- CLK1 inhibitor, TG003. We generated a splicing analysis with rMATS, focusing on skipping exons. We first represented the inclusion level for each ER$^{i\text{-splice}}$ transcripts in Tm versus DMSO (Tm) or in Tm+TG003 versus DMSO (Tm+TG003) (**see fig. A below**). We found that the three events (*AP3S2*, *ARIH2* et *RBM39*) were induced by tunicamycin but reversed by TG003, suggesting that those events rely on CLK1 activation upon ER stress. *AP3S2* splicing event could then results from both: an active splicing mechanism and a passive accumulation due to NMD inhibition.

Then, we extended the analysis to all common skipping exons affected by tunicamycin and by the combination of tunicamycin + TG003. We defined a list of 11219 exons with the same coordinate (**see fig. B below**). From this list, we found a total of 323 splicing events that are **exon exclusions** with ΔIncLevel ≥ 0.1, and 744 splicing events that are **exon inclusions** with ΔIncLevel ≤ -0.1 (**see fig. C below**). These results show that about 10% of the skipping exons are dependent of CLK1 upon ER stress, suggesting an active rewiring of splicing besides the stabilisation of NMD-sensitive transcripts.

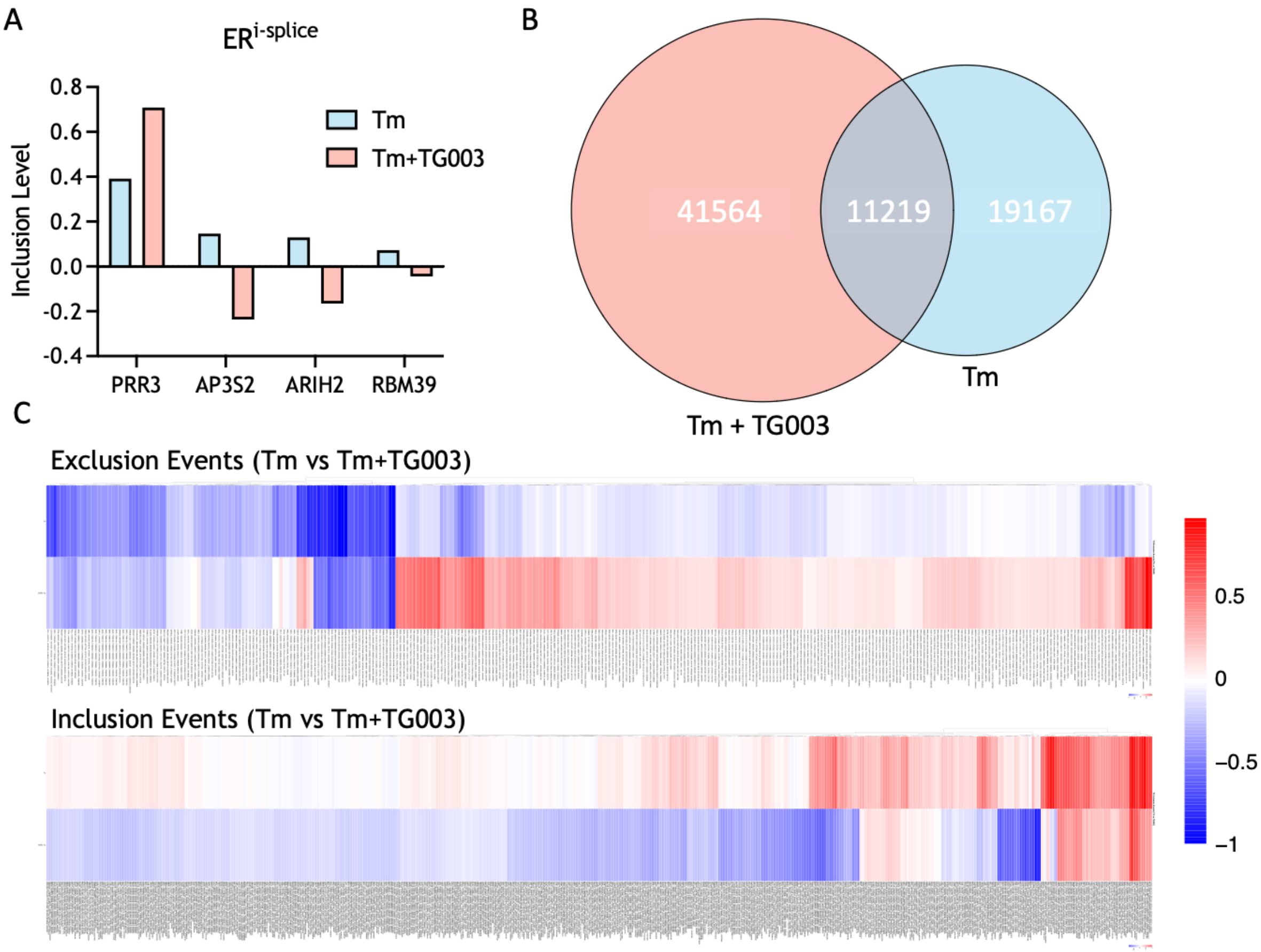

(A) Inclusion Level for each ER$^{i\text{-splice}}$ transcripts calculated by rMats in Tm versus DMSO (Tm) or in Tm+TG003 versus DMSO (Tm+TG003). RNAseq was performed in OCI AML3 treated with 1µg/mL tunicamycin (Tm) for 4H +/- CLK inhibitor (TG003). (B) Venn diagram showing the overlap in skipping exons (exact same coordinates) between Tm and Tm+TG003 treated cells. Both conditions were normalised to DMSO and events were selected with p-value < 0.05 . (C) Heatmaps depicting the exclusion events (top panel) or the inclusion events (bottom panel) from the overlap skipping exon list found in (B). Exclusion events were selected with a negative inclusion level and a TG003-induced increased inclusion ($\Delta \geq 0.1$), with a total of 323 splicing events affecting 205 genes.

5. The Puromycin pulldown + LC-MS/MS approach is elegant, but interpretation requires more nuance. If new synthesis of SR proteins is indeed detected: Are these changes robust across replicates?

We performed the OPP pull-down using agarose beads, which can introduce background due to their inherent nonspecific binding properties. To minimize this noise, we carried out the experiment using multiple OPP concentrations while keeping the amount of agarose beads strictly identical across all conditions. Under these conditions, an increase in peptide abundance across OPP concentrations indicates specific capture by the OPP rather than nonspecific binding to the agarose. This experimental design enabled the use of a Spearman correlation test to reliably distinguish newly synthesized peptides from the background.

The spearman analysis integrates the three biological replicates performed for each OPP concentration, thereby increasing the robustness and confidence of the peptide identification. In addition, we thoroughly validated our results for SRSF1 by independently confirming its stress-induced synthesis using a classical puromycin pull-down approach, as well as a cycloheximide time-course experiment under ER stress conditions (**Supplementary Fig. 1L&M**).

Could they reflect translational recovery or changes in global translation rather than specific induction? Supporting experiments (half-life measurements, isoform analyses) are needed.

We observed increased synthesis of splicing factors at 4 hours of ER stress, a time point at which global translation remains inhibited, as demonstrated by the SUNSET assay (**Fig. 5B**). This argues against a general recovery of translation as the primary explanation for our observations.

Furthermore, we show that this induction is dependent on eIF2α phosphorylation (**see response to Question 2, pages 7&8**), supporting a mechanism of selective translation within the stress response rather than global translational changes.

In addition, we have now strengthened our analysis by including SRSF1 half-life measurements under tunicamycin treatment (**Supplementary Fig. 1L&M**). We also performed isoform-level analyses (**see response to Question 1, page 5**), which do not support the hypothesis of induction of alternative, NMD-sensitive isoforms.

Together, these results support the conclusion that the observed increase in splicing factor expression reflects specific, stress-induced translational regulation rather than a nonspecific recovery of global protein synthesis.

6. The synergy in cell death is interesting, but the manuscript lacks mechanistic insight. Does the ER-induced splice program sensitize cells to RBM39 depletion? Are the affected exons enriched in pathways related to protein folding or apoptosis? More mechanistic depth is needed.

As shown in **Supplementary Fig. 2E**, protein folding, DNA repair, intracellular transport, and cell cycle are among the most enriched pathways affected by the ER-induced splice program in OCI-AML3 and HEK293T cells.

To refine this analysis, we performed a more focused analysis based on shared genes (**Fig. A below**) and then on common biological processes (**Fig. B below**) in OCI AML3 cells treated with tunicamycin. This analysis revealed both tissue-specific signalling pathways, such as retinoic acid

receptor signalling, and more general processes, including tRNA processing, cell cycle regulation, and DNA repair (**Fig. C below**). These pathways could either be promoted or impaired by the splicing events.

Indeed, the interpretation of these effects is limited by the use of short-read RNA sequencing, which does not allow precise resolution of isoform-specific functional outcomes.

Regarding our model of study, AML cells, we found it particularly relevant to focus on RBM39, which was found by the Ianis Aifantis Laboratory (PMID: 30799057) as one of the top essential RBPs in AML. Isoform-level analysis of RBM39 further illustrated the limitations of short-read sequencing, since there are at least 20 different RNA isoforms (**Supplementary Fig. 3C**), preventing accurate determination of which isoforms are differentially regulated.

We therefore examined the functional consequences at the protein level and found that RBM39 is upregulated upon ER stress (**Fig. 3E&F**), an effect that is reversed upon *SRSF1* knockdown (**Fig. 7C**). As RBM39 is documented in the literature to drive a pro-leukemic program (PMID: 30799057) and its inhibition has already been proposed as a therapeutic strategy, we wondered whether ER stress could potentiate this effect. Consistent with this, we observed a synergistic effect between tunicamycin and indisulam, an RBM39 inhibitor (**Fig. 7E&F**), highlighting the importance of RBM39 in stress resilience programs.

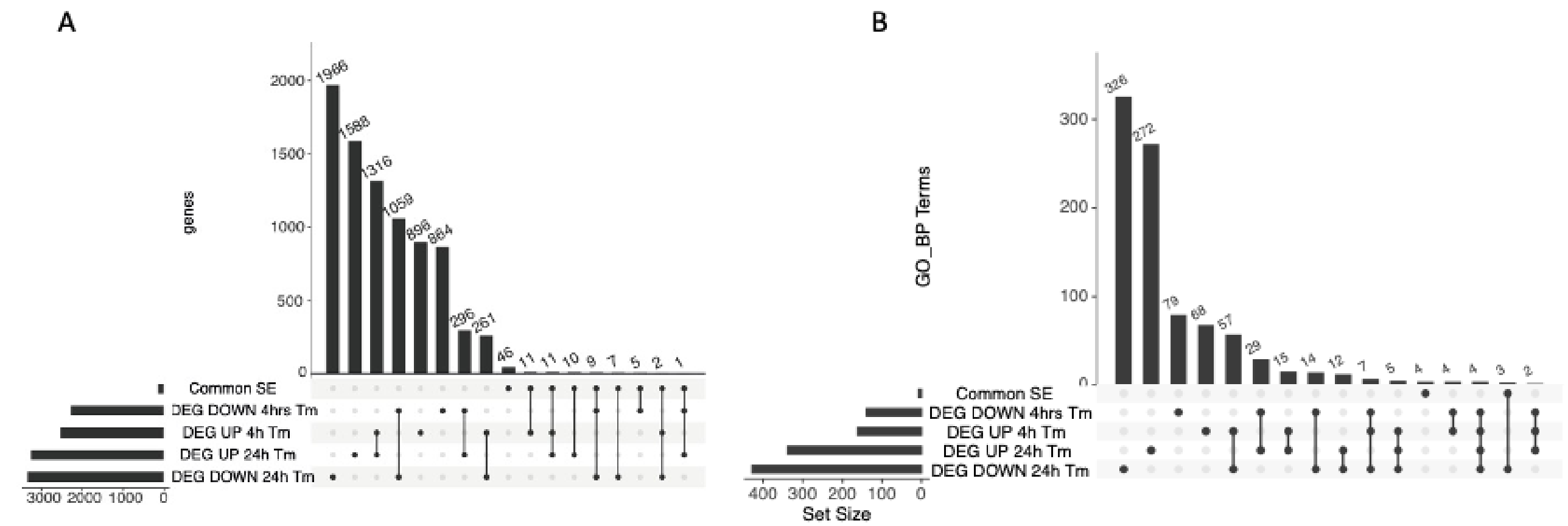

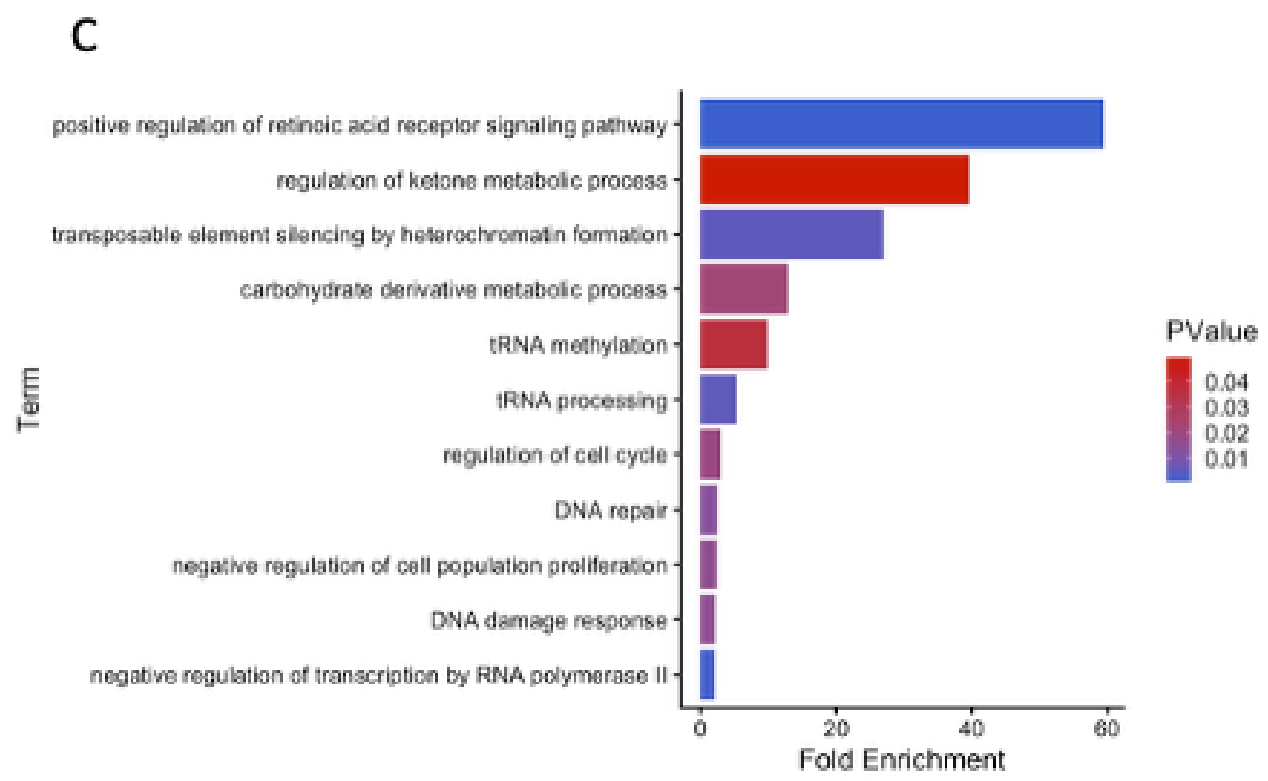

(A) UpSet plot depicting the overlap of genes undergoing differential gene expression (DEG) in OCI-AML3 treated with 1µg/mL of Tunicamycin for 4H or 24H, and common alternative splicing events detected upon ER stress (Common SE). (B) UpSet plot depicting the overlap of GO biological processes (GO_BP) enriched in OCI-AML3 treated with 1µg/mL of Tunicamycin for 4H or 24H, and GO_BP enriched among the common alternative splicing events detected upon ER stress (Common SE). (C) Gene Ontology analysis showing enriched biological processes' terms for the common alternative splicing events detected upon ER stress in OCI AML3. *pvalue* <0.05.

7. The discussion is too shallow and should include limitations of the study. For example, CLK1 activation during the UPR is shown, but its dependence on PERK is not established. While CLK1 likely contributes to a subset of splicing events, many observed transcript changes appear to stem from stabilization of NMD targets, not active alternative splicing. The authors should discuss whether the identified isoforms have functional relevance for ER stress survival or adaptation.

We thank the reviewer for their comment, and we now have discussed several limitations of the study in the discussion part (**lines 400-405**)

As shown in **Supplementary Fig. 2E**, protein folding, DNA repair, intracellular transport, and cell cycle are among the most enriched pathways (splicing events input) affected by the ER-induced splice program in OCI-AML3 and HEK293T cells.

8. Minor Comments

Figure 1 – The upper band in the SRSF1 western blot is unlikely to represent SRSF1. Authors should validate identity via siRNA knockdown or use of phospho-SRSF1–specific antibodies. The lower band's mobility shift at 24 h is also unexpected given CLK1-mediated hyperphosphorylation, which typically increases apparent molecular weight.

In order to validate that both bands are indeed SRSF1 proteins, we performed a knockdown of SRSF1 with siRNA, please see **Fig. 7C**. As shown on the figure, both bands are downregulated by the siRNA showing the specificity of these two bands.

Since SRSF1 autoregulates itself via 3'UTR splicing and NMD, the authors should examine potential isoform changes.

This is a relevant remark, we checked SRSF1 isoforms expression in our RNAseq experiment by running a package called IsoformSwitchAnalyzeR (ISAR). Results are displayed below. No changes in *SRSF1* isoforms were observed.

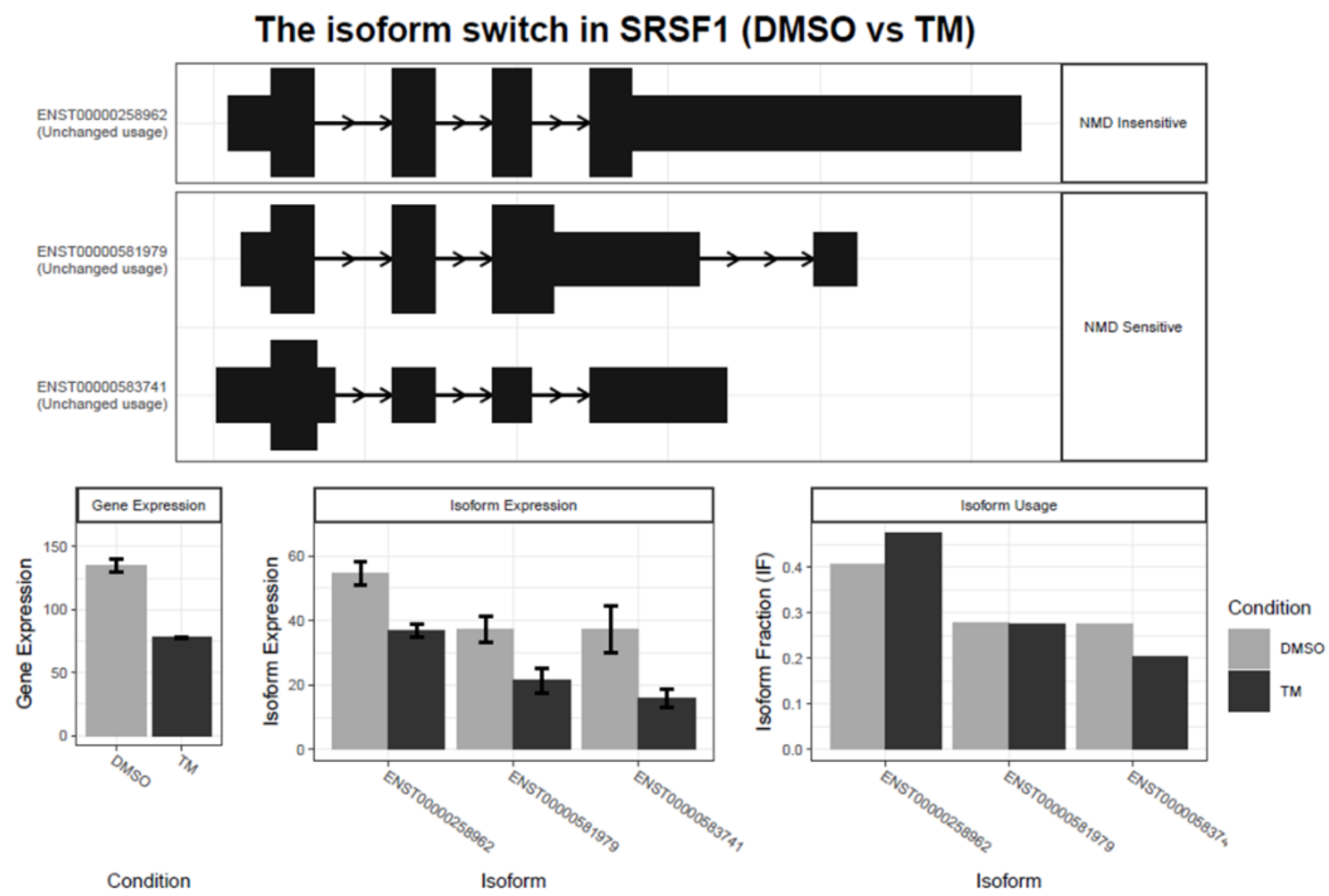

Graphs depicting the different SRSF1 isoforms found in OCI AML3 by using the IsoformSwitchAnalyzeR package (top panel). SRSF1 gene expression, isoforms expression and isoforms usage are represented by histograms in DMSO and tunicamycin (TM) treated OCI AML3 for 4H (bottom panel).

The statement "increase in skipping exon inclusion" is contradictory. Exon inclusion and exon skipping are mutually exclusive and should be described carefully.

We apologise for the lack of clarity in our original wording. The terminology used was based on the rMATS nomenclature, where "SE" (skipped exon) is a generic category that encompasses both exon inclusion and exon exclusion events.

We agree that the phrasing "increase in skipping exon inclusion" was confusing, as exon inclusion and exon skipping are mutually exclusive when described explicitly. To clarify, SE refers broadly to alternative splicing events affecting cassette exons, which can result in either increased inclusion or increased exclusion.

The text has now been revised (**line 162**) to clearly state "increase in exon inclusion."

RNA-binding proteins often contain regulatory exons producing NMD isoforms. Among the 33 shared events: How many correspond to NMD-linked exons? Are RBP autoregulatory circuits enriched?

This a very interesting point, while highly relevant, addressing the contribution of NMD-linked exons and autoregulatory RBP circuits is technically challenging given current bioinformatic tools limitations.

We used two complementary approaches to analyse alternative splicing: rMATS, which detects differential splicing events, and IsoformSwitchAnalyzeR (ISAR, based on DEXSeq), which focuses on differential exon usage to infer isoform-level changes. These tools rely on distinct

methodologies and are known to show limited overlap, as highlighted in recent comparative studies (PMID: 37020334; doi: 10.1101/2024.05.20.595048).

Given that our dataset is based on short-read RNA-seq, we considered rMATS to be more appropriate for robust identification of individual splicing events. We have also used rMATS in our previous work (*Mian & Philippe et al., Science Translational Medicine, 2023*), where it performed reliably for the identification of alternative splicing events. However, a key limitation of rMATS is that it does not reliably predict the functional consequences of these events, including NMD sensitivity. To address this, we complemented our analysis with ISAR (DEXSeq) to estimate the proportion of NMD-sensitive isoforms.

As expected, the overlap between ISAR differential isoform switches and rMATS differential alternative splicing events was limited (**please see below**).

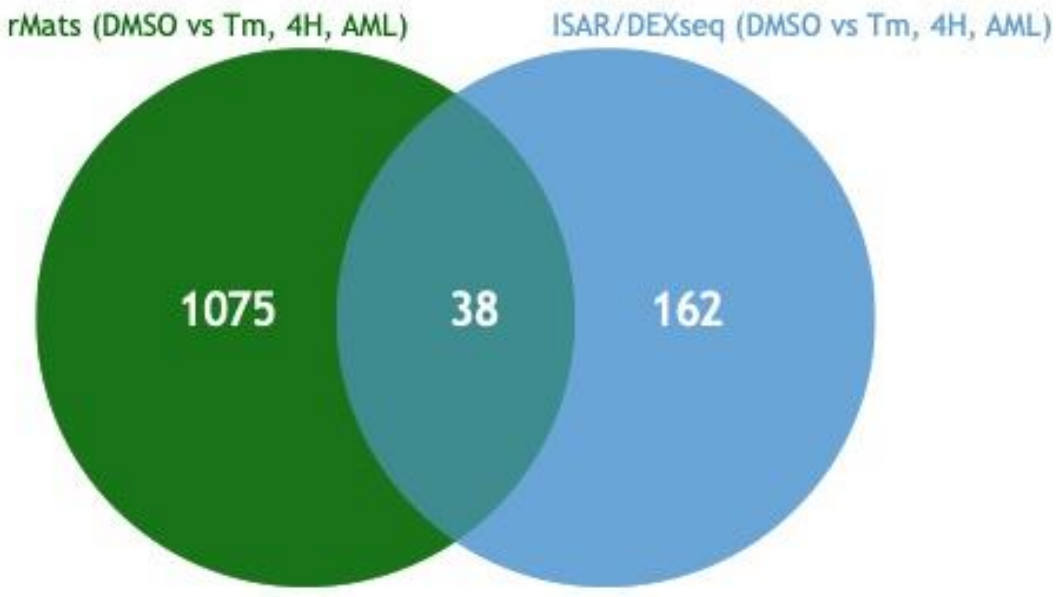

Venn diagram showing the overlap in genes commonly affected by skipping events detected by either rMats, with Inclusion Level>I0.2I, *pvalue*<0.05, or DEXseq (run with ISAR package), with dIF>I0.1I and *pvalue*<0.05.

As data test, we compared skipping exons in OCI-AML3 treated with either DMSO or tunicamycin for 4 hours. This dataset was the one used for the NMD prediction (**Fig. 5C**) in the manuscript, as this time point showed a great translation inhibition (**Fig. 5B**) that could increase the number of NMD-sensitive isoforms. We identified over 200 genes with differential isoform usage by ISAR. Of these, only 38 overlapped with the 1,113 genes identified by rMATS.

This level of concordance is consistent with previous reports (doi: 10.1101/2024.05.20.595048), which show that only a subset of events is commonly detected by both approaches, with many events uniquely identified by each method. For instance, it is mentioned in the manuscript doi: 10.1101/2024.05.20.595048: “Of those, 268 were called significant with both rMATS and DEXSeq, 3,236 were only called with rMATS, and 1,205 were only called with DEXSeq”), which is similar to what we observe.

Supplementary Figure 3 - Several panels appear to be missing and should be included for completeness.

We thank the reviewer for noticing. Please see below the full original figure that is now uploaded in the re-submitted version.

Supplementary Figure 3

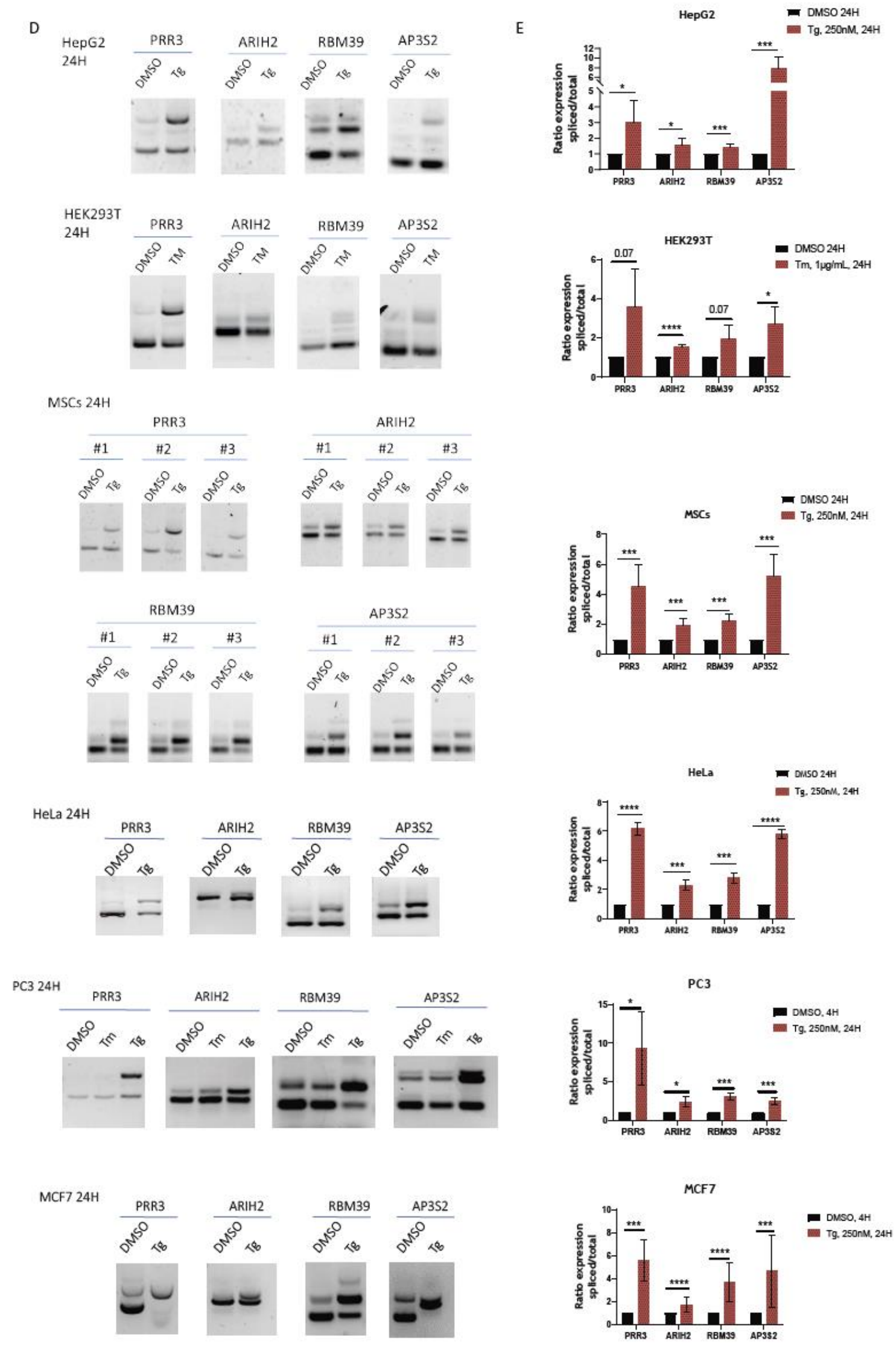

Figure 6 – The authors claim that ER stress releases SRSF1 from nuclear speckles and that CLK1 inhibition rescues this. Published studies (PMID: 39755675, 40716777, 36620874) show that other stresses (e.g., hypoxia) upregulate CLK1 and disperse SRSF5/6. These should be discussed to place findings into context.

This is now highlighted in the discussion **lines 363-381**.

As nuclear speckles are affected by different stresses, we tested a range of cellular stresses, including thermal stress, hypoxia, genotoxic stress, and metabolic stress. None of these conditions reproduced the alternative splicing events observed upon ER stress (**New figure Supp 4D-F and figure below**), indicating that the ER$^{i\text{-}splice}$ signature is not a generic consequence of cellular stress but is specific to ER stress.

In brief, we assessed the splicing of *PRR3*, *ARIH2*, *RBM39* and *AP3S2* in OCI-AML3 cells exposed to a range of stresses, including thermal stress (cold at 34°C or heat at 42°C), hypoxia (1% O_2), genotoxic stress (hydroxyurea, cisplatin, temozolomide), and metabolic stress (glutamine deprivation). Despite inducing cellular stress, none of these conditions reproduced the alternative splicing patterns observed upon ER stress induction (**see figure below**).

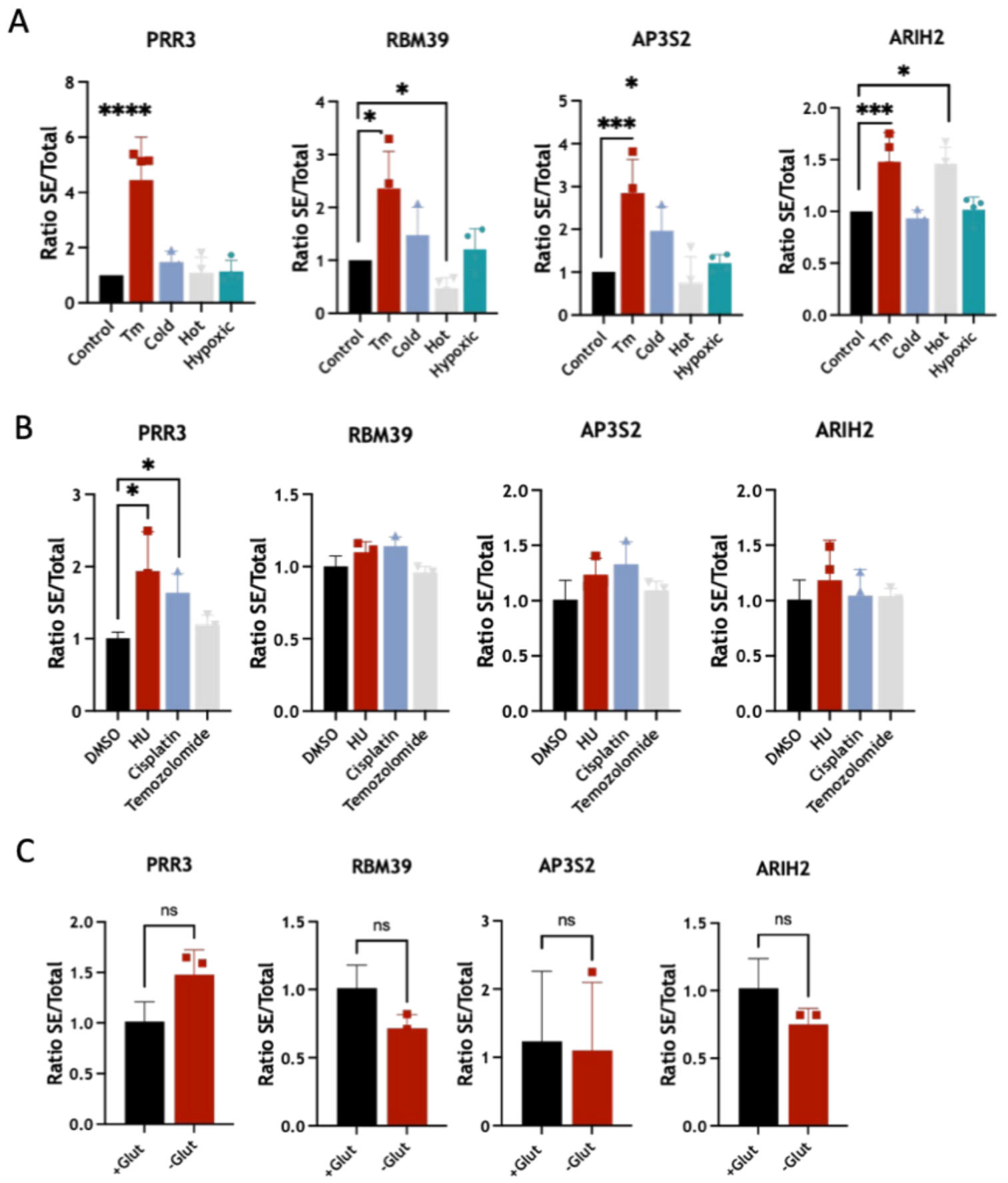

(A) Quantitative PCR of four alternative splicing events (*PRR3, ARIH2, RBM39 and AP3S2)* occurring in OCI-AML3 following ER stress (tunicamycin, 1µg/mL, 4H), Heat Shock either cold (34°C, 4h) or heat (42°C, 4h), and Hypoxia (1%O_2, 4h). Data are normalized respectively to total *PRR3, ARIH2, RBM39 and AP3S2* (n=3). mean± S.D. unpaired t.test. **p<0.05*, ****p<0.005*, *****p<0.001.* (B) Quantitative PCR of four alternative splicing events (*PRR3, ARIH2, RBM39 and AP3S2)* occurring in OCI-AML3 cells treated with genotoxic compounds (500 µM hydroxyurea, 3µM cisplatin or 50µM temozolomide) for 24H. Data are normalized respectively to total *PRR3, ARIH2, RBM39 and AP3S2* (n=3). mean± S.D. unpaired t.test. **p<0.05.* (C) Quantitative PCR of four alternative splicing events (*PRR3, ARIH2, RBM39 and AP3S2)* occurring in OCI-AML3 cells cultured +/- Glutamine for 4H. Data are normalized respectively to total *PRR3, ARIH2, RBM39 and AP3S2* (n=3). mean± S.D. **p<0.05*, ****p<0.005.*

Figure 6 - The microscopy images do not show canonical nuclear speckles. Instead, SRSF1-GFP accumulates in nucleoli, which is common upon protein overexpression. This undermines the claim that speckle disassembly is occurring. Endogenous staining or lower expression levels would be required.

We apologise if we were not clear, we did stain endogenous SRSF1 on the immunofluorescence pictures (**Fig. 6F**). We've now edited the text to clarify this, see **line 276**.

Figure 7 – The authors search for SRSF1 motifs in flanking exons, but SRSF1 generally binds within alternative exons to promote inclusion. Motif enrichment should be re-analyzed in the alternative exons themselves.

We thank the reviewer for their suggestions. We ran a motif enrichment analysis using the FIMO tool from the MEME suite on the list of alternative exons identified within the $ER^{i\text{-}splice}$ signature (33 exons in total).

The results are presented below. The pie chart represents the percentage of RBPs motifs found in the input sequences, either within the alternative exons (**left panel**) or in their flanking exons (**right panel**). We observed a higher enrichment of SRSF1 motifs in the flanking exons (26% of total detected motifs) compared to the alternative exons themselves (3% of total detected motifs).

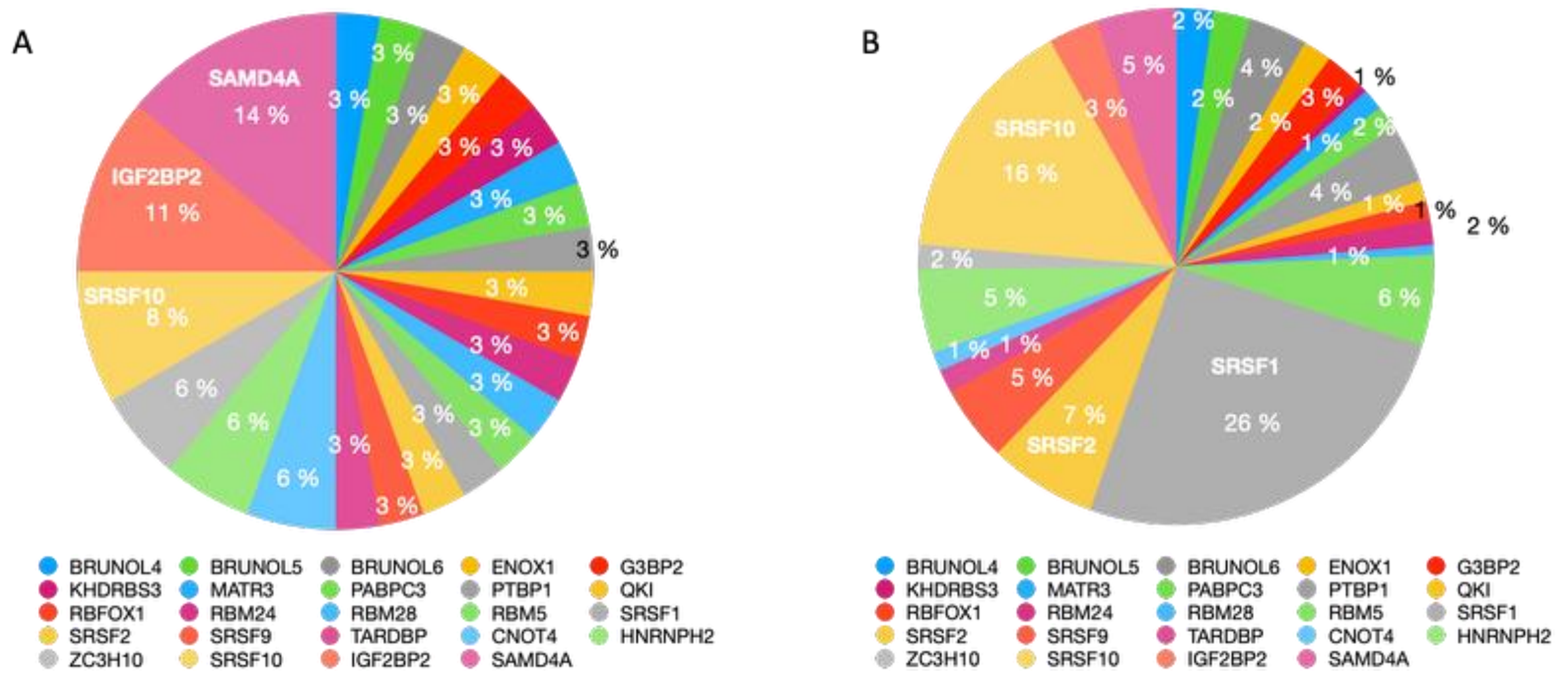

Chart pie displaying the percentage of RNA binding protein motifs on skipped exons sequences (A) or flanking exons (B) of the 33 common splicing events (OCI-AML3 and HEK293T cells) upon tunicamycin treatment at 4H and 24H. FIMO analysis was performed by providing by the Ray2013 motifs database (*Ray et al., Nature, 2013*).

Reviewer #3 (Remarks to the Author)

To the authors:

In the manuscript entitled „PERK orchestrates an endoplasmic reticulum stress alternative splicing program via CLK1/SRSF1", Philippe et al. use OPP labeling to identify newly synthesized proteins after triggering the unfolded protein response (UPR). Among these proteins the authors find numerous proteins involved in RNA processing and splicing. RNA sequencing after UPR activation reveals thousands of splicing pattern changes of which only a tiny fraction is shared between treatments and the cell lines employed. The authors claim that these changes to alternative splicing represent a signature specific to ER stress and demonstrate that they are mediated by activation of the eIF2alpha kinase PERK. While at least two of the UPR-induced mRNA isoforms (PRR3 and AP3S2 mRNA isoforms) represent NMD substrates that are stabilized upon activation of PERK, the other changes to alternative splicing are sensitive to inhibition of CLK1 or knockdown of SRSF1.

General Critique:

OPP-labelling and LC-MS/MS:

I commend the authors for using the OPP labelling approach to investigate protein translation upon activation of the UPR. However, I disagree with their statement that "due to the dynamic nature [...] and ribosome stalling, investigating translation upon stress has proven to be challenging" and that "therefore our understanding of [...] translatome rewiring upon stress remains limited" (l. 25-27). We and others have published comprehensive datasets that specifically address translation after pharmacological and genetic activation of the UPR employing different and complementary methodology under various, different conditions (e.g. Reich et al., 2020; Klann et al., 2020; Andreev et al., 2015 just to name a few). In this context, it would be very helpful if the authors would compare their dataset to the previously published ones, given that they use yet another methodological approach. What's the degree of overlap? What are the key differences? And if there are, what's the reason for those differences?

We thank the reviewer for this important comment and agree that several studies have already provided valuable insights into translational regulation during UPR activation using complementary approaches such as ribosome profiling and SILAC (e.g. Andreev et al., 2015; Klann et al., 2020; Reich et al., 2020). We apologise for the lack of nuance in our original statement.

To address the reviewer's point, we performed a systematic comparison of our OPP-based dataset with three published studies (**summarised in the table below**), taking into account the experimental context (cell type, stress inducer, duration, and methodology). Overall, we observed a moderate overlap in the identified proteins across datasets. However, this is likely explained by major differences in experimental design. Notably, most published studies were performed in adherent cell lines, whereas our data were generated in suspension cells. In addition, the nature and intensity of stress varied (e.g. tunicamycin at 1 µg/mL in our study versus 2.5 µg/mL, thapsigargin, or arsenite in others), as well as the analytical approaches used (predominantly ribosome profiling, with one SILAC-based study).

Importantly, the timing of analysis also differed substantially. While previous studies typically examined early (30 min–2.5 h) or later (6 h) time points, our analysis focused on a 4 h time point. Consistent with this, we observed that the degree of overlap increased with longer treatment durations. The largest overlap was found with the ribosome profiling dataset from astrocytoma cells treated with tunicamycin for 6 h (Reich et al., 2020), where 14 common proteins were identified, including SEC61B, SRP54, SYT1, and CHAC1, all associated with ER function and the UPR.

Together, these observations suggest that differences between datasets primarily reflect variations in cellular context, stress conditions, and temporal resolution rather than inconsistencies between methodologies. In this context, our OPP-based approach provides a complementary view of protein synthesis during the adaptive phase of the UPR.

Reference	PMID	Model, Cell Line	ER stress inducer	Time of treatment	Technique	Total hits	#in common with OPP	List in common with OPP
Reich et al., Nat. Comm., 2020	32522993	Astrocytoma, LN-308	Tunicamycin, 2,5ug/mL	2h	Riobosome Profiling	14 genes up	2	CHAC1, DYNLL1
Reich et al., Nat Comm., 2020	32522993	Astrocytoma, LN-308	Tunicamycin, 2,5ug/mL	6h	Riobosome Profiling	638 genes up	14	PGM3, ALDH1L2, BDH1, DDX49, EXOSC8, HEATR6, LSM8, MORF4L2, OGFOD1, RAB24, SEC61B, SRP54, SYT1, CHAC1
Reich et al., Nat Comm., 2020	32522993	Astrocytoma, LN-308	Thapsigargin, 200nM	2h	Riobosome Profiling	154 genes up	3	CHAC1, DYNLL1, NCAPH
Reich et al., Nat Comm., 2020	32522993	Astrocytoma, LN-308	Thapsigargin, 200nM	6h	Riobosome Profiling	636 genes up	10	PGM3, ALDH1L2, DDX49, HEATR6, MAPKAPK2, NDRG1, MRPS18A, SEC61B, SDHA, CHAC1
Klann et al., Mol. Cell, 2020	31812349	Cervical cancer, HeLa	Thapsigargin, 1uM	2.5h	SILAC (15-120min pulse)	6 peptides up	0	NA
Andreev et al., Elife, 2015	25621764	Embryonic kidney, HEK293T	Arsenite, 40uM	30min	Ribosome Profiling	35 genes up	0	NA
Philippe et al.,		OCI-AML3 Leukemic cells	Tunicamycin, 1ug/mL	4h	OPP-pull down LC-MS/MS	350 proteins up		

Regarding ribosome stalling upon activation of the UPR: we do not observe induction of specific ribosome pause sites in ribosome profiling data; also, there is no increase of disomes after RNaseI treatment which would be indicative of collisions due to stalling. Arguably eIF2alpha phosphorylation mediated by any of the eIF2alpha kinases (among them PERK -activated by the UPR- and GCN2 -activated e.g. by the ribotoxic stress response) is involved in resolving ribosome collisions by reducing ribosome loading on mRNAs presumably also helping to resolve stalling. In light of this, OPP tagging does not offer any obvious advantages over ribosome profiling (in particular when those data are additionally validated by changes in steady state protein levels detected shotgun MS). While I very much appreciate the alternative methodology to monitor translation output under stress, the authors should down tone their claims about the advantages of OPP tagging.

We thank the reviewer for pointing out this specific aspect of the demonstration. Indeed, the presence of stalled ribosomes in stress granules is a debated topic as previous papers demonstrated opposite results (*Wu et al, Science Advances, 2025*). The sentence has been removed from the abstract to avoid any confusion.

Regarding the use of ribosome profiling to study protein synthesis upon stress, stalled ribosomes are not the only bias we could identify. Translation changes upon stress can be rapid, for instance ATF4 is translated within the first 30min following stress induction (**see Supp Fig. 1I**). However, time resolution of ribosome profiling is really dependent of the experimental design, as it captures snapshot at specific time rather than monitoring within a time frame what is translated. The difference of approach and results is illustrated by the modest correlation (r = 0.37–0.43) that was previously assessed between ribosome profiling and other methods, including pSILAC and PUNCH-P, respectively (*Aviner et al., Genes Dev, 2013*).

To push further the comparison and see if these results could have been obtained with ribosome profiling, we generated, in collaboration with Dr Marcos Morgan's lab (National Institute of Environmental Health Sciences (NIEHS), North Carolina, US), a Riboseq analysis of DMSO and Tunicamycin OCI-AML3 cells, treated for 4H with 1µg/mL tunicamycin. Forty-one genes, Log2FC ≥ I0.2I, *p.value adjusted* ≤0.05 were captured (**Fig. A below**). Among these 41 genes, 2 were common to the OPP pull-down list (**Fig. B below**), including CHAC1, one of the top proteins captured (**Fig. 1B**) and whose gene expression showed the highest fold change in our RNAseq

(**Fig. 2E**). Among the 4 GO biological processes (GO_BP) enriched, we observed the response to unfolded protein (**Fig. C below**). Thus, the enrichment in ER stress-related terms validates the Ribo-Seq approach, however we did not capture splicing factors in the 41 genes. As a comparison, Reich et al. (PMID: 32522993) capture by Ribosome profiling, at 2 hours, about 13 transcripts differentially regulated vs 260 differentially regulated transcripts at 6 hours, and only 7 of these transcripts are captured by their proteomic experiment.

Contrary to the OPP-LC-MS/MS approach where proteins/peptides are captured over a period of 30 minutes, Ribo-Seq represents a snapshot of transcripts trapped in Ribosomes at a given time. In order to validate the results obtained with OPP, we focused on SRSF1 as a proof of concept. In addition, we thoroughly validated our results for SRSF1 by independently confirming its stress-induced synthesis using a classical puromycin pull-down approach, as well as a cycloheximide time-course experiment under ER stress conditions (**See Reviewer 2 -Question 1, page 4-6**).

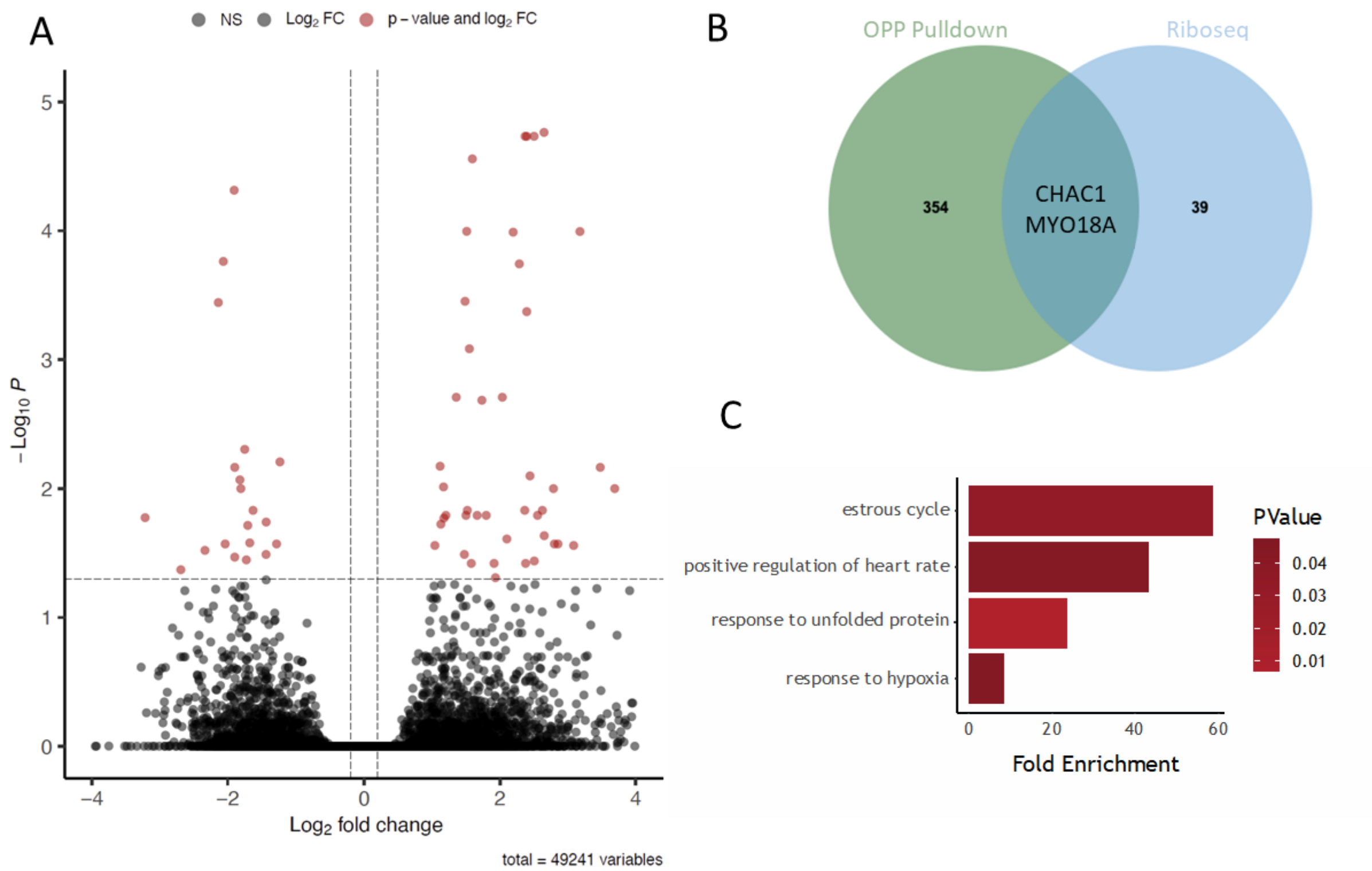

(A) Volcano plot depicting the statistical significance and Log2 fold change in translation efficiency between DMSO and Tunicamycin treated OCI-AML3 cells. Cells were treated for 4H with 1µg/mL tunicamycin. (n=3). Log2FC ≥ I0.2I, p.value.adjusted ≤0.05. (B) Venn diagram showing the overlap in proteins captured by OPP Pulldown and protein equivalent transcripts captured by Riboseq. (C) Gene Ontology analysis showing enriched biological processes' terms for the Riboseq-captured transcripts. pvalue <0.05.

Even after scrutinizing the manuscript, it still remains unclear to me how the OPP experiment was conducted. In the main body of the manuscript and the Materials and Methods section, the authors do not mention any negative controls or disclose how they bioinformatically generated the list of 350 proteins that "significantly correlated [...] with OPP concentration upon ER stress".

Why did the authors use correlation with OPP concentration as a criterium in the first place? Even proteins whose translation is attenuated upon stress should exhibit this correlation.

Finally, induction of the UPR does not entirely shut-off translation and most mRNAs in the cell still produce protein, albeit at a reduced rate. These should also be detectable among the "bona fide proteins de novo synthesized upon endoplasmic reticulum stress" (l. 85). Why do the authors then come up with a list comprising only 350 proteins? Surely, some bioinformatic sorting/scoring has been performed which is not disclosed in the manuscript.

We agree with the reviewer that more details were needed on the OPP pull-down analysis. We performed the OPP pull-down using agarose beads, which can introduce background due to their inherent nonspecific binding properties. To minimize this noise, we carried out the experiment using multiple OPP concentrations while keeping the amount of agarose beads strictly identical across all conditions. Under these conditions, an increase in peptide abundance across OPP concentrations indicates specific capture by the OPP rather than nonspecific binding to the agarose. This experimental design enabled the use of a Spearman correlation test to reliably distinguish newly synthesized peptides from the background. The Spearman analysis integrates the three biological replicates performed for each probe concentration, thereby increasing the robustness and confidence of the peptide identification. More details have now been added, highlighted in the materials and methods section.

Of interest are rather the **changes in translation rate** upon induction of the UPR and the **overall translation rate** during the UPR, which need to be determined relative to control cells. This important control – a control treatment – is mentioned only in the legend to figure S1G: log2 fold change of TM treatment over vehicle (DMSO). Unfortunately, Table 1 lacks all the relevant information (such as e.g. FC relative to control, Z-score etc.) and only provides the identity of the 350 genes.

We thank the reviewer for this constructive remark. For the 350 selected proteins, we have now added a column corresponding to the log2 Fold change of Tm treatment over vehicle for the OPP=10uM condition (**see supplementary table 1**).

And why don't they identify ATF4 among the highly translated proteins? They comment on this in l. 104-105. However, without a proper description of how their data was derived, I cannot follow this reasoning.

The translation of ATF4 is occurring quickly upon ER stress induction, within the first 30 minutes (**see western blot, Supp fig. 1I**). After 4 hours, the ATF4 protein level has reached a plateau of protein expression (see western blot below), and translation is slowing down. Despite this, we can still capture ATF4 in the OPP pull-down experiment upon tunicamycin (**Supp Fig. 1H**). For the convenience of the reviewer, we generated another WB following the kinetics of expression and decay of ATF4 over time, in OCI AML3 cells. **Please see below**.

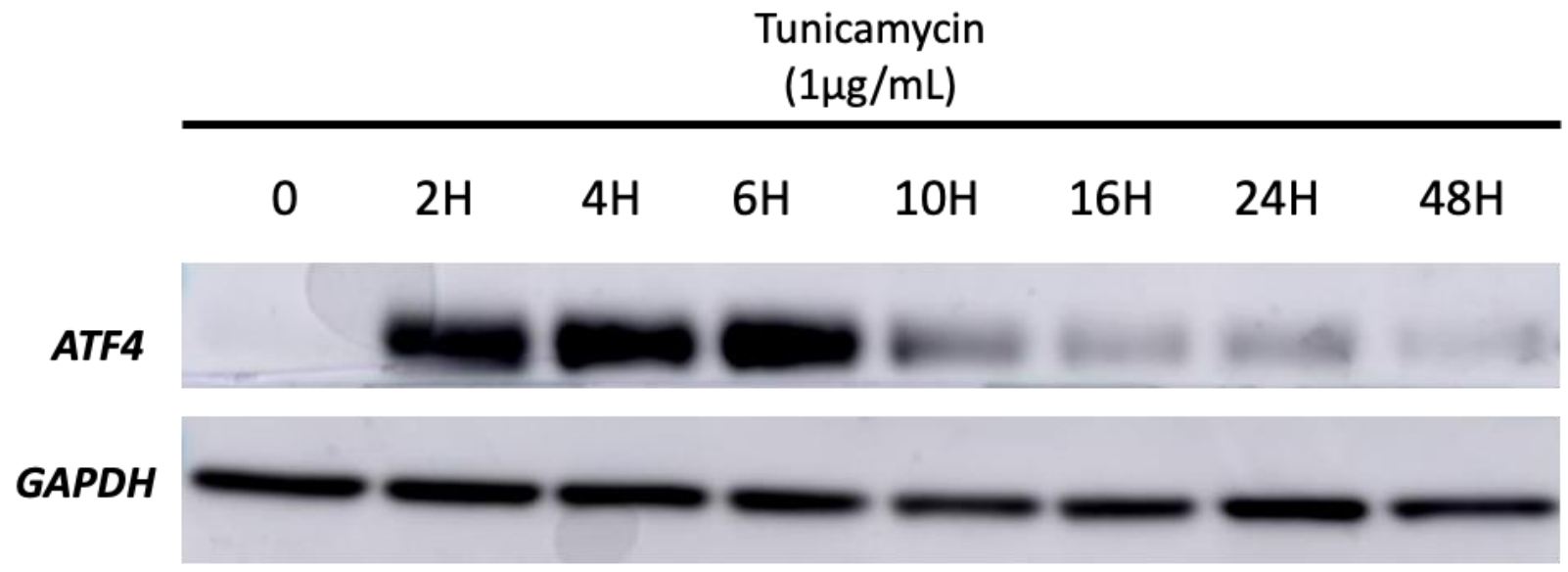

In summary I am truly puzzled by the experiment, most likely because important information is lacking from the manuscript.

We thank the reviewer for their feedback. Further details have been added to the manuscript according to reviewers' comments. We kept track of all editing and highlighted changes.

Splicing signature and activation of CLK1:

The changes to AS observed by the authors in two different cell lines and at two different time points after induction of the UPR are hardly similar – only less than 10% (of each individual treatment) are shared, a rather poor correlation and far from convincing. Would this not be in the range of a spurious overlap between two completely unrelated treatments? The authors should compare to another type of stress (genotoxic stress, hypoxia, starvation, heat shock, or else) to show that the changes observed upon TM treatment are significant.

We acknowledge the reviewer's remark, and to address it, we performed different types of stress in the OCI AML3 cells model. Briefly, we exposed the cells to heat shock either cold (34°C, 4h) or heat (42°C, 4h), Hypoxia (1%O2, 4h), a panel of genotoxic drugs (Hydroxyurea, Cisplatin, and Temozolomide), and glutamine starvation (**please see figure below and new supplementary Fig. 4D-F**). We then measured by qPCR the expression level of the 4 shortlisted isoforms over the total expression of the gene. Across all stresses, we find that only a treatment with tunicamycin can fully recapitulate the $ER^{i\text{-}splice}$ events we described. Overall, alternative splicing is extremely context and tissue-specific (Liang & Wilkins, bioRxiv, 2025). Here, we showed that the defined signature is ER stress-specific and not tissue-specific, as we validated the induction of the signature upon ER stress in 7 cell types in total (OCI AML3, HEK293T, HepG2, HeLa, PC3, MCF7, and MSCs from three independent healthy donors). Although this figure was mentioned in the manuscript, some of the data were lost during the generation of the combined PDF file; we apologise for this. We have now re-uploaded the original full figure, see **supplementary figure 3E&F**.

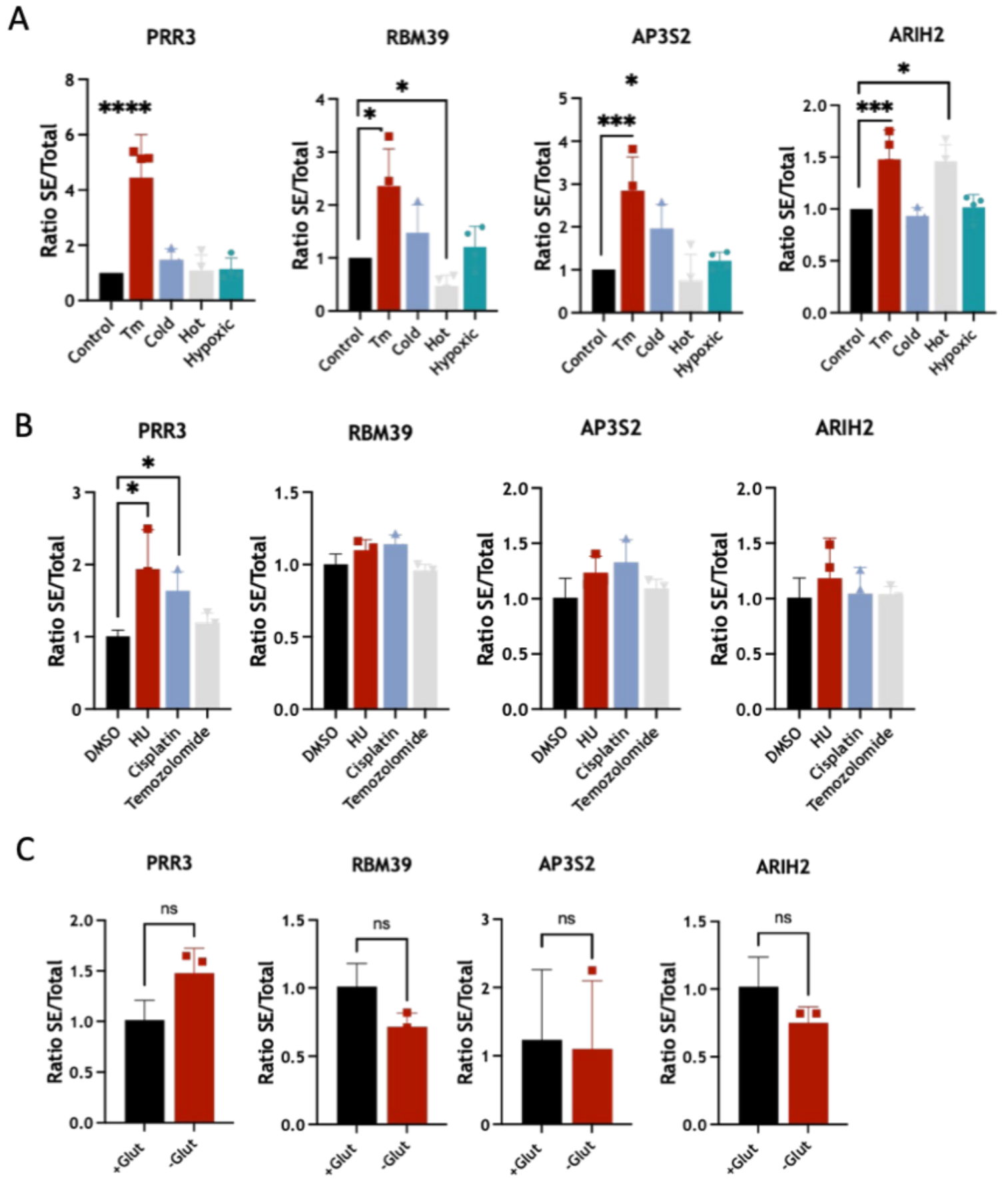

(A) Quantitative PCR of four alternative splicing events (*PRR3, ARIH2, RBM39 and AP3S2)* occurring in OCI-AML3 following ER stress (tunicamycin, 1µg/mL, 4H), Heat Shock either cold (34°C, 4h) or heat (42°C, 4h), and Hypoxia (1%O2, 4h). Data are normalized respectively to total *PRR3, ARIH2, RBM39 and AP3S2* (n=3). mean± S.D. unpaired t.test. **p<0.05*, ****p<0.005*, *****p<0.001.* (B) Quantitative PCR of four alternative splicing events (*PRR3, ARIH2, RBM39 and AP3S2)* occurring in OCI-AML3 cells treated with genotoxic compounds (500 µM hydroxyurea, 3µM cisplatin or 50µM temozolomide) for 24H. Data are normalized respectively to total *PRR3, ARIH2, RBM39 and AP3S2* (n=3). mean± S.D. unpaired t.test. **p<0.05.* (C) Quantitative PCR of four alternative splicing events (*PRR3, ARIH2, RBM39 and AP3S2)* occurring in OCI-AML3 cells cultured +/- Glutamin for 4H. Data are normalized respectively to total *PRR3, ARIH2, RBM39 and AP3S2* (n=3). mean± S.D. **p<0.05*, ****p<0.005.*

I very much appreciate the efforts the authors made to demonstrate how the UPR (via PERK) impacts on alternative splicing. However, it remains unclear, how PERK activity results in activation of CLK1. Is it a direct downstream target? Likely not given that SR protein phosphorylation peaks at 4h after induction of the UPR and not earlier. Does activation of another eIF2Alpha kinase (e.g. GCN2, HRI, PKR, or MARK2) produce a similar splicing pattern? Does treatment with e.g. salubrinal recapitulate these changes and can they be blunted by ISRIB?

In other words, are those changes to AS mediated by eIF2alpha phosphorylation, or driven by another PERK downstream target such as Nrf2?

This would also address the question whether the particular changes to AS are truly ER stress specific as suggested by the authors (named ER^{splice}), or shared with other pathways of the ISR.

We thank the reviewer for this relevant comment. To determine if the phosphorylation of eIF2a is necessary to observe the $ER^{i\text{-}splice}$ signature, we used ISRIB that increases the guanine exchange factor, eIF2B and consequently rescues protein synthesis.

We confirm the protein synthesis rescue by sunset assay (**Fig. A below**). We also observed as expected that ER stress is relieved as indicated by the decreased expression of XBP1 and CHOP upon ISRIB treatment in ER stress condition (**Fig. B below**). Finally, the $ER^{i\text{-}splice}$ signature was fully rescued by the addition of ISRIB upon stress (**Fig. C below**). These results show that the phosphorylation is necessary for the induction of $ER^{i\text{-}splice}$.

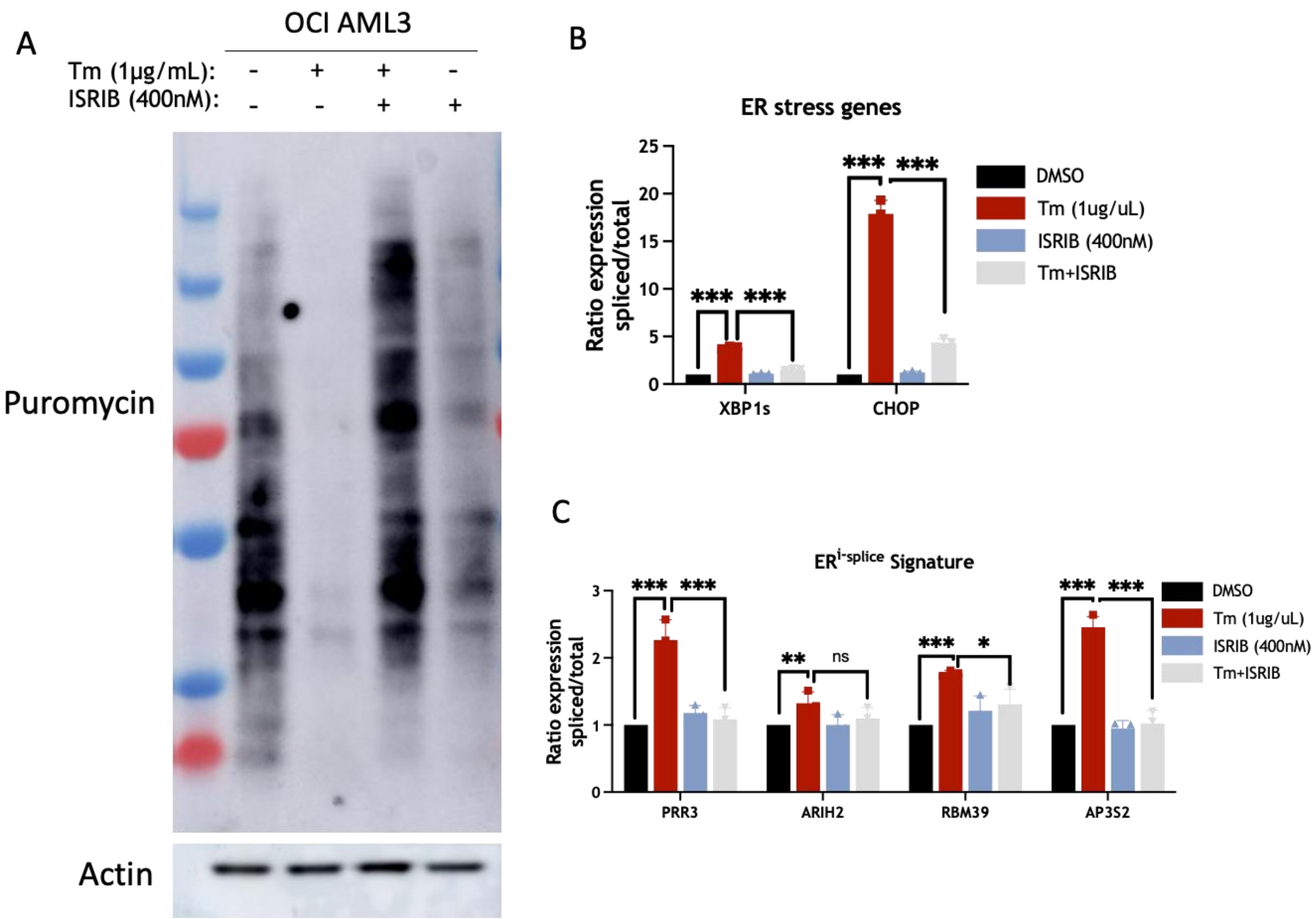

(A) Sunset Assay showing *de novo* peptides tagged with 2µM puromycin for 30min following 4H of tunicamycin treatment at 1µg/mL +/- ISRIB treatment at 400nM, OCI-AML3. Actin protein is used as a loading control. (B) Quantitative PCR analysis of *XBP1s* (Red panel) and *CHOP* (Blue panel) mRNA in OCI-AML3 cells treated with 1µg/mL tunicamycin for 4H +/- ISRIB at 400nM, in OCI-AML3. Data are normalized to Actin B (n=3). mean± S.D. ****p<0.005*. (C) Quantitative PCR of four alternative splicing events (*PRR3, ARIH2, RBM39* and *AP3S2*) occurring in OCI-AML3 treated with 1µg/mL tunicamycin for 4H +/- ISRIB at 400nM, in OCI-AML3. Data are normalized respectively to total *PRR3, ARIH2, RBM39* and *AP3S2* (n=3). mean± S.D. **p<0.05, **p<0.01, ***p<0.005.*

Regarding the question of the NRF2 pathway involvement in this mechanism, we performed a knock down experiment with a siNRF2 in OCI AML3 cells. Although we found an efficient KD of 70% of NRF2 (**Fig. A below**), we did not observe any rescue of the splicing events induced by ER stress (**Fig. B below**), which suggests that NRF2 is not involved in this response.

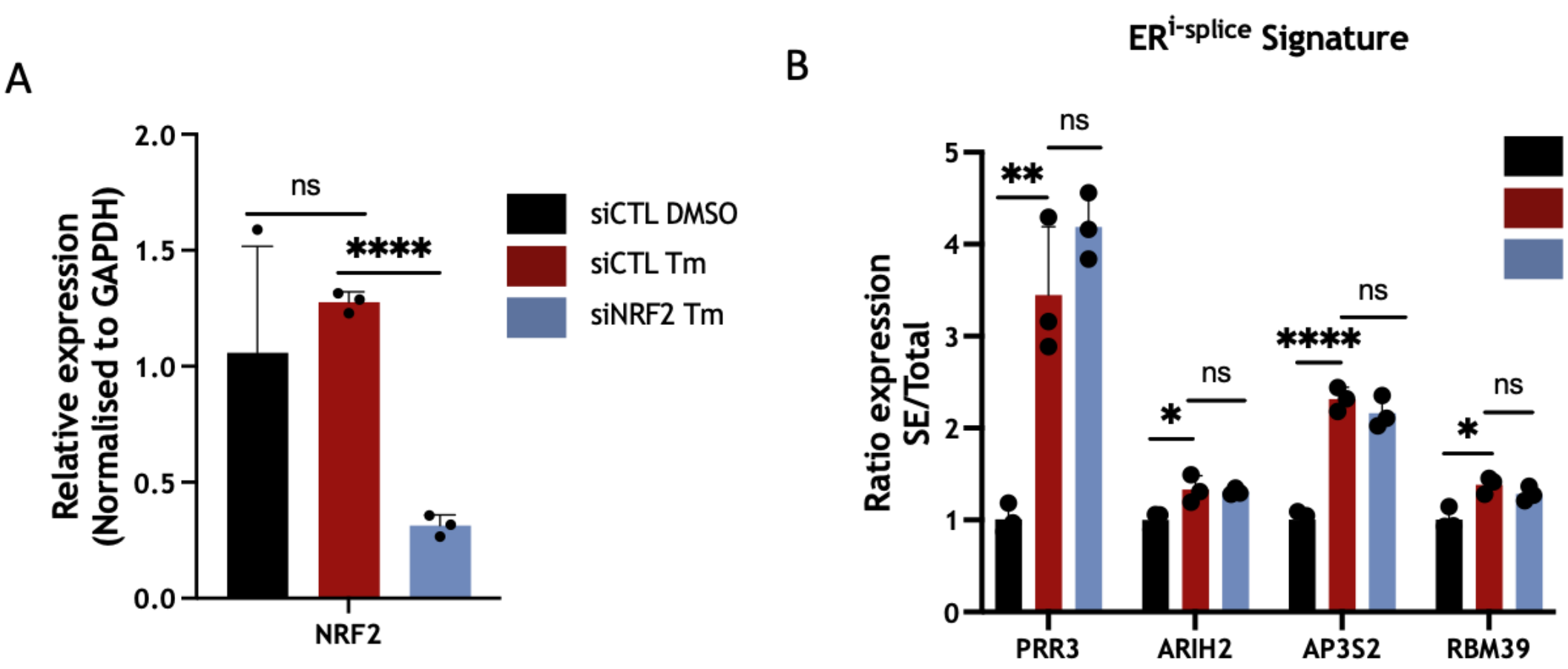

(A) Quantitative PCR analysis of *NRF2* in OCI AML3 cells that have been transfected with either siRNA control (CTL), or siRNA NRF2 for 48H, and then treated with 1µg/mL tunicamycin for 4H. Data are normalized to GAPDH (n=3). mean± S.D. * *p<0.05,* ** *p<0.01,* ****p<0.005,* *****p<0.001.* (B) Quantitative PCR of four alternative splicing events (*PRR3, ARIH2, RBM39 and AP3S2)* occurring in OCI AML3 cells treated with 1µg/mL tunicamycin for 4H and transfected with either siRNA control (CTL), or siRNA NRF2 for 48H. Data are normalized respectively to total *PRR3, ARIH2, RBM39 and AP3S2* (n=4). mean± S.D. **p<0.05*, ***p<0.01*, ****p<0.005*, *****p<0.001.*

Regarding the question whether CLK1 is a direct target of PERK, "Is it a direct downstream target". As the reviewer we think that it is unlikely, for instance it was not identified in our phosphoproteomic analysis performed upon inhibition of PERK kinase activity.

Does the LC-MS/MS analysis capture **peptides indicative of the changes to AS**? If so, what's the fraction of the protein isoform produced by alternative splicing relative to the overall amount of the respective protein (could also be addressed by WB analysis)? Also, the authors have not addressed the effect that the changes to AS might have on the function of the respective encoded proteins (potential LOF, GOF, competitive inhibitor etc.).

This is an interesting question. We ran a PIT analysis (Proteomics Informed by Transcriptomics PMID: 23142869). In brief, proteomic MS/MS spectra are searched against open reading frames derived from de novo assembled transcripts using SUPPA2 (instead of DEXseq or rMATS). The use of this pipeline on our OPP data is limited, as usually it would require proteomic samples labelled with TMT to normalise peptides. However, it was sufficient to identify 4 peptides harbouring the splicing sequence identified by transcriptomic, including RBM39 and EIFAE2 (**see figure below**). Although the approach is limited, it shows that some of the splicing events identified lead to stable translation of proteins.

		Intensity					
Gene	Peptide	DMSO rep1	DMSO rep2	DMSO rep3	Tm rep1	Tm rep2	Tm rep3
RBM39	ADDIDIEAMLEAPYKKDENK	0	0	0	0	0	95884
SHMT2	AFPMPGFDEH	0	0	0	2261900	2163200	1236100
WARS1	AGNASKDEIDSAVK	0	0	2176900	2311900	3214800	0
EIF4A2	DFTVSALHGDMDQKER	0	0	0	0	379050	669700

Peptide level evidence for 4 unique genes obtained from the analysis on the 6 LC-MS/MS OCI AML3 samples (3 DMSO replicates and 3 Tunicamycin treated replicates) by MaxQuant.

Regarding the functional consequences of AS, one of the limitations of short-read RNAseq compared to long-read sequencing technologies is that we cannot determine among the different isoforms harbouring the same exons which specific isoform is captured. Functional assays on a single event must be performed in order to understand the consequences of splicing at the protein level. We performed a western blot on the four targets; however, only AP3S2 and RBM39 (**Fig. 3E&F**) were detected at the protein level, although we cannot exclude that the antibodies for ARIH2 and PRR3 were not working.

We focused on the RNA signature as a readout of the ER stress splicing activity rather than the actual function of the final protein. Although for completeness and as a pharmacological inhibitor was available for RBM39, we demonstrated that the inhibition of RBM39 synergizes with ER stress induction to kill the cells (**Fig. 7E&F**). This is very much relevant and interesting as RBM39 is an RNA-binding protein involved in splicing regulation.

As mentioned by the reviewer, a thorough investigation of these spliced isoforms is required to fully understand their biological significance in the context of stressed cells and should be the focus of the following investigations.

Biological Importance of the observed alternative splicing (AS) events:

Unfortunately, also the biological importance of the observed changes to AS remains vague. Increased apoptosis upon induction of the UPR in combination with SRSF1 knockdown or tinkering with RBM39 does not convincingly demonstrate that both are causally related – in particular since negative controls are lacking entirely. Pushing cells further to the limit by pharmacological activation of the UPR typically sensitizes them to various other types of challenges and increased apoptosis. The observed effect upon combined treatment therefore does not necessarily reflect a direct interaction. Hence, I am not convinced that the authors have discovered an "new pro-survival facet of ER stress" (l. 35). More data and refined experimentation are needed to mount a convincing case.

Pro-survival was removed from the sentence **line 41**.

All appropriate controls, including vehicle and siControl conditions, are included and shown in the relevant figures. We note that the experiment presented is a **synergy analysis**, specifically designed to distinguish between additive and synergistic effects. The increased apoptosis observed upon combined ER stress and RBM39 inhibition is not simply cumulative but exceeds the expected additive effect.

While it is well established that ER stress can sensitize cells to additional perturbations, this does not account for the synergistic effect observed here. The purpose of our approach was precisely to control for this possibility.

Minor points:

l. 94: typo- should read cycloheximide instead of cycloheximide

Thank you, it has been modified. **Line 101 now**.

l. 134: the event is called exon skipping

We apologise for the lack of clarity. The terminology used was based on the rMATS nomenclature, where "SE" (skipped exon) is a generic category that encompasses both exon inclusion and exon exclusion events.

l. 136: what's an inclusion level of -0.66%; percentage of inclusion or fold change would be more informative

The inclusion level of -0.66% means with rMATS that 66% of the total RNA harbours an exclusion of the given exon. This terminology is proper to rMATS while other packages of splicing analysis such SUPPA2 or DEXseq will usually use percentile of splicing (PSI).

l. 144: a venn diagram is not an analysis but rather a way to depict data

"analysis" has been removed. **Line 156**

l. 147 & 184: what do the authors mean by "identical coordinates"? I can only assume that it is the exact same change to AS?

Yes, sorry if it was not clear.

l. 150 & 151: "skipping exon inclusion" and "increase in skipping exon inclusion" should be rephrased

To improve clarity, we modified the text for "increase in exon inclusion", see **line 162**

l. 197ff: what the authors observe are transcriptomic changes; hence, it is not translation reprogramming sensu stricto

"that reflects an expected" was added **line 219**.

l. 232: there are many more splicing factors than the SR proteins. These just represent on particularly well studied group.

Text has been modified accordingly. **line 253**

l. 273: Looks to me that SRSF1 is not THE top RBP in the list. By eyeballing hnRNPU and hnRNPA1 show higher scores overall in Fig 7A

SpliceLearn uses available ENCODE eCLIP data and supporting eCLIP peaks to predict RBPs binding, see **figure 7A** in the manuscript. The top predicted RBPs binding our targets were HNRNPU, QKI, HNRNPC and SRSF1. Furthermore, using FIMO from the MEME suite confirmed that the most common binding motifs belonged to SRSF1 (**see Supp Fig. 7A**) which was also captured by our OPP approach contrary to hnRNPU and hnRNPA1, for the following reasons we investigated further the role of SRSF1.

Figs 2G and S2C: depict the same data just plotted differently. One can be omitted entirely

We agree that both panels present the same dataset. However, one displays absolute values while the other shows relative percentages, which provide complementary perspectives on the data.

Figs 3c and D: essentially similar results, one panel could move into the supplement

We moved 3D in **Supp Fig. 3D.**

Fig 5A: I am truly puzzled by the model put forward by the authors...

Details have been added to the legend.

Fig S2D: This is not a sashimi plot, it's read depth plot, only

The figure has been updated with read depth plot.

Fig S2F: mentioned in legend but missing

It has been removed from the legend.

REVIEWERS' COMMENTS

Reviewer #1 (Remarks to the Author):

I think the authors responded well to my initial comments. I think that we already have enough acronyms for major processes and I dont see really a need for more acronyms. On the other hand, I see that many authors like to come up with new acronyms for effects they discovered. ERi-splice is acceptable and less misleading than the original name. As for the knockdown efficiency, 70% is still not a very strong knockdown, and a knockout should have been attempted. However, in light of the otherwise very strong evidence, I think the manuscript is as a whole very convincing.

We thank the reviewer for their thoughtful comments. We did consider a knockout approach; however, due to the three-month revision deadline, we opted for an siRNA-based strategy to ensure timely resubmission of the manuscript.

Additionally, we considered that siRNA presented a lower risk of adaptive responses or counter-selection compared to CRISPR-based knockout or lentiviral shRNA approaches for this specific question.

Reviewer #2 (Remarks to the Author):

I appreciate the effort that the authors undertook to revise the manuscript according to my suggestions. It really improved. I have no further comments.

We thank the reviewer for their positive feedback and for their valuable suggestions, which have helped to further strengthen and clarify the manuscript. In particular, the addition of experiments, such as the puromycin pull-down, SRSF1 Western blot with cycloheximide (CHX), the genotoxic, heat shock, hypoxia experiments and rescue experiments using ISRIB, that provides additional support for our conclusions.

Reviewer #3 (Remarks to the Author):

I commend the authors for the thorough revision of the manuscript and the additional data that strengthen the claims in the manuscript. Importantly, the authors now provide data that demonstrate that the identified splice signature can be induced in various different cell lines (Figure S3E & F) and that it is stress type specific: heat shock, hypoxia, genotoxic drugs, or amino acid starvation do not recapitulate the alternative splicing changes observed in the four critical transcripts after induction of the UPR (Figure S4D-F).

Is the splicing pattern truly UPR-specific or part of the ISR?

The authors provide additional evidence that the observed alternative splicing patterns of PRR3, RBM39, AP3S2, and ARIH2 are driven by stress-induced phosphorylation of eIF2alpha by PERK. Notably, knock down of the PERK downstream target NRF2 has no effect, while treatment with ISRIB mitigates the changes to alternative splicing (note to the authors: ISRIB treatment does not relieve Tm-induced ER stress [p25 of the rebuttal] but overcomes the effect of low to moderate phosphorylation of eIF2alpha).

What remains to be addressed is, however, whether other stress kinases that also phosphorylate eIF2alpha S51 can induce similar changes to alternative splicing. Unfortunately, the authors do not show whether GCN2 has been activated after withdrawal of glutamine from the tissue culture medium (Fig S4F) since eIF2 phosphorylation has not been experimentally addressed. Before claiming that the observed splicing signature is indeed ER stress-specific (as suggested by the name ERi-splice) it needs to be convincingly demonstrated that the activation of the other eIF2 kinases does not result in similar changes to splicing (hence not part of the ISR). Specific activation of other eIF2 kinases (GCN2, HRI, PKR, and MARK2) by e.g. pI:pC (PKR), BTdCPU (HRI) or the use of salubrinal would solve this issue.

I do not consider these experiments critical for publication, though. To get semantics right, the authors could instead of ERi-splice simply choose a different acronym that does not imply that the changes to AS are specific to activation of the UPR.

We thank Reviewer #3 for their thoughtful comments and suggestions regarding the need for a systematic approach to disentangle the contribution of the integrated stress response (ISR) to this splicing program.

In our study, several lines of evidence support the specificity of a PERK-driven response at early time points following stress. First, we employed a genetic inducible overexpression system for PERK. Second, the observed phenotype is reversed by pharmacological inhibition of PERK within 2 hours. Third, glutamine starvation, which activates GCN2, does not recapitulate the same splicing phenotype at 4 hours. Together, these findings argue for a specific and rapid PERK-dependent effect.

That said, we cannot exclude the possibility that, over longer time courses, activation of other ISR kinases such as PKR, or HRI could lead to a similar phenotype or induce distinct splicing programs. Addressing this question in a systematic manner, across different cell types, stimuli, and time points, would be an important direction for future investigation.
