# Supplementary material for: PERK orchestrates an endoplasmic reticulum stress alternative splicing program via CLK1/SRSF1
Source: Nat Commun. 2026 Jun 19;17:7745. doi: 10.1038/s41467-026-74397-y (PMC13434015; doi:10.1038/s41467-026-74397-y)
Supplement: Supplementary file 2 [file 41467_2026_74397_MOESM1_ESM.pdf]

Supplementary Figure 1

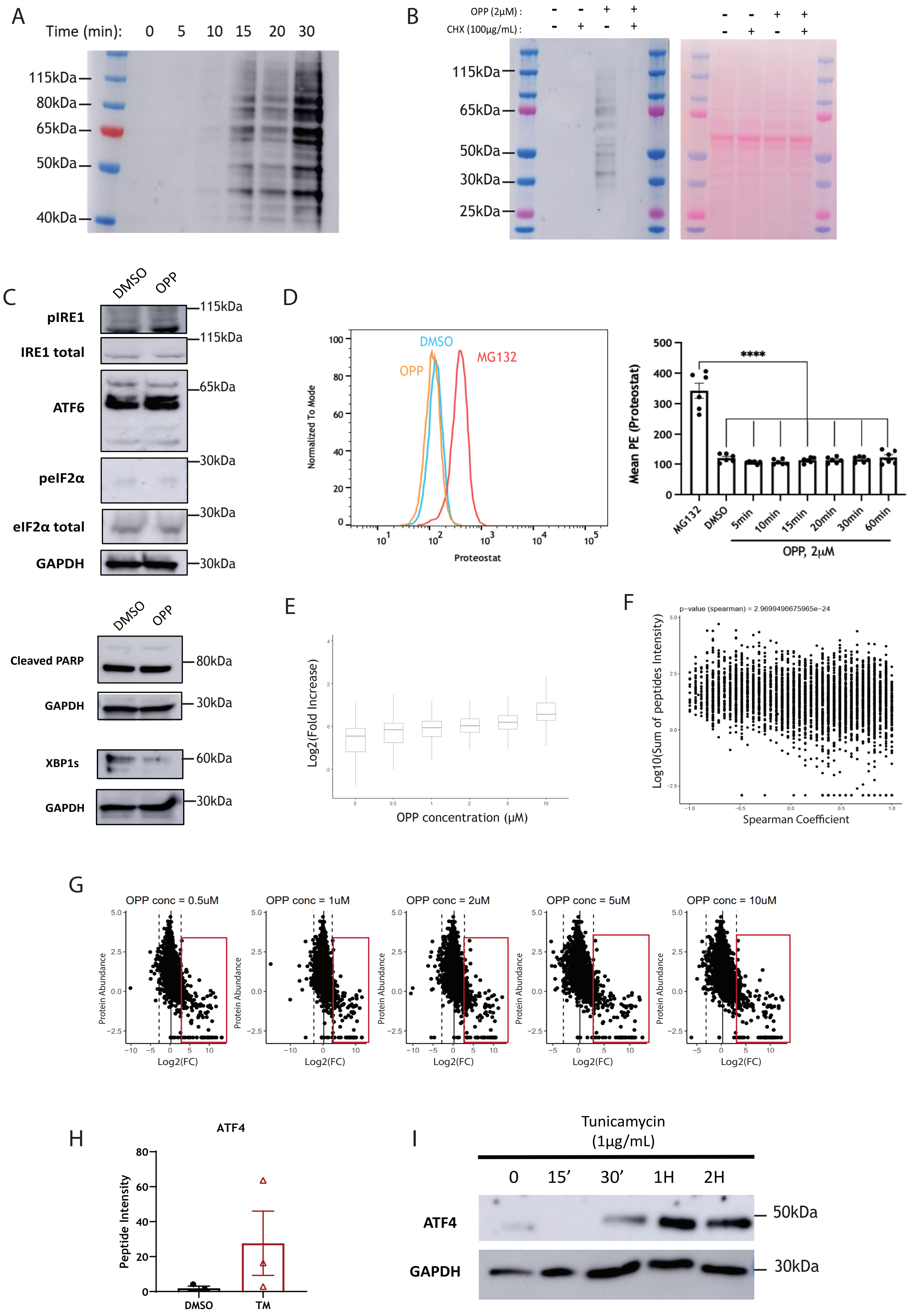

Supplementary Figure 1

J

ID	Gene Name	Spearman coefficient
BCLF1_HUMAN	BCL2 associated transcription factor 1(BCLAF1)	1
DDX49_HUMAN	DEAD-box helicase 49(DDX49)	1
DDX50_HUMAN	DExD-box helicase 50(DDX50)	1
RRP44_HUMAN	DIS3 homolog, exosome endoribonuclease and 3'-5' exoribonuclease(DIS3)	1
LSM8_HUMAN	LSM8 homolog, U6 small nuclear RNA associated(LSM8)	1
MOV10_HUMAN	Mov10 RNA helicase(MOV10)	1
EXOS8_HUMAN	exosome component 8(EXOSC8)	1
XPO5_HUMAN	exportin 5(XPO5)	1
HNRC1_HUMAN	heterogeneous nuclear ribonucleoprotein C like 1(HNRNPCL1)	1
HNRDL_HUMAN	heterogeneous nuclear ribonucleoprotein D like(HNRNPDL)	1
RUXGL_HUMAN	small nuclear ribonucleoprotein polypeptide G pseudogene 15(SNRPGP15)	1
DDX10_HUMAN	DEAD-box helicase 10(DDX10)	0.942857143
DDX18_HUMAN	DEAD-box helicase 18(DDX18)	0.942857143
DX39A_HUMAN	DExD-box helicase 39A(DDX39A)	0.942857143
G3BP1_HUMAN	G3BP stress granule assembly factor 1(G3BP1)	0.942857143
QKI_HUMAN	QKI, KH domain containing RNA binding(QKI)	0.942857143
SMCA4_HUMAN	SWI/SNF related, matrix associated, actin dependent regulator of chromatin, subfamily a, member 4(SMARCA4)	0.942857143
THOC3_HUMAN	THO complex subunit 3(THOC3)	0.942857143
THOC5_HUMAN	THO complex subunit 5(THOC5)	0.942857143
RENT1_HUMAN	UPF1 RNA helicase and ATPase(UPF1)	0.942857143
YTDC2_HUMAN	YTH N6-methyladenosine RNA binding protein C2(YTHDC2)	0.942857143
CSTFT_HUMAN	cleavage stimulation factor subunit 2 tau variant(CSTF2T)	0.942857143
IF4A3_HUMAN	eukaryotic translation initiation factor 4A3(EIF4A3)	0.942857143
EXOSX_HUMAN	exosome component 10(EXOSC10)	0.942857143
FUBP1_HUMAN	far upstream element binding protein 1(FUBP1)	0.942857143
GEMI5_HUMAN	gem nuclear organelle associated protein 5(GEMIN5)	0.942857143
HNRPR_HUMAN	heterogeneous nuclear ribonucleoprotein R(HNRNPR)	0.942857143
JMJD6_HUMAN	jumonji domain containing 6, arginine demethylase and lysine hydroxylase(JMJD6)	0.942857143
MBNL1_HUMAN	muscleblind like splicing regulator 1(MBNL1)	0.942857143
PTCD3_HUMAN	pentatricopeptide repeat domain 3(PTCD3)	0.942857143
SRSF1_HUMAN	serine and arginine rich splicing factor 1(SRSF1)	0.942857143
SRP54_HUMAN	signal recognition particle 54(SRP54)	0.942857143
TRM2A_HUMAN	tRNA methyltransferase 2 homolog A(TRMT2A)	0.942857143
SF3A3_HUMAN	Splicing Factor 3a Subunit 3(SF3A3)	0.942857143
SF3A2_HUMAN	Splicing Factor 3a Subunit 2(SF3A2)	0.942857143
CNOT7_HUMAN	CCR4-NOT transcription complex subunit 7(CNOT7)	0.885714286
ELAV1_HUMAN	ELAV like RNA binding protein 1(ELAVL1)	0.885714286
RBM12_HUMAN	RNA binding motif protein 12(RBM12)	0.885714286
THUM1_HUMAN	THUMP domain containing 1(THUMPD1)	0.885714286
RO52_HUMAN	tripartite motif containing 21(TRIM21)	0.885714286

K

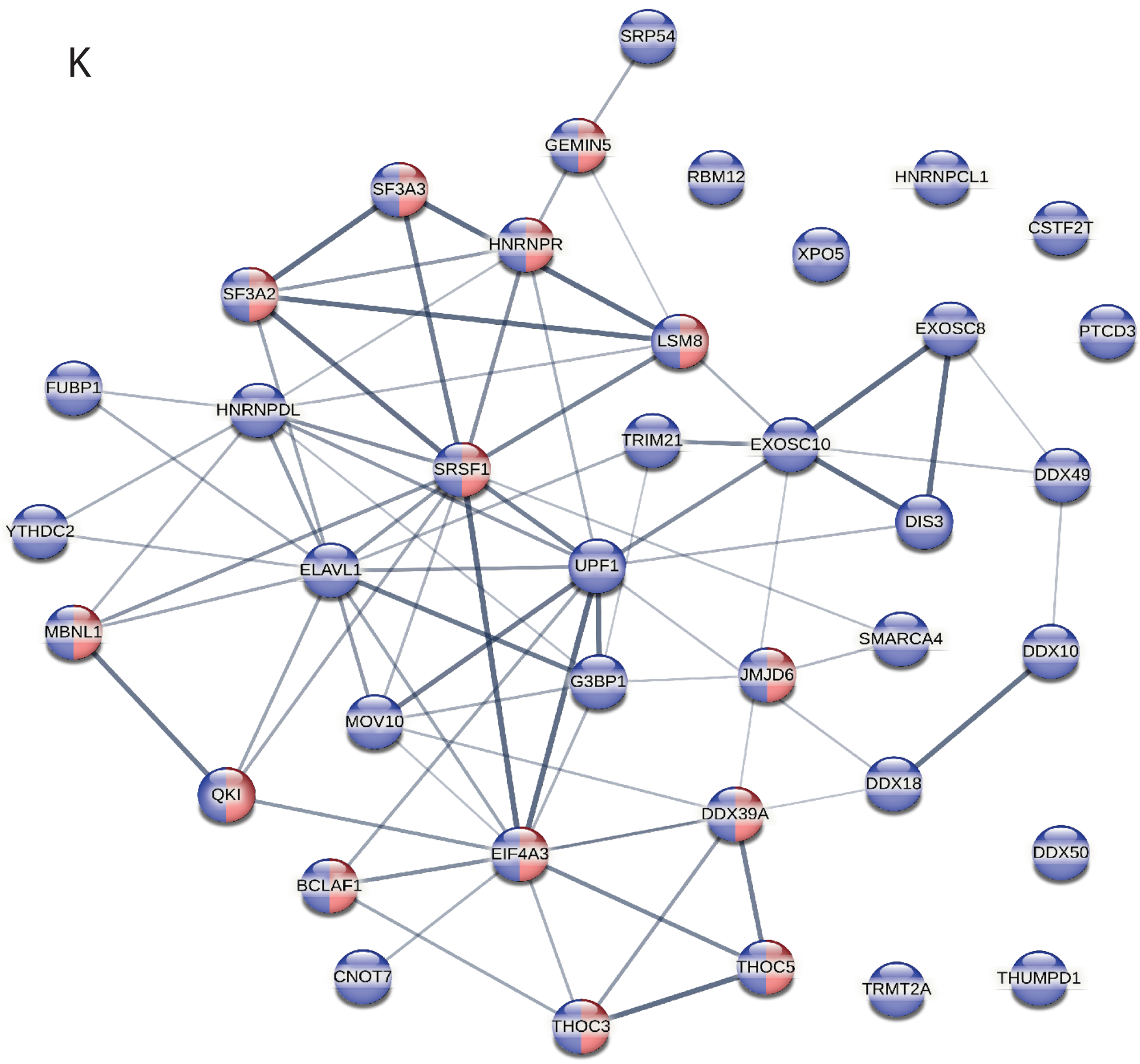

Supplementary Figure 1

L

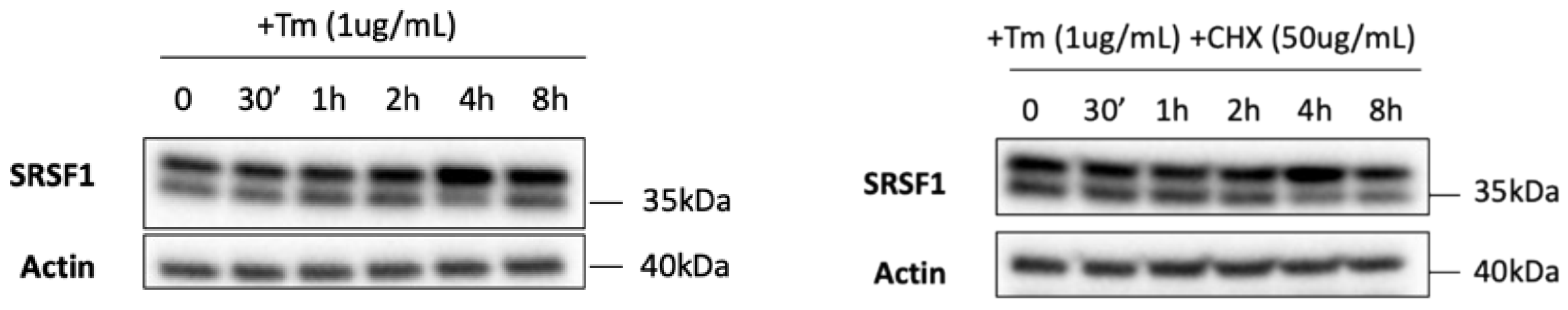

M

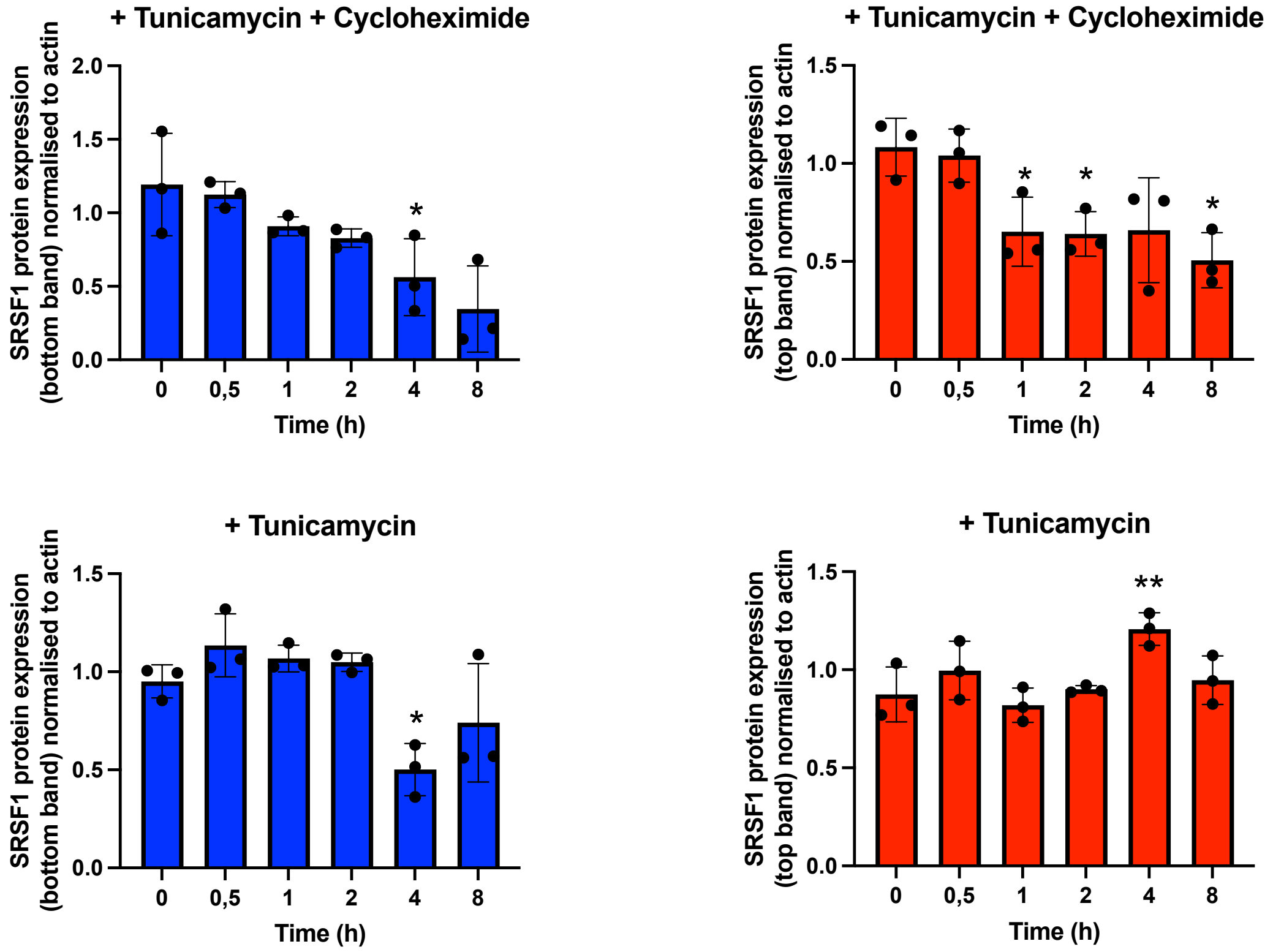

N

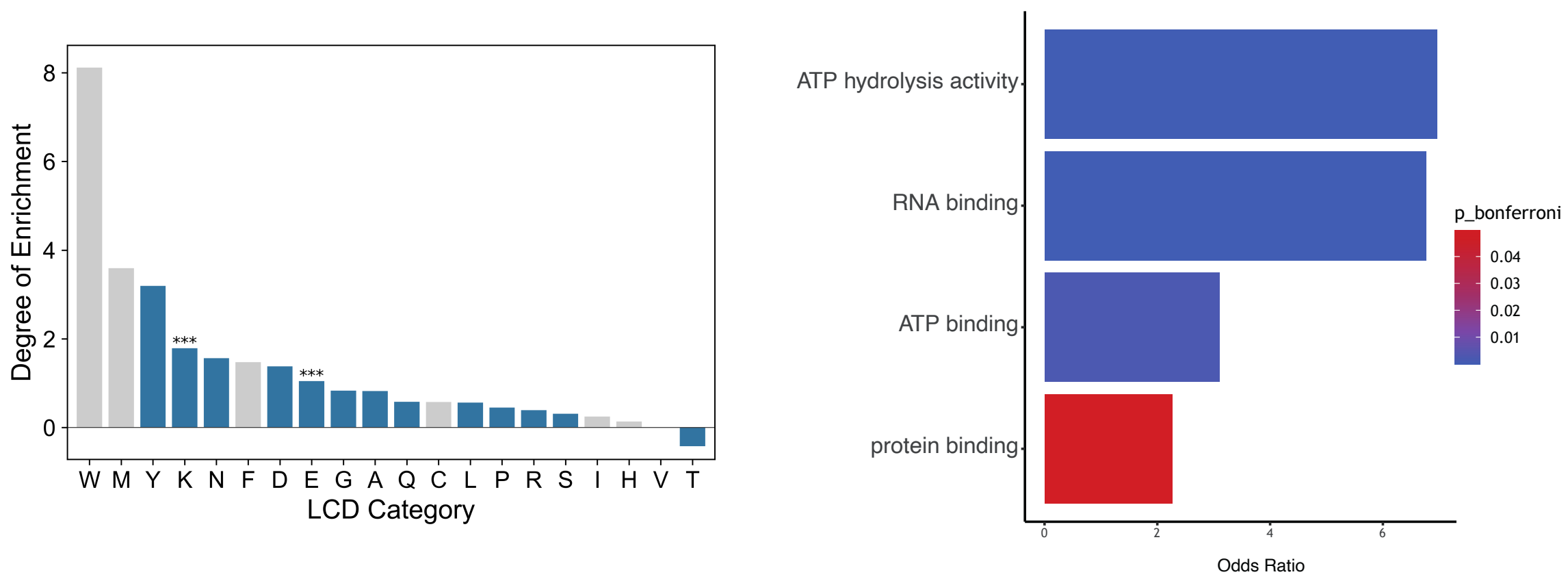

Supplementary Figure 1: OPP pull-down method allows identification of low abundant de novo peptides

(A) Sunset Assay showing *de novo* peptides tagged with 2µM puromycin for 0, 5, 10, 15, 20 and 30min in OCI-AML3 cells. (B) Sunset Assay (left panel) showing *de novo* peptides tagged with 2µM OPP and treated or not with 100µg/mL cycloheximide for 30min in OCI-AML3 cells. Ponceau staining of the same membrane depicts the total proteins loaded (right panel). (C) Western blot of phospho-IRE1, IRE1 total, ATF6, phospho-eIF2α, eIF2α total (top panel), and Cleaved PARP and XBP1s (bottom panel) in OCI-AML3 cells treated with 2µM OPP for 30min. GAPDH protein is used as a loading control. (D) Representative flow cytometry analysis of cells stained for aggresomes using the proteostat probe (left panel). Cells are either treated with DMSO, 2µM of OPP for 30min, or 10µM MG132 for 18H as a positive control. Mean of fluorescence has been reported for cells treated either with 10µM MG132 for 18H, or 2µM of OPP for 5, 10, 15, 20, 30, 60min and stained for aggresomes by using the proteostat probe (right panel). (n=4 biological replicates). mean± S.D. two-sided unpaired t.test. **** *p<0.01.* (E) Box plots showing the Log2(Fold Increase) of peptides according to the OPP concentration in OCI-AML3 treated with tunicamycin at 1µg/mL for 4H. (F) Graph depicting the correlation between peptides' intensity detected upon DMSO (log10(sum of basal)) and their spearman coefficient in the OPP pull down dose response experiment. (G) Plots displaying peptides' abundance according to their Log2(Fold Change (tunicamycin vs DMSO)) for each OPP concentration used. Captured peptides are contained in the red rectangle. (H) ATF4 peptide intensity detected following OPP pull-down in OCI-AML3 cells treated with tunicamycin at 1µg/mL for 4H and co-treated for the last 30min with 2µM OPP. (I) Western blot of ATF4 in OCI-AML3 cells treated with tunicamycin at 1µg/mL for 0, 15min, 30min, 1H and 2H. GAPDH protein is used as a loading control. (J) List of *de novo* peptides captured by OPP pull down with an RNA binding function. (K) Network shows interactions between RNA binding protein (in blue) synthesised upon UPR activation. Protein involved in splicing are highlighted in red. (L) Representative western blot of SRSF1 performed on OCI-AML3 treated with 1ug/mL tunicamycin +/- cycloheximide, for 0, 30min, 1H, 2H, 4H, and 8H. Actin is used as loading control. (M) Quantification of the protein expression level of SRSF1 (bottom band quantification in blue and top band quantification in red) normalised to Actin at the different time point and for the different conditions. (n=3 biological replicates). mean± S.D. two-sided unpaired t.test. **p<0.05,* ** *p<0.01.* (N) Enrichment plot in low-complexity domain in proteins captured by OPP pull down assay (top panel). The LCD-composer webserver was used for analysis. Gene Ontology analysis showing molecular functions enriched for the proteins with LCD and captured by OPP pull down assay (bottom panel). *adj.pvalue* <0.05.

Supplementary Figure2

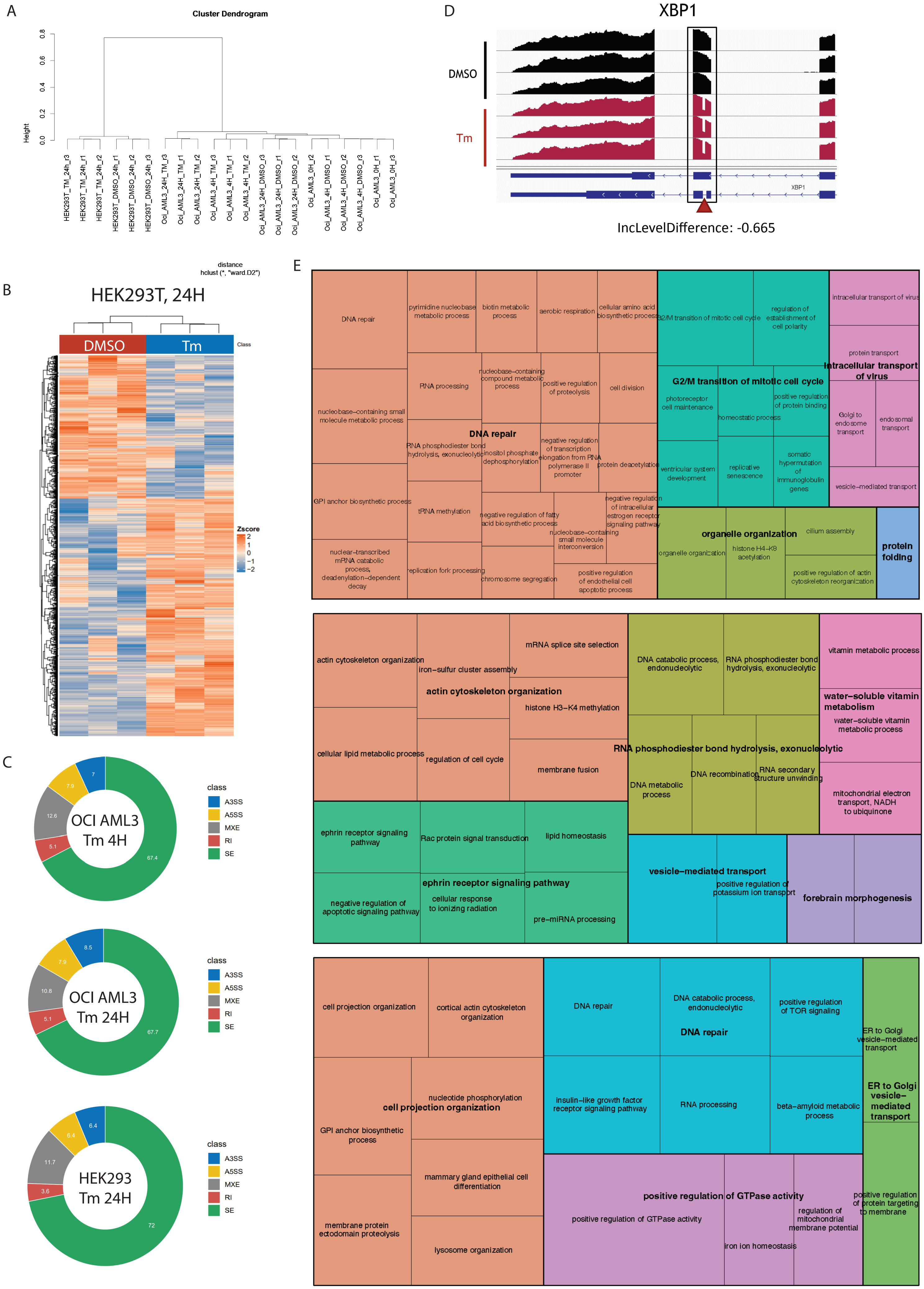

Supplementary Figure 2: Alternative splicing analysis reveals differential splicing events upon ER stress

(A) Unsupervised clustering of the samples analysed by RNAseq using all genes. (B) Heatmap of inclusion levels of differential splicing events in HEK293T cells treated with DMSO (red cluster) or 1µg/mL tunicamycin (blue cluster) for 24H. *FDR<0.05 and Inclusion Level Difference≥|0.1|*. (C) Pie chart representing the differential splicing events in OCI-AML3 cells treated with 1µg/mL tunicamycin compared to DMSO at 4H (top panel) and 24H (middle panel), and in HEK293T treated with 1µg/mL tunicamycin compared to DMSO at 24H (bottom panel). Splicing events are categorised as: skipped exon (SE), alternative 5' splice site (A5SS), alternative 3' splice site (A3SS), mutually exclusive exon (MXE), and retained intron (RI). *FDR<0.05 and Inclusion Level Difference≥|0.1|*. (D) Read depth plot depicting the reads count aligned on XBP1 gene for the samples treated with DMSO (in black) and treated with 1µg/mL tunicamycin for 4H (in red). The black rectangle highlights the "unconventional splicing" undergoing upon ER stress. (E) REVIGO analysis of genes identified as differentially spliced upon tunicamycin compared to DMSO in OCI-AML3 cells treated for 4H (top panel), for 24H (middle panel) and in HEK293T cells treated for 24H (bottom panel). *FDR<0.05 and Inclusion Level Difference≥|0.1|*. The significantly enriched GO Biological processes with *adj. pvalue < 0.05* are used as input in REVIGO.

Supplementary Figure 3

A

Samples	GeneID	geneSymbol	chr	strand	exonStart	exonEnd	PValue	FDR	IncLevelDifference
OCI AML3 4h	ENSG00000204576	PRR3	chr6	+	30561327	30561508	0	0	0.395
OCI AML3 24h	ENSG00000204576	PRR3	chr6	+	30561327	30561508	1.76E-05	0.001905	0.178
HEK293T 24h	ENSG00000204576	PRR3	chr6	+	30561327	30561508	3.87E-08	2.20E-05	0.161
OCI AML3 4h	ENSG00000131051	RBM39	chr20	-	35740524	35740597	0	0	0.222
OCI AML3 24h	ENSG00000131051	RBM39	chr20	-	35740524	35740597	1.64E-07	5.69E-05	0.108
HEK293T 24h	ENSG00000131051	RBM39	chr20	-	35740524	35740597	1.11E-16	5.53E-13	0.128
OCI AML3 4h	ENSG00000157823	AP3S2	chr15	-	89877305	89877424	3.21E-09	1.01E-06	0.34
OCI AML3 24h	ENSG00000157823	AP3S2	chr15	-	89877305	89877424	7.38E-07	0.000183	0.199
HEK293T 24h	ENSG00000157823	AP3S2	chr15	-	89877305	89877424	9.67E-05	0.007448	0.323
OCI AML3 4h	ENSG00000177479	ARIH2	chr3	+	48945135	48945181	3.47E-10	1.42E-07	0.15
OCI AML3 24h	ENSG00000177479	ARIH2	chr3	+	48945135	48945181	1.37E-07	4.89E-05	0.358
HEK293T 24h	ENSG00000177479	ARIH2	chr3	+	48945135	48945181	1.28E-09	1.34E-06	0.654

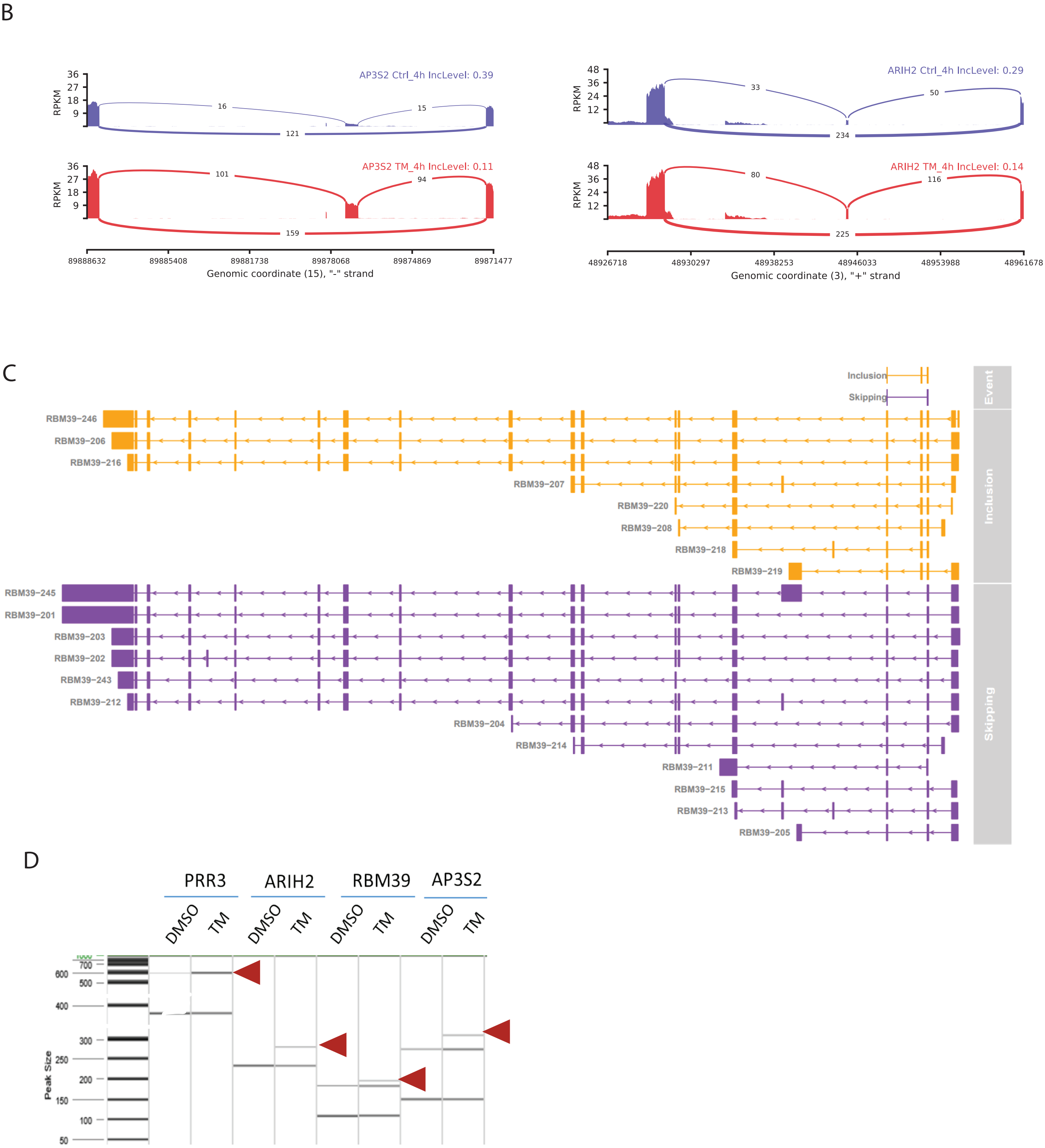

Supplementary Figure 3

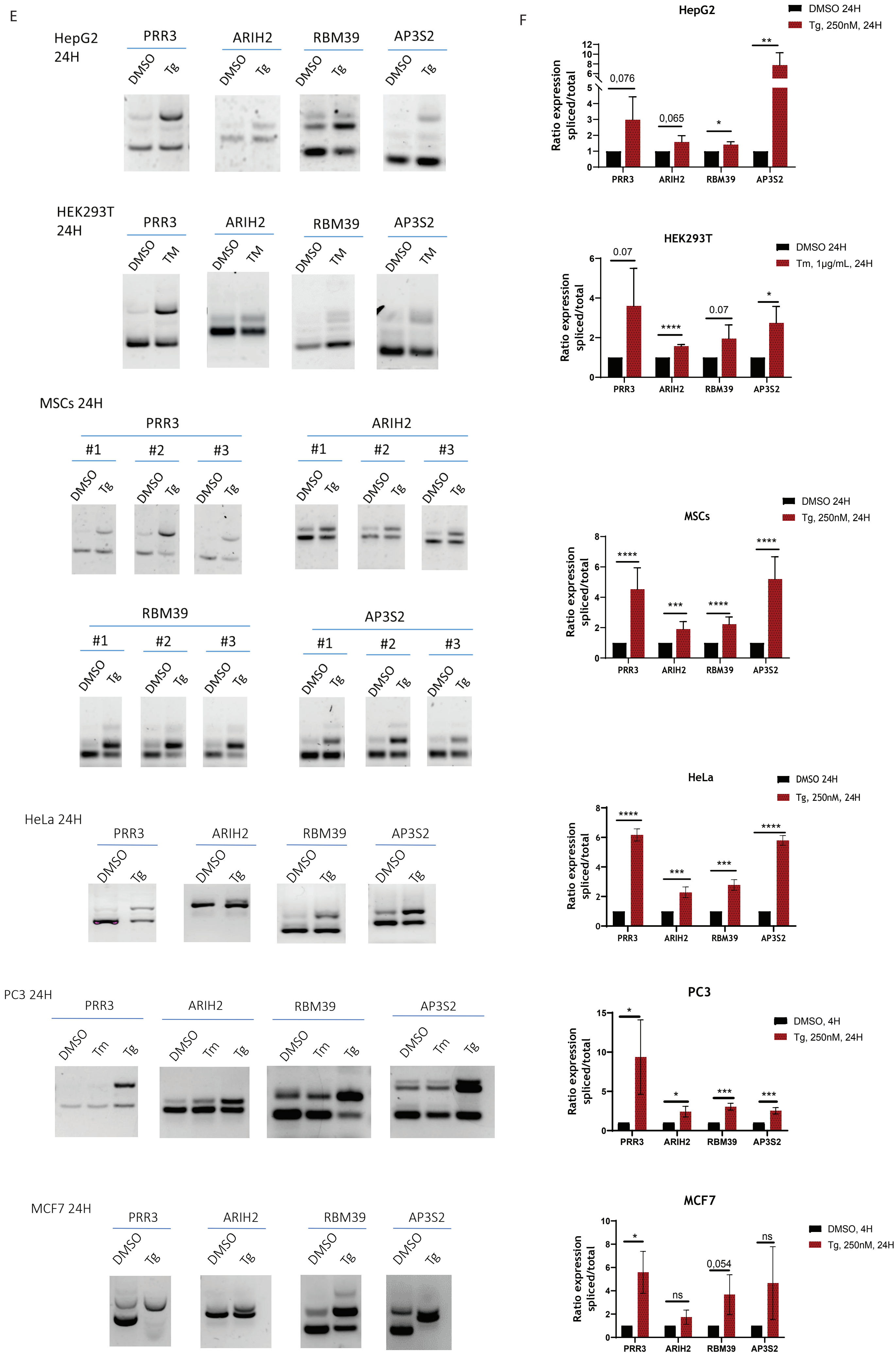

Supplementary Figure 3: Identification of isoforms that accumulate upon ER stress

(A) List of the four alternative splicing events identified by rMATS (*FDR<0.05 and Inclusion Level Difference≥|0.1|*) as common to OCI-AML3 cells treated for 4H and 24H, and to HEK293T cells treated for 24H of tunicamycin at 1µg/mL. (B) Sashimi plots of two splicing events: *AP3S2* (left panel) and *ARIH2* (right panel), in OCI-AML3 treated with DMSO (in Red) or with 1µg/mL tunicamycin (in Blue) for 4H. (C) Representation of RBM39 isoforms that include (in yellow) or exclude (in purple) the exon of interest. (D) Representative PCR analysed with QIAxcel showing alternative splicing occurring in OCI-AML3 treated with 1µg/mL tunicamycin for 4H, for four of the splicing events: *PRR3, ARIH2, RBM39 and AP3S2.* (E) Gel of PCR products showing the alternative splicing events occurring in HepG2, HEK293T, Mesenchymal Stem Cells isolated from three healthy donors (#1,#2,#3), HeLa, PC3 and MCF7 cells treated with tunicamycin at 1µg/mL or thapsigargin at 250nM for 24H, for four of the splicing events: *PRR3, ARIH2, RBM39 and AP3S2.* (F) Quantitative PCR of four alternative splicing events (*PRR3, ARIH2, RBM39 and AP3S2)* occurring in HepG2, HEK293T, Mesenchymal Stem Cells isolated from three healthy donors (#1,#2,#3), HeLa, PC3 and MCF7 cells treated with tunicamycin at 1µg/mL or thapsigargin at 250nM for 24H. Data here are normalized respectively to total *PRR3, ARIH2, RBM39 and AP3S2* (n=3 biological replicates). mean± S.D. two-sided unpaired t.test. * *p<0.05*, ** *p<0.01*, ****p<0.005*, *****p<0.001.*

Supplementary Figure 4

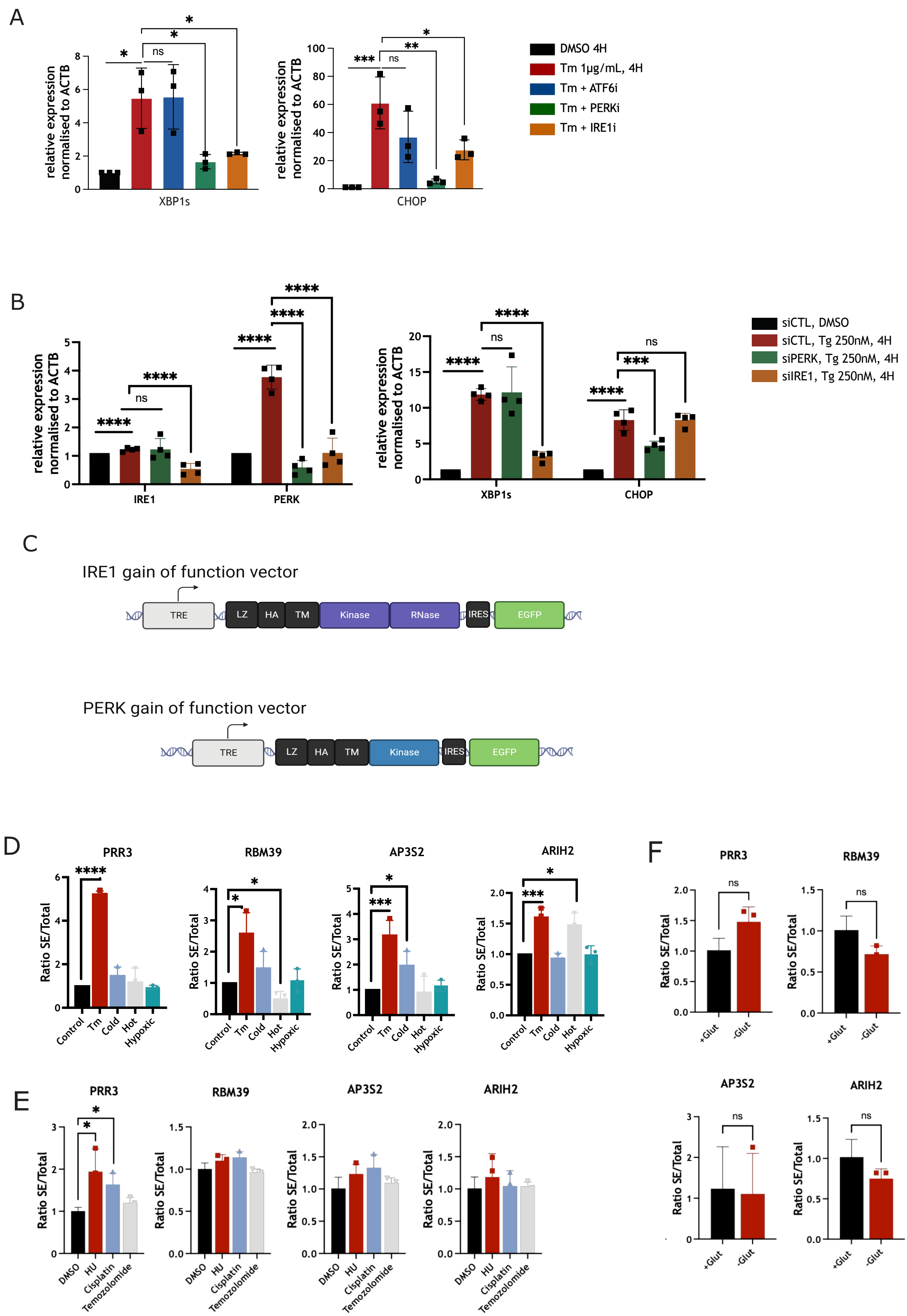

Supplementary Figure 4: Validation and description of the ER stress investigation tools

(A) Quantitative PCR analysis of *XBP1s* (left panel) and *CHOP* (right panel) mRNA in OCI-AML3 cells treated with 1µg/mL tunicamycin for 4H +/- PERK, ATF6 or IRE1 inhibitor, respectively used at 300nM, 10µM, and 50µM. Data are normalized to Actin B (n=3 biological replicates). mean± S.D. two-sided unpaired t.test. * *p<0.05, ** p<0.01, ***p<0.005*. (B) Quantitative PCR analysis of *IRE1, PERK* (left panel), and *XBP1s, CHOP* (right panel) mRNA in OCI-AML3 cells that have been transfected with either siRNA control (CTL), siRNA PERK, or siRNA IRE1 for 48H, and then treated with 1µg/mL tunicamycin for 4H. Data are normalized to Actin B (n=4 biological replicates). mean± S.D. two-sided unpaired t.test. * *p<0.05, ** p<0.01, ***p<0.005, ****p<0.001.* (C) Schematic representation of the two gain-of-function vectors of the UPR branches: IRE1 (top panel) and PERK (bottom panel). The intraluminal ER sensor domain of PERK and IRE1 is replaced by a c-Jun leucine zipper (LZ) fused to a HA tag (PERK-LZ, IRE1-LZ). The transmembrane domain (TM) and cytosolic domains (Kinase and RNAse) remain intact compared to wild type IRE1 and PERK. A cassette IRES-EGFP has been cloned downstream of the recombinant proteins to allow cell selection by flow cytometry. A doxycycline-inducible promoter (TRE) controls the entire gene. Created in BioRender. Philippe, C. (2026) https://BioRender.com/818ef4i (D) Quantitative PCR of four alternative splicing events (*PRR3, ARIH2, RBM39 and AP3S2)* occurring in OCI-AML3 following ER stress (tunicamycin, 1µg/mL, 4H), Heat Shock either cold (34°C, 4h) or heat (42°C, 4h), and Hypoxia (1%O2, 4h). Data are normalized respectively to total *PRR3, ARIH2, RBM39 and AP3S2* (n=3 biological replicates). mean± S.D. two-sided unpaired t.test. **p<0.05*, ****p<0.005*, *****p<0.001.* (E) Quantitative PCR of four alternative splicing events (*PRR3, ARIH2, RBM39 and AP3S2)* occurring in OCI-AML3 cells treated with genotoxic compounds (500 µM hydroxyurea, 3µM cisplatin or 50µM temozolomide) for 24H. Data are normalized respectively to total *PRR3, ARIH2, RBM39 and AP3S2* (n=3 biological replicates). mean± S.D. two-sided unpaired t.test. **p<0.05.* (F) Quantitative PCR of four alternative splicing events (*PRR3, ARIH2, RBM39 and AP3S2)* occurring in OCI-AML3 cells cultured +/- glutamine for 4H. Data are normalized respectively to total *PRR3, ARIH2, RBM39 and AP3S2* (n=3 biological replicates). mean± S.D. **p<0.05*, ****p<0.005.*

Supplementary Figure 5

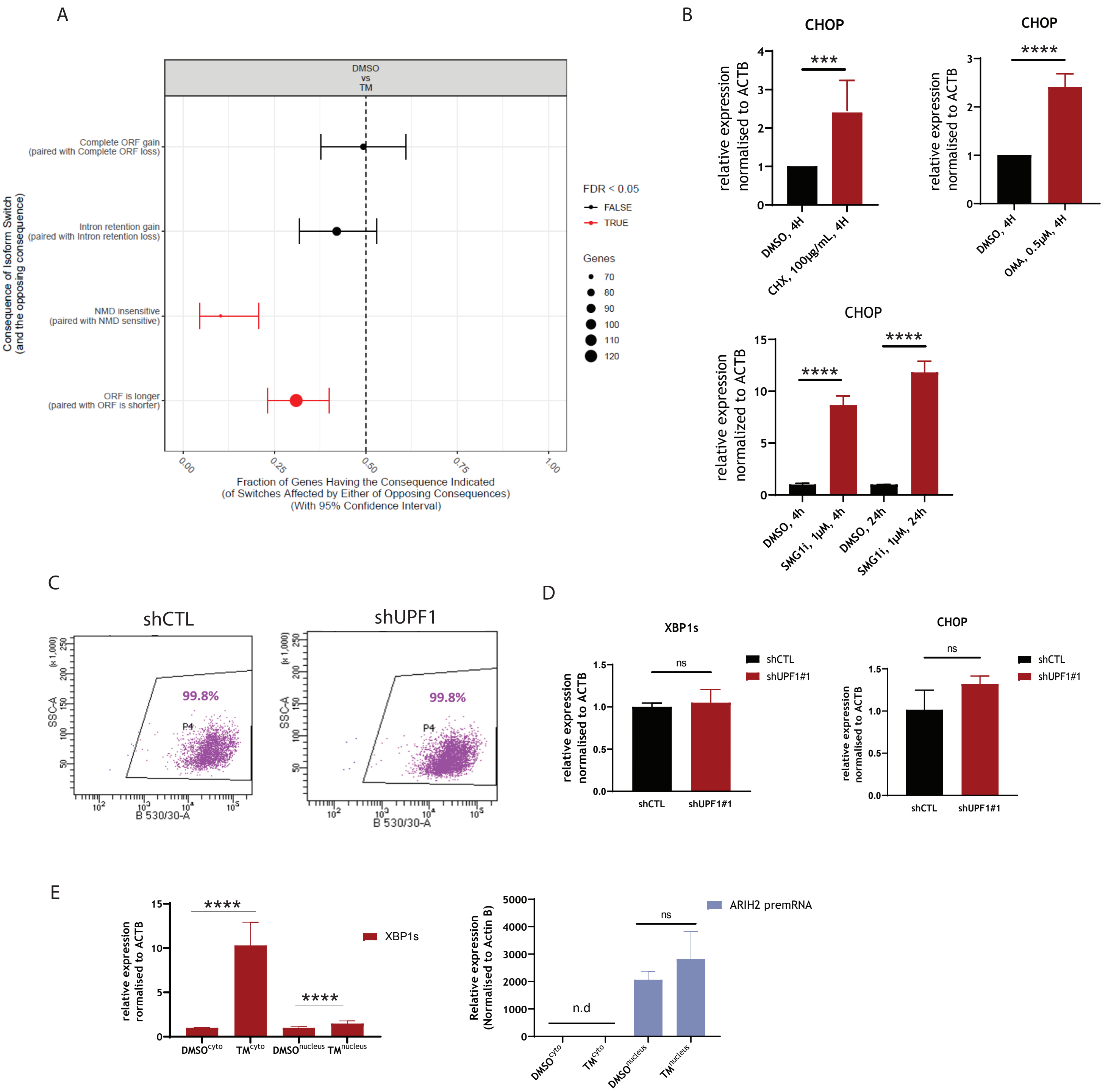

Supplementary Figure 5: Uncoupling NMD and ER stress' effects

(A) Representation of the consequences of isoform switches detected with the IsoformSwitchAnalyzeR package, in OCI-AML3 treated with 1µg/mL tunicamycin for 4H. (B) Quantitative PCR of *CHOP* in OCI-AML3 treated with either 100µg/mL cycloheximide (CHX) for 4H (left panel) (n=5 biological replicates), 0.5µM omacetaxine (OMA) for 4H (right panel) (n=6 biological replicates), or 1µM SMG1 inhibitor for 4 or 24H (bottom panel) (n=3 biological replicates). Data are normalized to Actin B. mean± S.D. two-sided unpaired t.test. ** $p<0.01$, **** $p<0.001$. (C) Representative dot plots depicting the percentage of EGFP cells in OCI-AML3 transduced with the shControl, shCTL, (left panel) or shUPF1#1 (right panel). (D) Quantitative PCR of *XBP1s* (left panel) and *CHOP* (right panel) in OCI-AML3 transduced with the shControl (shCTL) or shUPF1#1. Data are normalized to Actin B (n=3 biological replicates). mean± S.D. two-sided unpaired t.test. (E) Quantitative PCR of *XBP1s* (left panel), as a cytosolic control, and *ARIH2* pre-mRNA (right panel), as a nuclear control, in cytoplasmic or nuclear fractions of OCI-AML3 treated with 1µg/mL tunicamycin for 4H. Data here are normalized respectively to Actin B (n=3 biological replicates). mean± S.D. two-sided unpaired t.test. **** $p<0.001$.

Supplementary Figure 6

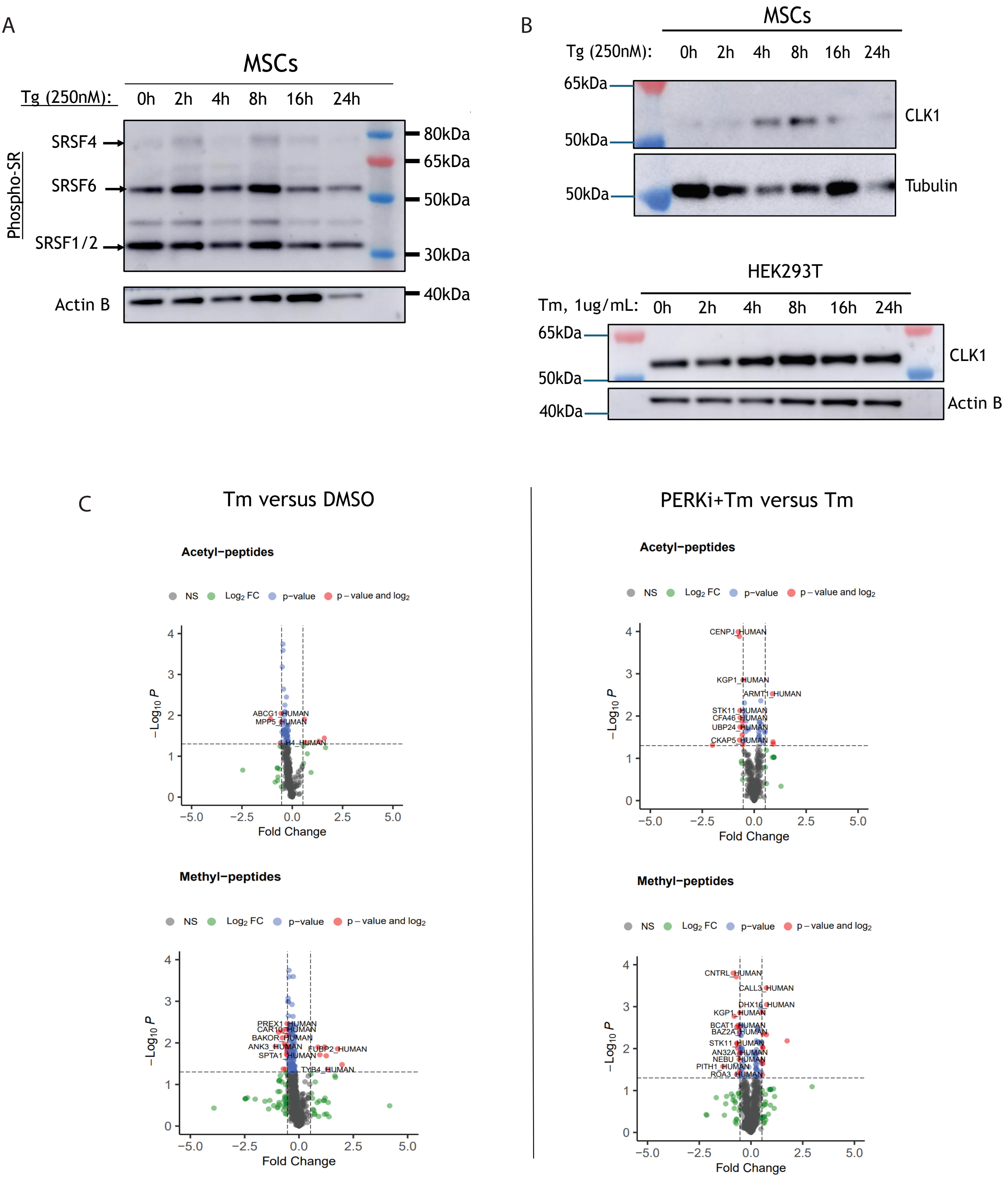

Supplementary Figure 6

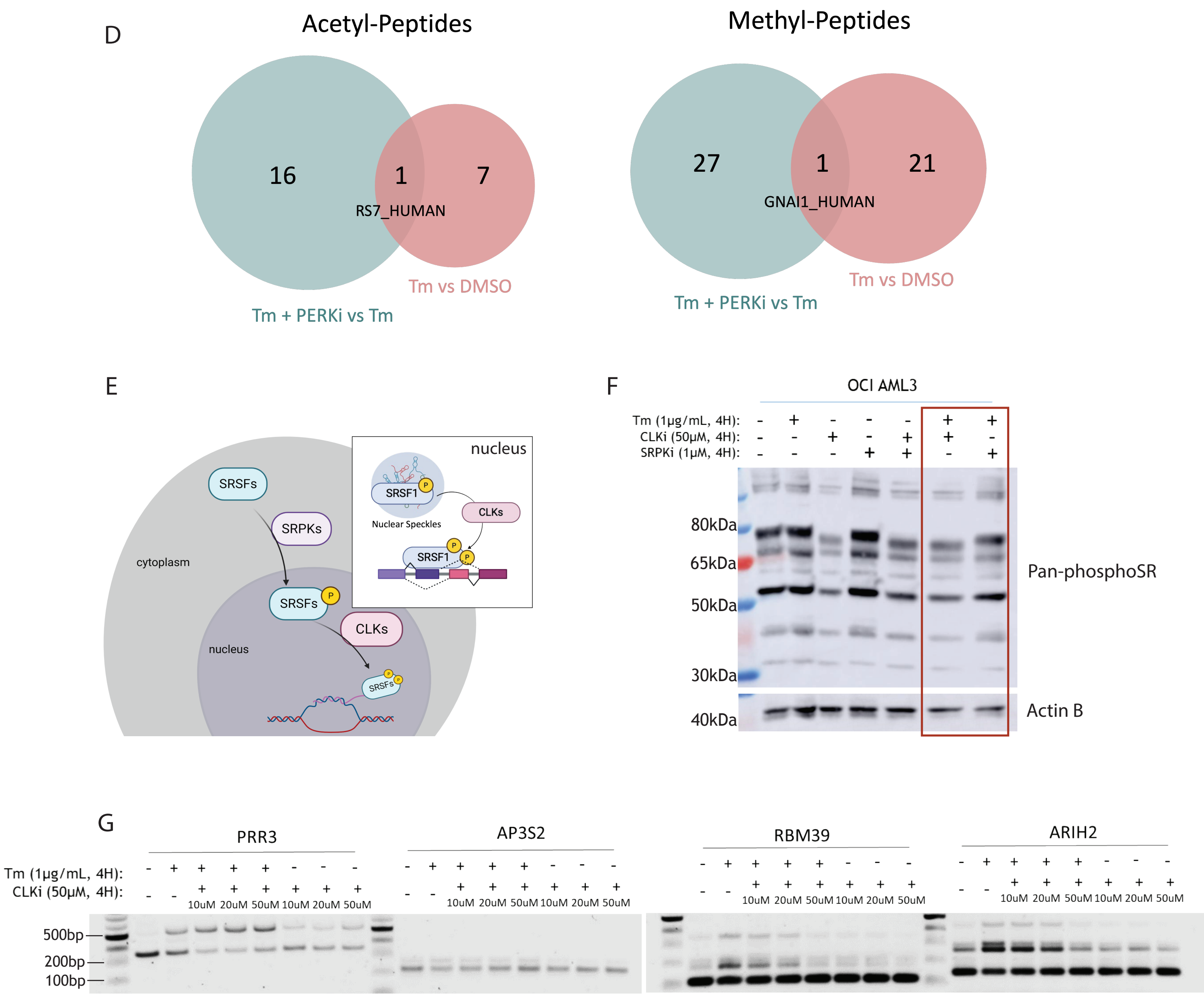

Supplementary Figure 6: Post-translational modifications upon ER stress

(A) Representative western blot of phospho-SR proteins in Mesenchymal Stem Cells (MSCs) cells treated with 250nM thapsigargin for 0, 2, 4, 8, 16 and 24H. Actin B protein is used as a loading control. (B) Representative western blot of CLK1 protein expression levels in Mesenchymal Stem Cells (MSCs) cells (top panel), and CLK1 in HEK293T cells (bottom panel) treated, respectively with 250nM thapsigargin or 1µg/mL tunicamycin for 0, 2, 4, 8, 16 and 24H. Tubulin and Actin are used as loading control. (C) Volcano plots depicting fold change in acetyl- (top panel) and methyl-peptides (bottom panel), detected by LC-MS/MS, in OCI-AML3 treated for 4H with 1µg/mL tunicamycin (Tm) versus DMSO (left panel), and tunicamycin and PERK inhibitor (PERKi) versus cells treated with tunicamycin only (right panel). (D) Venn diagrams showing the overlap in acetyl-peptides (left panel) or methyl-peptides (right panel) significantly affected in both analysis, Tm versus DMSO, and Tm+PERKi versus Tm, in OCI-AML3 cells for 2H. (E) Schematic depicts the role of the kinases SRPKs, in the cytoplasm, and CLKs, in the nucleus, in phosphorylating SRSFs factors. Created in BioRender. Philippe, C. (2026) https://BioRender.com/2ke7imd (F) Western blot of phospho-SR proteins in OCI-AML3 cells treated with 1µg/mL tunicamycin (Tm), with or without 50µM of CLK inhibitor (CLKi) or 1µM SRPK inhibitor (SRPKi) for 4H. Actin B protein is used as a loading control. (G) PCR gel showing four alternative splicing events (*PRR3, ARIH2, RBM39 and AP3S2)* occurring in OCI-AML3 treated with tunicamycin at 1µg/mL, with or without CLK inhibitor (CLKi) at 10µM, 20µM or 50 µM for 4H.

Supplementary Figure7

A

33 common events

FIMO scanning

All of those common events have a predicted **SRSF1** binding sites

	RBP id	Motif id	Matched sequence	p-value
1	SRSF1	RNCMPT00110	CAGGACAC	1.28E-05
2	SRSF2	RNCMPT00072	AGGAGAGG	1.41E-05
3	KHDRBS3	RNCMPT00034	GATAAACC	1.90E-05
4	TARDBP	RNCMPT00076	TGAATGAG	1.92E-05
5	ZCRB1	RNCMPT00087	GAATTAAG	2.36E-05
6	SRSF7	RNCMPT00073	GACGACGA	2.40E-05
7	FXR1	RNCMPT00161	AATGACAA	2.41E-05
8	RBM8A	RNCMPT00056	GCGCGCG	3.00E-05
9	PPRC1	RNCMPT00045	GCGCGCG	3.00E-05
10	RBM4	RNCMPT00052	GCGCGGG	3.10E-05

B

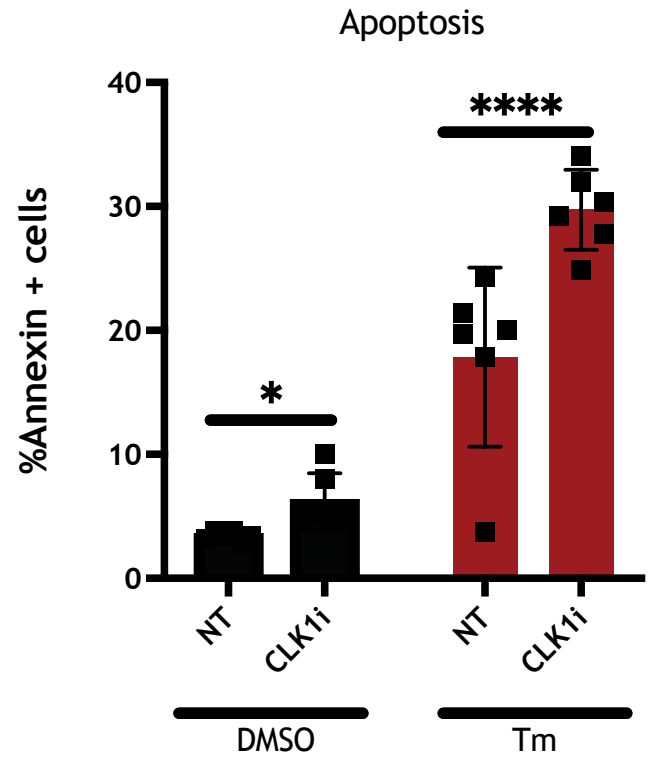

C

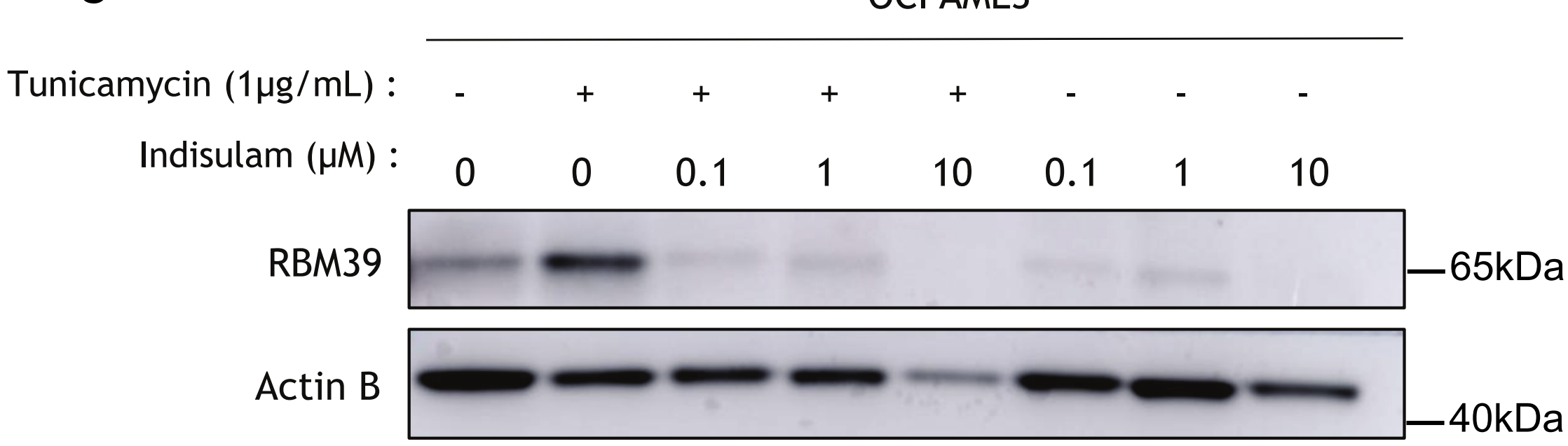

D

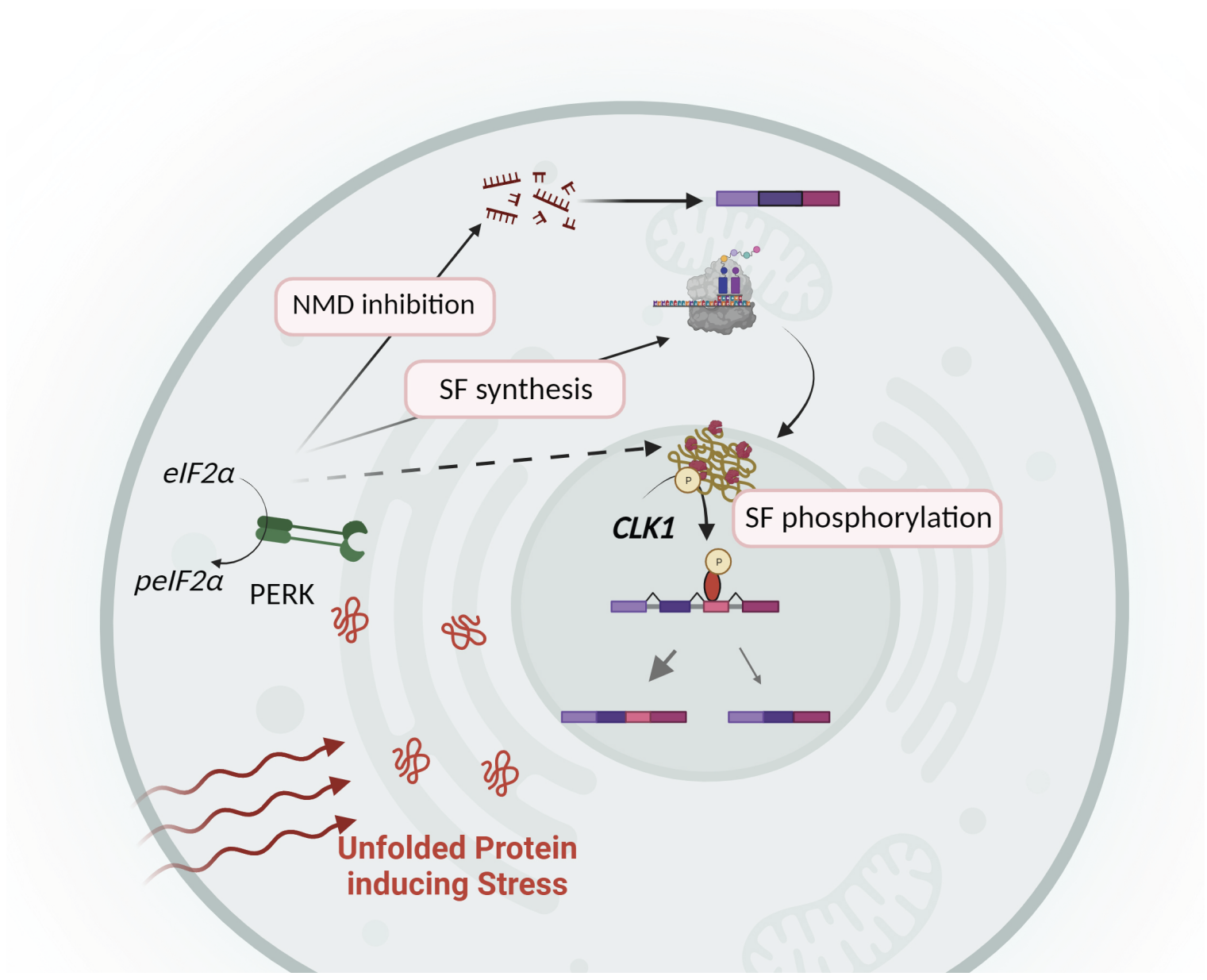

Supplementary Figure 7: RBM39 splicing and inhibition

(A) FIMO analysis performed on extracted sequences from the 33 common splicing events described in fig3. Sequences of the skipped exon and spanning introns and exons were retrieved and scanned for RNA Binding Protein motifs. Top 10 motifs found in the 33 sequences are displayed. (B) Apoptosis assessed by Annexin V staining in OCI AML3 treated with or without tunicamycin (Tm) at 1µg/mL, and with or without CLK1 inhibitor, TG003 (CLKi) at 50µM for 24H. (n=3 biological replicates, with technical duplicates displayed). mean± S.D. two-sided unpaired t.test. ***$p<0.01$.* (C) Western blot of RBM39 in OCI-AML3 cells treated with or without tunicamycin at 1µg/mL, and with or without indisulam at 0.1µM, 1µM or 10µM for 48H. Actin protein is used as a loading control. (D) Graphical abstract depicting the different processes affecting isoforms changes during ER stress: Non-sense mediated decay (NMD) inhibition, Splicing factor (SF) synthesis, and Splicing factor (SF) phosphorylation. Created in BioRender. Philippe, C. (2026) https://BioRender.com/cngm363.
