# Supplementary material for: PERK orchestrates an endoplasmic reticulum stress alternative splicing program via CLK1/SRSF1
Source: Nat Commun. 2026 Jun 19;17:7745. doi: 10.1038/s41467-026-74397-y (PMC13434015; doi:10.1038/s41467-026-74397-y)
Supplement: Supplementary file 3 [file 41467_2026_74397_MOESM2_ESM.pdf]

Description of Additional Supplementary Files

Supplementary Data 1: OPP-captured peptides' list
For the OPP pull-down experiment, a Spearman correlation coefficient was calculated for each protein by ranking peptide intensities and OPP concentrations from smallest to largest. Proteins with a Spearman coefficient ≥ 0.8 were then shortlisted.

Supplementary Data 2: $ER^{i\text{-}splice}$ genes list of 33 and 8
Differential splicing events were thresholded with FDR =< 0.05 and inclusion level difference >= |0.1|. Common splicing events found at the intersection of the different models (*Figure 3A and 4G*).

Supplementary Data 3: Phosphoproteomic at 24h of tunicamycin
For phosphoproteomics experiments, we obtained a phosphorylation index (ppIndex) by summing the signals of all peptide ions containing the same modification site. Normalised intensity for the phosphopeptides, in OCI-AML3 cells treated were analysed by two-sided unpaired t.test.

Supplementary Data 4: Phosphoproteomic at 2h of tunicamycin and PERK inhibitor
For phosphoproteomics experiments, we obtained a phosphorylation index (ppIndex) by summing the signals of all peptide ions containing the same modification site. Normalised intensity for the phosphopeptides, in OCI-AML3 cells treated were analysed by two-sided unpaired t.test.

Supplementary Data 5: Post-translational modification analysis at 24h of tunicamycin
For post translational modifications experiments, normalised intensity for the modified peptides, in OCI-AML3 cells treated were analysed by two-sided unpaired t.test.

Supplementary Data 6: Primers' list
