# Supplementary material for: PERK orchestrates an endoplasmic reticulum stress alternative splicing program via CLK1/SRSF1
Source: Nat Commun. 2026 Jun 19;17:7745. doi: 10.1038/s41467-026-74397-y (PMC13434015; doi:10.1038/s41467-026-74397-y)
Supplement: Supplementary file 4 [file 41467_2026_74397_MOESM9_ESM.pdf]

nature portfolio

Corresponding author(s): Dr Kevin Rouault-Pierre

Last updated by author(s): Nov 1, 2025

Reporting Summary

Nature Portfolio wishes to improve the reproducibility of the work that we publish. This form provides structure for consistency and transparency in reporting. For further information on Nature Portfolio policies, see our Editorial Policies and the Editorial Policy Checklist.

Statistics

For all statistical analyses, confirm that the following items are present in the figure legend, table legend, main text, or Methods section.

n/a	Confirmed	
☐	☒	The exact sample size (*n*) for each experimental group/condition, given as a discrete number and unit of measurement
☐	☒	A statement on whether measurements were taken from distinct samples or whether the same sample was measured repeatedly
☐	☒	The statistical test(s) used AND whether they are one- or two-sided<br>*Only common tests should be described solely by name; describe more complex techniques in the Methods section.*
☐	☒	A description of all covariates tested
☐	☒	A description of any assumptions or corrections, such as tests of normality and adjustment for multiple comparisons
☐	☒	A full description of the statistical parameters including central tendency (e.g. means) or other basic estimates (e.g. regression coefficient) AND variation (e.g. standard deviation) or associated estimates of uncertainty (e.g. confidence intervals)
☐	☒	For null hypothesis testing, the test statistic (e.g. *F*, *t*, *r*) with confidence intervals, effect sizes, degrees of freedom and *P* value noted<br>*Give P values as exact values whenever suitable.*
☒	☐	For Bayesian analysis, information on the choice of priors and Markov chain Monte Carlo settings
☒	☐	For hierarchical and complex designs, identification of the appropriate level for tests and full reporting of outcomes
☐	☒	Estimates of effect sizes (e.g. Cohen's *d*, Pearson's *r*), indicating how they were calculated

Our web collection on statistics for biologists contains articles on many of the points above.

Software and code

Policy information about availability of computer code

Data collection	FACSDiva™ Software BD Biosciences<br>Amersham 600 RGB imager (chemidoc)<br>BioRad CFX Manager
Data analysis	Image J Schneider et al. https://imagej.nih.gov/ij/<br>GraphPad Prism Pad Software, Inc.<br>Serial Cloner SerialBasics Software http://serialbasics.free.fr/Home/Home.html<br>rMAT Shen et al. https://rnaseq-mats.sourceforge.io/<br>GSEA Subramanian et al. https://www.gsea-msigdb.org/gsea/index.jsp<br>FlowJo™ Software BD Biosciences<br>IsoformSwitchAnalyzeR Kristoffer Vitting-Seerup et al. https://bioconductor.org/packages/release/bioc/html/IsoformSwitchAnalyzeR.html<br>MASER Veiga et al. https://bioconductor.org/packages/release/bioc/html/maser.html<br>IGV Robinson et al. https://igv.org/<br>SynergyFinder Plus Zheng et al. https://tangsoftwarelab.shinyapps.io/synergyfinder/<br>GSVA Castelo et al. https://www.bioconductor.org/packages/release/bioc/html/GSVA.html<br>survcomp Haibe-Kains et al. https://bioconductor.org/packages/release/bioc/html/survcomp.html<br>RSEM Li et al. http://deweylab.github.io/RSEM/<br>limma Ritchie et al. https://bioconductor.org/packages/release/bioc/html/limma.html<br>XCaliburg Thermo Fisher Scientific https://www.thermofisher.com/search/results?query=xcalibur%E2%84%A2&navId=12141&persona=Catalog

Mascot Daemon (v2.8.0.1) Matrix Science http://www.matrixscience.com/
Mascot Distiller (v2.5.1.0) Matrix Science http://www.matrixscience.com/
Mascot Search Engine (v2.5) Matrix Science http://www.matrixscience.com/
Pescal In house developed software https://doi.org/10.1074/mcp.M700037-MCP200
R (v3.6.1) The R project of Statistical Computing https://www.r-project.org/
RStudio Desktop (v1.2.5033) RStudio https://rstudio.com/
Gplots package (v3.0.3) cran r project https://cran.r-project.org/web/packages/gplots/index.html
ggplot2 package (v3.3.1) cran r project https://cran.r-project.org/web/packages/ggplot2/index.html
ggpubr package (v0.3.0) cran r project https://cran.r-project.org/web/packages/ggpubr/index.html
ComplexHeatmap Gu et al. https://bioconductor.org/packages/release/bioc/html/ComplexHeatmap.html
Maxquant (v1.6.3.3) Tyanova et al. https://www.maxquant.org
SpliceLearn™ Fronk et al. https://doi.org/10.1101/2022.10.14.512313

For manuscripts utilizing custom algorithms or software that are central to the research but not yet described in published literature, software must be made available to editors and reviewers. We strongly encourage code deposition in a community repository (e.g. GitHub). See the Nature Portfolio guidelines for submitting code & software for further information.

Data

Policy information about availability of data

All manuscripts must include a data availability statement. This statement should provide the following information, where applicable:

- Accession codes, unique identifiers, or web links for publicly available datasets
- A description of any restrictions on data availability
- For clinical datasets or third party data, please ensure that the statement adheres to our policy

The GDC Pan-cancer RNASeq dataset was downloaded from the UCSC Xena browser (GDC-PANCAN.htseq_fpkm-uq.tsv, n = 11,768 samples). Transcriptomics' and proteomics' datasets generated in this study have been uploaded respectively on GEO (GSE245918) and PRIDE (PXD046476). Data will be publicly accessible right after publication.

Research involving human participants, their data, or biological material

Policy information about studies with human participants or human data. See also policy information about sex, gender (identity/presentation), and sexual orientation and race, ethnicity and racism.

Reporting on sex and gender	NA
Reporting on race, ethnicity, or other socially relevant groupings	NA
Population characteristics	NA
Recruitment	NA
Ethics oversight	NA

Note that full information on the approval of the study protocol must also be provided in the manuscript.

Field-specific reporting

Please select the one below that is the best fit for your research. If you are not sure, read the appropriate sections before making your selection.

☒ Life sciences ☐ Behavioural & social sciences ☐ Ecological, evolutionary & environmental sciences

For a reference copy of the document with all sections, see nature.com/documents/nr-reporting-summary-flat.pdf

Life sciences study design

All studies must disclose on these points even when the disclosure is negative.

Sample size	For statistical relevance, each proteomics' experiments has been performed on 4 replicates. RNAseq and Functional in vitro experiments were performed on three independent biological replicates as stated in the figures' legend.
Data exclusions	No data excluded
Replication	The number of replicates (technical or biological) are stated in the figure legends. All findings have been replicated on different technical and biological replicates. To test the robustness of the results, we validated the findings on different cell types (primary and cell lines).
Randomization	NA

Blinding	NA

Reporting for specific materials, systems and methods

We require information from authors about some types of materials, experimental systems and methods used in many studies. Here, indicate whether each material, system or method listed is relevant to your study. If you are not sure if a list item applies to your research, read the appropriate section before selecting a response.

Materials & experimental systems

n/a	Involved in the study
☐	☒ Antibodies
☐	☒ Eukaryotic cell lines
☒	☐ Palaeontology and archaeology
☒	☐ Animals and other organisms
☒	☐ Clinical data
☒	☐ Dual use research of concern
☒	☐ Plants

Methods

n/a	Involved in the study
☒	☐ ChIP-seq
☐	☒ Flow cytometry
☒	☐ MRI-based neuroimaging

Antibodies

Antibodies used

Anti-Phospho-IRE1 (Ser724) Rabbit polyclonal Abcam #ab48187
Anti-IRE1α Rabbit monoclonal Cell Signaling #3294
Anti-XBP1s Rabbit polyclonal Cell Signaling #83418
Anti-cleaved PARP (Asp214) Rabbit polyclonal Cell Signaling #9541
Anti-Phospho-eIF2α (Ser51) Rabbit monoclonal Cell Signaling #3398
Anti-eIF2α Antibody Rabbit polyclonal Cell Signaling #9722
Anti-ATF6 Mouse monoclonal Novus Biologicals #NBP1-40256
Anti-Puromycin Mouse monoclonal Merck #MABE343
Anti-ATF4 Rabbit monoclonal Cell Signaling #11815
Anti-SRSF1 Mouse monoclonal Thermo Fisher #324500
Anti-RBM39 Mouse monoclonal Proteintech #67420-1-lg
Anti-AP3S2 Rabbit polyclonal Proteintech #15319-1-AP
Anti-Phosphoepitope SR proteins Mouse monoclonal Merck #MABE50
Anti-UPF1 Rabbit monoclonal Cell Signaling #12040
Anti-αTubulin Mouse monoclonal Abcam #ab7291
Anti-GAPDH Mouse monoclonal Cell Signaling #97166
Anti-Histone H3 Rabbit polyclonal Merck #PLA0148
Anti-Actin Rabbit polyclonal Merck #A2066
Anti-HA antibody Mouse monoclonal Cell Signaling #2999
Annexin V-Alexa Fluor 647 Biolegend #640912

Validation

All antibodies were validated by their suppliers.

Eukaryotic cell lines

Policy information about cell lines and Sex and Gender in Research

Cell line source(s)	OCI AML3 (DSMZ, male), HEK293T (DSMZ, female), PC3 (DSMZ, male), MCF7 (DSMZ, female), HeLa (DSMZ, female), HepG2 (DSMZ, male)
Authentication	Experiments have been performed on low passaged cell culture. Cell lines were authenticated by their suppliers.
Mycoplasma contamination	Cell lines were all tested negative for mycoplasma
Commonly misidentified lines (See ICLAC register)	NA

Plants

Seed stocks	NA
Novel plant genotypes	NA
Authentication	NA

Flow Cytometry

Plots

Confirm that:

☒ The axis labels state the marker and fluorochrome used (e.g. CD4-FITC).

☒ The axis scales are clearly visible. Include numbers along axes only for bottom left plot of group (a 'group' is an analysis of identical markers).

☒ All plots are contour plots with outliers or pseudocolor plots.

☒ A numerical value for number of cells or percentage (with statistics) is provided.

Methodology

Sample preparation	Cells were harvested and washed in PBS 1x prior to staining and were stained as described in the methods section
Instrument	Analysis was performed on the LSRFortessa (BD). Cell sorting was performed on a FACSAria Fusion (BD).
Software	DIVA was used to acquire the data and FLowJo was used for analysis
Cell population abundance	For transduced cells, GFP was >99% after cell sorting. For apoptosis assay, AnnexinV+ cells percentage depends of the treatment applied.
Gating strategy	DAPI neg cells were firstly selected (NA for apoptosis), then single cells were selected (SSC-H, SSC-A), Debris are removed (FSC-A, SSC-A). Populations of interest (GFP+ or Annexin V+) were then investigated.

☒ Tick this box to confirm that a figure exemplifying the gating strategy is provided in the Supplementary Information.
